# The evolution of hemocyanin genes in Tectipleura: a multitude of conserved introns in highly diverse gastropods

**DOI:** 10.1186/s12862-021-01763-3

**Published:** 2021-03-04

**Authors:** Gabriela Giannina Schäfer, Veronika Pedrini-Martha, Daniel John Jackson, Reinhard Dallinger, Bernhard Lieb

**Affiliations:** 1 Institute of Molecular Physiology, Johannes Gutenberg-University of Mainz, Johann-Joachim-Becher-Weg 7, 55128 Mainz, Germany; 2 Institute of Zoology and Center of Molecular Biosciences, University of Innsbruck, Technikerstraße 25, 6020 Innsbruck, Austria; 3 Department of Geobiology, Georg-August-University of Göttingen, Goldschmidtstr. 3, 37077 Göttingen, Germany

**Keywords:** Hemocyanin, Gene structure, Intron, Intron gain, Evolution, Habitat shift, Adaptation, Tectipleura, Heterobranchia, Mollusca

## Abstract

**Background:**

Hemocyanin is the oxygen transporter of most molluscs. Since the oxygen affinity of hemocyanin is strongly temperature-dependent, this essential protein needs to be well-adapted to the environment. In Tectipleura, a very diverse group of gastropods with > 27,000 species living in all kinds of habitats, several hemocyanin genes have already been analyzed. Multiple independent duplications of this gene have been identified and may represent potential adaptations to different environments and lifestyles. The aim of this study is to further explore the evolution of these genes by analyzing their exon–intron architectures.

**Results:**

We have reconstructed the gene architectures of ten hemocyanin genes from four Tectipleura species: *Aplysia californica, Lymnaea stagnalis, Cornu aspersum* and *Helix pomatia*. Their hemocyanin genes each contain 53 introns, significantly more than in the hemocyanin genes of Cephalopoda (9–11), Vetigastropoda (15) and Caenogastropoda (28–33). The gene structures of Tectipleura hemocyanins are identical in terms of intron number and location, with the exception of one out of two hemocyanin genes of *L. stagnalis* that comprises one additional intron. We found that gene structures that differ between molluscan lineages most probably evolved more recently through independent intron gains.

**Conclusions:**

The strict conservation of the large number of introns in Tectipleura hemocyanin genes over 200 million years suggests the influence of a selective pressure on this gene structure. While we could not identify conserved sequence motifs within these introns, it may be simply the great number of introns that offers increased possibilities of gene regulation relative to hemocyanin genes with less introns and thus may have facilitated habitat shifts and speciation events. This hypothesis is supported by the relatively high number of introns within the hemocyanin genes of *Pomacea canaliculata* that has evolved independently of the Tectipleura. *Pomacea canaliculata* belongs to the Caenogastropoda, the sister group of Heterobranchia (that encompass Tectipleura) which is also very diverse and comprises species living in different habitats. Our findings provide a hint to some of the molecular mechanisms that may have supported the spectacular radiation of one of Metazoa’s most species rich groups.

## Background

The Mollusca is the second largest animal phylum and comprises diverse species with an enormous array of different body forms, physiologies, habitats and behaviors. They are divided into eight major classes (e.g. Cephalopoda, Bivalvia and Scaphopoda) of which Gastropoda represents by far the largest. Gastropoda encompass the five major groups Patellogastropoda, Vetigastropoda, Neritimorpha, Caenogastropoda and Heterobranchia of which especially the latter two are highly diverse. Together they form the clade Apogastropoda [1] and include about 68,800 different species [2]. Species of both groups can be found in most kinds of environments and exhibit a wide range of specializations. Adaptations of their respiratory systems, for example, were fundamental for colonization of different habitats. Besides respiratory organs, also the oxygen transporter of molluscs, namely hemocyanin, must have been adapted to new environments because the oxygen affinity of this protein is known to be temperature-dependent [3–6]. Therefore, changes to molluscan hemocyanin genes must reflect an essential part of the process of adaptation to new habitats to sustain a sufficient oxygen supply [3, 4]. Accordingly, the evolution of this protein is strongly linked to the evolution of molluscs. Analyzing hemocyanin genes and reconstructing their evolution will therefore deepen our understanding of the evolution of the Mollusca.

**Fig. 1:**
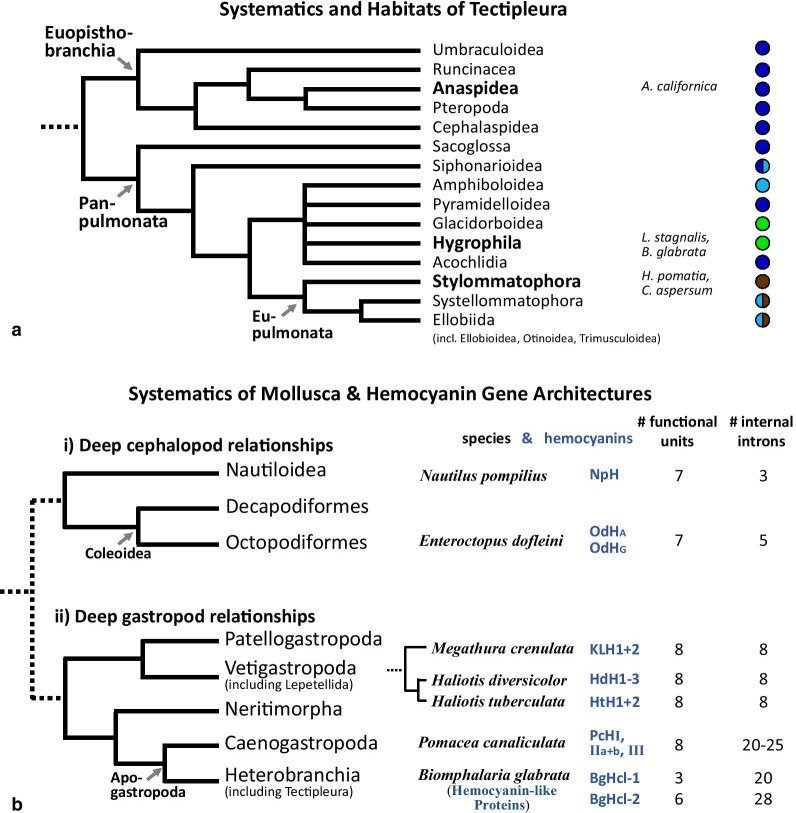
Systematics of Tectipleura, their habitats and hemocyanin gene structures. **a** Tectipleura comprise the two large groups Euopisthobranchia and Panpulmonata. The systematics of both groups are shown as derived in molecular analysis by Jörger et al*.* [7], Schrödel [8] and Kano et al*.* [9]. Tectipleura included in this study are shown on the right site of the lineage they belong to (highlighted in bold). Habitats are represented by colored circles on the right. While all Euopisthobranchia are marine (dark blue), Panpulmonata also include intertidal (light blue), limnic (green) and terrestrial (brown) gastropods. **b** The number of internal introns and of functional units (right) of yet analyzed cephalopod and gastropod hemocyanin genes. For the distribution within functional units see Additional file 1. Species and the rough systematics of the major groups they belong to are shown on the left side. Hemocyanin-like proteins of *Biomphalaria glabrata* are included in this figure, as well

In this study we focus on hemocyanins of Tectipleura, a group of Heterobranchia with over 27,000 species. This clade was recovered by molecular phylogenetic analyses [7–9] and comprises Euopisthobranchia (e.g. sea hares (Anaspidea) and pteropod sea butterflies) and Panpulmonata (e.g. traditional pulmonates as Hygrophila and Stylommatophora; Fig. 1a). They have undergone multiple independent habitat shifts from sea to land or freshwater within different clades, and have therefore developed independent adaptations. Within Panpulmonata, for example, the evolution of lungs took place several times independently [7, 10]. In a previous analysis, we have reported about hemocyanin gene duplications that occurred multiple times independently within different Panpulmonata lineages [11]. Most probably, those duplication events represent convergent adaptations to their multiple habitat shifts and may have supported the extensive radiation of Tectipleura. Thus, the aim of this study was to analyze the genes of hemocyanins within Tectipleura in more detail.

**Fig. 2:**
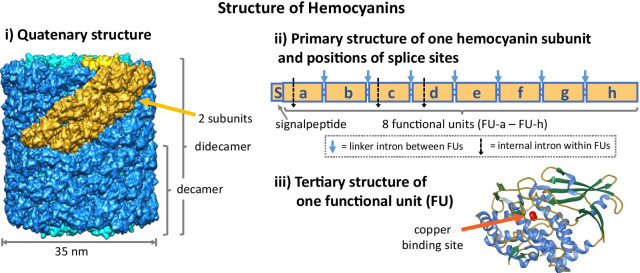
Basic structure of molluscan hemocyanin. On protein level, hemocyanin represents a partly hollow cylinder (outer wall: dark blue; inner collar: light blue, PDB-ID: 4BED) with a diameter of 35 nm (**i**). This quaternary structure is built out of two decamers lying on top of each other. The repetitive unit is a subunit dimer colored in golden (collar: yellow). The primary structure of one subunit (**ii**) shows the composition of one signal peptide (S) and eight paralogous domains called functional units (FU-a – FU-h). Each functional unit forms a tertiary structure as shown in (**iii**) and comprises a copper binding site with two copper ions that can bind a dioxygen molecule reversibly (PDB-ID: 1JS8). Within the primary structure (**ii**) also splice sites are illustrated: blue arrows symbolize splice sites of linker introns that lie between the FUs (conserved at the same position and in phase 1 for all analyzed hemocyanins); dotted black arrows symbolize splice sites of internal introns. Their number and positions vary among the different gene structures

Comprising approximately 3400 amino acids, the 400 kDa subunits of molluscan hemocyanins belong to the largest naturally occurring polypeptides (Fig. 2). Mono-, di- and multidecamers of these proteins are freely floating in the hemolymph and cause its blue color due to their binuclear copper active site. In several molluscan species, two or more hemocyanin genes have been found [12–14]. Previous studies identified the basic composition of hemocyanin subunits to be highly conserved [15]: Usually, each subunit comprises eight homologous protein domains termed functional units FU-a to FU-h (Fig. 2) which are connected by linker peptides of 10–20 amino acids. This eight-FU protein most probably evolved from a single-FU gene precursor through three domain duplications in a common ancestor of all molluscs before the radiation into different molluscan classes [16]. This repetitive structure in combination with the multitude of hemocyanin genes within many molluscan species impedes de novo assemblies of NGS (Next Generation Sequencing) data. Thus, automatically assembled hemocyanin gene or cDNA sequences are often error-prone. To analyze these genes which partially reflect the evolution of molluscs (see above), however, additional studies like this one are required.

The highly conserved and complex structure of hemocyanins is also reflected by the structure of their genes: In all molluscan hemocyanin genes that have been analyzed so far the encoded individual functional units are separated from each other by phase 1 introns (called linker introns) which lie at an almost equivalent point just upstream from the linker peptide coding regions (Fig. 2) [16–18]. Thereby, intron phases 0, 1 and 2 are defined as being located before the first, after the first and after the second nucleotide of a codon, respectively. Consequently, every FU is represented by at least one separate exon. The identical positions across all molluscan classes suggest that those introns originated through domain duplications which resulted in the first hemocyanin consisting of eight functional units [16]. Thus, these introns are most probably as old as the hemocyanin molecule itself.

Beyond these linker introns between the functional units, Lieb et al*.* [16] identified introns lying within these FUs, subdividing them into multiple exons. The number of these introns as well as their position within the sequence and their intron phases vary between hemocyanin genes of different molluscan lineages and between different functional units. To distinguish between those different kinds of introns within hemocyanin genes, they have been classified in two types called (i) linker introns (phase 1 introns located between single functional units) and (ii) internal introns (non-conserved introns of any phase within any functional unit, see Fig. 2 and [16]).

Full hemocyanin exon–intron architectures are known from the cephalopods *Enteroctopus dofleini* [16] and *Nautilus pompilius* [18] as well as of three species of Vetigastropoda (*Megathura crenulata, Haliotis tuberculata and H. diversicolor* [16, 17, 19, 20]). Figure 1b shows that hemocyanin gene structures differ between hemocyanins of these different groups. Hemocyanin genes within these groups and within the same species, on the other hand, are identical in terms of number and positions of introns (see also Additional file 1). The internal introns of hemocyanin genes most probably originated independently after the ancestors of these groups separated from each other (e.g. for the listed Vetigastropoda in a common ancestor of Lepetellida, the vetigastropod group to which *Megathura* and *Haliotis* belong to).

Only recently, Chiumiento et al*.* [21] showed that exon–intron structures of four hemocyanin genes of the caenogastropod *Pomacea canaliculata* differ not only from those of Lepetellida but also vary from each other. Furthermore, they contain a much larger number of introns than those known from Vetigastropoda or Cephalopoda (Fig. 1b). Since no other hemocyanins of Caenogastropoda are known yet, it is unclear whether the multitude of introns in hemocyanin genes of *P. canaliculata* is species-specific or widespread within that clade.

Apart from Lepetellida and *P. canaliculata*, no gastropod exon–intron architectures of full-length hemocyanin genes have yet been described. Peña and Adema [22], however, published the exon–intron architecture of two hemocyanin-like genes of *Biomphalaria glabrata*. This species belongs to Planorbidae, hygrophilid panpulmonates within Tectipleura which represents the only known gastropod family that uses hemoglobin (evolved from a gastropod myoglobin) instead of hemocyanin as their primary oxygen transporter [23]. Despite this, hemocyanin relics of *B. glabrata* have been detected in electron microscope analyses and by SDS-PAGEs by Lieb et al*.* [23] (hemocyanin without the inner collar). Furthermore, two genes that encode partial, incomplete hemocyanin-like proteins have been identified [22]: One (*BgHcl-1*) consisting of three FUs (FU-a, FU-b and FU-h), the other one (*BgHcl-2*) comprising six FUs (FU-a to FU-f). The gene architectures of both genes comprise the typical phase 1 linker introns between distinct functional units, leading to the conclusion that these genes may indeed be evolutionary remnants of previous full-length hemocyanin genes. Similar to the hemocyanin genes of *P. canaliculata*, both *BgHcl* genes contain a conspicuously larger number of internal introns per FU than those of Lepetellida and Cephalopoda (Fig. 1b). Since the exact number and the positions of internal introns in *BgHcl* are different to those in hemocyanins of *P. canaliculata*, the gene structures must have evolved independently within these different species of Apogastropoda.

The increased numbers of introns which are present in hemocyanin genes of both distinctly-related species of Apogastropoda may be one of a multitude of molecular adaptations that enabled the enormous radiation as well as the great biodiversity of Caenogastropoda and Heterobranchia. Thus, our intent was to reconstruct exon–intron architectures of hemocyanins of additional Tectipleura lineages to investigate if increased numbers of introns, as Peña & Adema [22] have discovered in *BgHcl* genes, are also present in full-length hemocyanins of Tectipleura. This should provide insight into any correlation between adaptation to altered living environments and different gene structures of hemocyanins within this clade of gastropods.

## Results/discussion

We accomplished the full reconstruction of exon–intron architectures of ten hemocyanin coding sequences of four different Tectipleura species (*Aplysia californica, Lymnaea stagnalis, Cornu aspersum* and *Helix pomatia*) as well as three hemocyanin coding sequences of two Octopodoidea species (*Octopus bimaculoides*, *Octopus vulgaris*). The publicly available coding sequences of hemocyanins have been updated with annotations of exons under their accession numbers (see Methods). Genomic sequences used to generate gene architectures of hemocyanins of *C. aspersum* and *H. pomatia* were obtained and subsequently assembled, whereas for all other species we used publicly available genomic data (see Methods).

### Gene structures of *Tectipleura hemocyanins*: a multitude of conserved introns

**Fig. 3:**
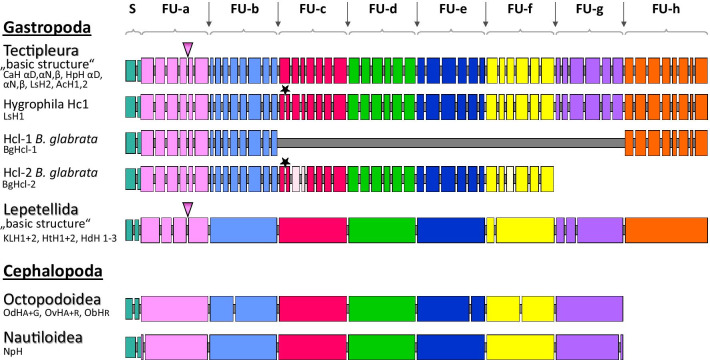
Gene structures of molluscan hemocyanins. The comparison of exon–intron architectures illustrates the conspicuously larger number of introns in hemocyanin genes of Tectipleura, compared to Lepetellida and Cephalopoda. Big boxes represent exons, small grey ones between them, introns. Positions of plotted splice sites correlate with their position relative to the coding sequence of the FUs. Widths of small boxes are not relative to intron length. Exons of signal peptides (S) and of different FUs are colored differently. The additional Hygrophila-specific intron (only in one of their hemocyanin genes, Hc1) can be seen in FU-c (star) and represents the only difference to the basic structure of Tectipleura hemocyanins. It is also present in *BgHcl*-2. Linker introns are conserved in their position between all FUs of hemocyanins of all species and are marked with grey arrows on top. Pink arrowheads show the only internal introns which are located at the identical position within Tectipleura and Lepetellida. For *BgHcl*-2 three internal introns are not fully proven by our analysis (brighter colored in FU-c and Fu-f). Gastropod gene structures are depicted for hemocyanins of Tectipleura (*CaH*: *Cornu aspersum*; *HpH*: *Helix pomatia*; *LsH*: *Lymnaea stagnalis*; *AcH*: *Aplysia californica*; *BgHcl*: *Biomphalaria glabrata*) and Lepetellida (*KLH*: *Megathura crenulata* (Keyhole limpet); *HtH*: *Haliotis tuberculata*; *HdH*: *Haliotis diversicolor*); for Cephalopoda hemocyanins of Octopodoidea (*OdH*: *Enteroctopus dofleini*; *OvH*: *Octopus vulgaris*; *ObH*: *Octopus bimaculoides*) and of Nautiloidea (*NpH*: *Nautilus pompilius*). Cephalopod hemocyanins differ from the basic molluscan hemocyanin structure by lacking FU-h

The gene structures of all analyzed Tectipleura hemocyanins are very similar (Fig. 3). With the exception of one hemocyanin gene from *Lymnaea stagnalis* (*LsH1*) that includes one additional intron, all full-length Tectipleura hemocyanin genes contain 53 introns within the coding sequence. In addition to the number of introns, the intron positions with respect to the coding sequences and the phases of these introns are identical. We assume that this gene architecture is primordial for Tectipleura hemocyanins because we have found it in the two sister groups Euopisthobranchia and Panpulmonata which together form the clade of Tectipleura (listed species in Fig. 1a)). Corresponding to the molecular clock of Zapata et al*.* [1] and Kano et al*.* [9], this implies that this architecture of hemocyanin genes of Tectipleura arose at least 230 ± 50 million years ago (mya).

Figure 3 compares the distribution of introns among the different hemocyanin FUs of Tectipleura and of other molluscs. As in all genes of molluscan hemocyanins, linker introns are located between all functional units (grey arrows in Fig. 3). However, the number of internal introns within Tectipleura hemocyanin genes is strikingly higher than in any published gene architecture of a molluscan hemocyanin so far: For Tectipleura hemocyanins we identified 46 internal introns (in *LsH1* even 47) in addition to the seven conserved linker introns, whereas there are three (*Nautilus pompilius*) and five (Octopodoidea) in Cephalopoda and eight internal introns in Lepetellida (Vetigastropoda) [16–18, 20]. The number of internal introns of Tectipleura hemocyanins is greater than that found in hemocyanin genes of *Pomacea canaliculata*, which comprise 21 to 26 internal introns per gene according to Chiumiento et al*.* [21]. The *P. canaliculata* gene structures are intentionally omitted from Fig. 3 and the following analyses because we detected inconsistencies in their assembly and annotation (e.g. two out of four genes are represented in the wrong orientation and some splice sites were not or incorrectly identified). However, we discuss these results because they unambiguously report a higher number of introns in hemocyanin genes of *P. canaliculata* compared to those of Cephalopoda or Vetigastropoda.

The positions of internal introns are distributed along the complete coding sequences of Tectipleura hemocyanin genes and are identical between different Tectipleura species (except for one additional intron in the *LsH1* gene of *L. stagnalis*). Exon–intron architectures of the individual FUs of Tectipleura hemocyanins, on the other hand, differ from each other. Each FU-coding sequence contains six to eight exons varying in size between 97 and 309 nucleotides with a median number of 189. As in other molluscan hemocyanins, internal introns of all three phases are present. The general phase distribution within Tectipleura hemocyanins is 52% of phase 0, 24% of phase 1 and 24% of phase 2. These results match the overall tendency that phase 0 is the most frequent intron phase among different genes of various species [24, 25].

### The additional intron in the *LsH1* gene is Hygrophila-specific

*LsH1* contains one additional intron in FU-c which has not been detected in the gene structures of any other Tectipleura other than Hygrophila (star in Fig. 3). Therefore, we confirmed the assembled sequence independently via PCR. We also uncovered the presence of this additional intron in one hemocyanin gene of *Radix balthica*, another species of Hygrophila. We searched for this specific nucleotide sequence section within genomic NGS data of *R. balthica* and found two corresponding hemocyanin sequences: one containing an additional intron as per *LsH1* and one without an intron at this position – like in *LsH2* and other Tectipleura hemocyanins. The hemocyanin genes to which those sequences belong to were named *RbH1* and *RbH2*, respectively. This additional intron is also present in *BgHcl-2*, as revealed by our analysis of this hemocyanin-like gene of *B. glabrata* (see below). Therefore, it can be considered as a Hygrophila-specific intron which evolved after the radiation event of this lineage from a Tectipleura ancestor.

### Hemocyanin-like proteins of *Biomphalaria glabrata* and full-length hemocyanins of other Tectipleura share the same gene structure

Previous to this study, only the exon–intron architectures of two hemocyanin-like protein genes of *Biomphalaria glabrata* (*BgHcl*-1, *BgHcl*-2) have been published for species of the clade of Tectipleura [22]. *Biomphalaria glabrata* belongs to Planorbidae, a family of Hygrophila which represents an exception within the clade of Tectipleura. Instead of hemocyanin, *B. glabrata* uses a hemoglobin as oxygen transporter which most probably evolved exclusively within this gastropod family [23, 26]. Thus, the hemocyanin isoforms of these species are probably not essential any more for oxygen transport but may have been repurposed to novel functions (suggestions for further functions *cf*. [22]). Furthermore, the detected proteins contained only three and six functional units, respectively, instead of eight FUs which is typical for other gastropod hemocyanins [22, 23].

The comparison of exon–intron structures of Tectipleura hemocyanin genes with those of hemocyanin-like protein genes of *B. glabrata* (Fig. 3) shows that all splice sites of the *BgHcl*-1 gene are located at the same position in the corresponding FUs (a, b and h) of the analyzed Tectipleura hemocyanin genes [22]. Through our reanalysis of the draft *B. glabrata* genome assembly, which was used by Peña and Adema to determine the coding sequence of the *BgHcl*-2 gene [22], we identified sequences that were defined as introns but are characteristic of hemocyanin coding sequences. Therefore, we assume that these are genuine exons and thus we suggest an expanded coding sequence and a novel gene structure. The revised exon–intron architecture of *BgHcl*-2 is highly similar to the gene structure of other Tectipleura hemocyanins (Fig. 3).

Differences of this exon–intron structure to those of full-length hemocyanins of Tectipleura are only present in sequence sections that were poorly resolved within the published genome of *B. glabrata*: In FU-c and in FU-f we have found sequence motifs which are usually conserved in hemocyanins but could not be detected in the *BgHcl*-2 gene. Also, the number of bases was smaller than characteristic for those hemocyanin functional units. Within FU-c the undetected parts correspond to two Tectipleura exons (exon 3 + 4). The section of the genome assembly in which those exons would have been expected includes ambiguous nucleotides (NNN’s). For FU-f the 3′ section of the third exon could not be determined due to stretches of NNN’s. On the basis of this draft genome, it is currently impossible to determine whether those exons are missing in *BgHcl*-2 or if they are just not properly assembled. In Fig. 3 these exons are included but are highlighted with brighter colors. Additionally, two of the newly identified exons were found in a reverse orientation within the draft genome (FU-b exon 4 and FU-f exon 1). Since they correspond to nucleotide sequences and exon–intron structures of other Tectipleura hemocyanins, we propose that they constitute exons of the *BgHcl*-2 gene. Incorrectly oriented exons are common errors in automated computational draft genome assemblies. Including these hypothetical exons in a predicted cDNA sequence, the gene architecture of *BgHcl*-2 has the same exon–intron structure of FU-a to FU-f of the *LsH1* gene in terms of number, positions and phases of introns (including the additional intron in FU-c which has been found exclusively in *LsH1* and *RbH1* genes; stars in Fig. 3).

This strict conservation of gene structures is particularly interesting, since the amino acid sequences of both *BgHcl* proteins differ significantly in sequence motifs that are otherwise conserved through all analyzed molluscan hemocyanins including those of Tectipleura, Vetigastropoda and Cephalopoda (see differences highlighted in the alignment in Additional file 2). The exon–intron structures of *BgHcl* genes, which remained consistent despite all these deviations from characteristic sequence features of other hemocyanin genes, highlights the strong conservation of introns within Tectipleura hemocyanin genes. Additionally, the identical splice site positions corroborate the hypothesis of Peña and Adema that those genes are descendants of the hemocyanin gene family. This suggests that the large number of introns should not be considered as a novel gain within hemocyanin-like genes and is, therefore, probably not linked to the evolution of novel functions of hemocyanin-like proteins in Planorbidae as suggested after their first discovery [22].

### Accumulation of introns in Tectipleura is hemocyanin gene-specific

To exclude that intron accumulation is a general phenomenon in Tectipleura genomes, we compared average numbers of introns as well as exon sizes of over 15,000 genes of two Tectipleura species, specifically *Aplysia californica* (GCF_000002075.1) and *Radix auricularia* [27]), with those of two Octopoda species, namely *Octopus bimaculoides* (GCF_001194135.1) and *O. vulgaris* (GCF_006345805.1). We found no general increase in the number of introns within Tectipleura genomes (Additional file 3a). Additionally, we compared exon counts of orthologous genes of *A. californica* and *O. bimaculoides* as well as *A. californica* and *O. vulgaris* (results shown in Additional file 3b). These exon count comparisons must be considered carefully because the underlying data encompass all data published in NCBI and were thus produced by different studies using different strategies of sequencing and data processing. Hence, comparability of these data is limited (cf. [28]). However, the results exhibit trends of gene architecture evolution which do not comprise extensive intron gains or losses between orthologs of *A. californica* and the two *Octopus* species. While the differences in exon counts of hemocyanin genes between these species is 35 (only including FU-a – Fu-g of *AcH* for comparability), we have only identified 7 and 6 orthologous genes that vary in exon count in more than 25 introns between *A. californica* and the two *Octopus* species, respectively (shown in table in Additional file 3b). 50% of the numbers of differences in exon counts encompass only up to one intron change and 80% up to four (*O. bimaculoides*) and up to six intron changes (*O. vulgaris*), respectively. Although this is only an approximate trend based on a preliminary analysis, widely varying numbers of introns between orthologous genes of Octopoda and Tectipleura seem to be characteristic for only few genes and do not represent a general phenomenon within these genomes. Apparently, the extensive accumulation of introns we observe in hemocyanins of Tectipleura is gene-specific. The strong lineage-specific conservation of these introns across disparate molluscan clades, on the other hand, argues against a random variation of internal introns but rather suggests a significant selection pressure acting on this gene structures. We therefore performed more in-depth analyses on this aspect of these genes.

## Highly conserved linker introns

Previous studies on molluscan hemocyanin gene structures revealed strong conservation of the so-called linker introns of hemocyanins (see above and [16]). As shown in Fig. 3, they are located within the linker peptide coding regions between all functional units of hemocyanins throughout different molluscan classes, including Tectipleura (grey arrows in Fig. 3). They are without exception phase 1 introns (located after the first nucleotide of a codon). Since they are shared by all modern molluscan clades, they must have evolved prior to the radiation of molluscs into different classes. Lieb et al*.* [16] assumed that this occurred during duplications of FUs through which the eight-FU hemocyanin molecule arose from an ancestral mono-FU. Despite the increasing amount of molluscan hemocyanin sequence data during the last decades, the chronological order of these duplication events and the exact evolutionary origin and function of linker introns remains unresolved.

Although linker introns of hemocyanin genes and their positions are conserved through all molluscan clades, we did not find any indications of concerted evolution, transposition or conserved regulatory elements by comparing their nucleotide sequences. Comparative studies of exon–intron structures of human genes identified high positional conservation as typical characteristics of introns carrying important functions [29]. Accordingly, these strictly conserved linker introns in molluscan hemocyanin genes might be functional, even though they vary in their sequences. Altenhein et al*.* [17] suggested, for example, that they could play a role for correct transcription of these giant polypeptides. However, since the nucleotide sequences of a multitude of these linker introns are not fully assembled yet, a final conclusion on their functional significance is still pending.

### Internal introns

The similarity of hemocyanin gene architectures of Tectipleura suggests that their exon–intron structures arose in a common ancestor and stayed almost the same for > 230 ± 50 mya (according to molecular clocks of [1] and [9]). Considering that linker introns of hemocyanin genes in Tectipleura match all other known hemocyanin genes of molluscs in terms of their positions and phases, we concentrated on the internal introns which lie within the functional units of hemocyanin genes. Splice sites of internal introns of molluscan hemocyanins have not been found to be highly conserved in previous studies. Lieb et al*.* [16] showed that gene structures of hemocyanins in *Enteroctopus dofleini* (Cephalopoda, Octopodoidea) and in *Haliotis tuberculata* (Vetigastropoda) are totally different concerning numbers, positions, lengths and phases of their internal introns (Fig. 3). Bergman et al*.* [18] revealed that internal introns vary completely even between the cephalopod hemocyanins of *E. dofleini* and *Nautilus pompilius.* In contrast, the two different hemocyanin genes identified in *H. tuberculata* possess a highly similar genomic structure [17]. A comparable exon–intron architecture was also found for the three hemocyanin genes of *Haliotis diversicolor* [20] and the two of *Megathura crenulata* (*KLH1* and *KLH2*) [19]. Like Haliotoidea, *M. crenulata* belongs to Fissurelloidea, a group of Lepetellida (Vetigastropoda). Although the internal introns of hemocyanin genes through all these Lepetellida species vary in length and in their nucleotide sequences, they are located at the same position and in the same phase with respect to the coding sequence within all analyzed Lepetellida hemocyanins. This most probably reflects a common origin of evolution.

### Lineage-specific conservation of hemocyanin gene structures contrasts with high variability between major molluscan clades

To investigate whether internal introns are also conserved within the hemocyanin genes of closely related cephalopod groups, we analyzed hemocyanin genes from the published genomes of *Octopus vulgaris* and *Octopus bimaculoides*. We found that the derived exon–intron architectures of their hemocyanin genes correspond to those found in *Enteroctopus dofleini* concerning intron number, location and phase (Fig. 3). Due to their identities with hemocyanin genes of *E. dofleini* (shown in Additional file 4), we named the hemocyanin genes of *O. vulgaris OvH* Type-A (XM_029780310.1) and Type-R (XM_029796515.1) and the hemocyanin gene of *O. bimaculoides ObH* Type-R (XM_014934350.1; XM_014934481.1). As shown above, Tectipleura also displays a very conserved exon–intron structure of their hemocyanin genes. We therefore conclude that lineage-specific positional conservation of internal introns might be a common feature of molluscan hemocyanins.

A first comparison of the conserved exon–intron structure of Tectipleura hemocyanin genes with those splice sites of hemocyanin genes of *P. canaliculata*, which we were able to verify, showed that their exon–intron structures differ substantially from each other (e.g. 80% of the splice sites of the gene *PcHI* are absent within Tectipleura hemocyanins). Hence, we assume that the accumulations of internal introns in hemocyanin genes of *P. canaliculata* and in those of Tectipleura evolved independently from each other and thus arose most probably after the radiation of Apogastropoda into Caenogastropoda and Heterobranchia.

High conservation of intron positions as found for hemocyanin genes of Tectipleura is a frequently occurring phenomenon in orthologous genes of animals [30–32] but it is particularly striking for hemocyanin genes of Tectipleura, since they feature 46 conserved internal introns instead of three (*Nautilus pompilius*), four (Octopodoidea) or seven (Lepetellida) and all of them stayed at the exact same position for more than 200 million years. These results are furthermore surprising as they do not correspond with the hemocyanin gene structures of Caenogastropoda elucidated by Chiumiento et al*.* [21]. On one hand, their results indicate that accumulation of introns as we have detected in Tectipleura is a common feature of hemocyanin genes of Apogastropoda, but on the other hand, the four hemocyanin genes of *P. canaliculata* vary in number and positions of introns and do not show such a strong conservation as we have identified for Tectipleura.

Against this background, the strict conservation of the gene structure of Tectipleura hemocyanins appears not to be random. Instead, it may rather have been caused by an evolutionary pressure towards conservation of this exon–intron structure due to its supposed functional roles. Chorev et al*.* [29] described a correlation between, on one hand, high positional conservation of introns as well as low intron loss rates and, on the other, intronic functions. This might also be the case for internal introns of Tectipleura hemocyanin genes.

So far, we were not able to assemble all introns and to analyze them on sequence levels, because of large and highly repetitive sequence sections. We did, however, analyze 107 intron sequences of the two genes of *LsH1* and *LsH2* found in a soon to be released genome of *Lymnaea stagnalis.* Our sequence similarity searches did not reveal conserved sequence motifs within these intron sequences, neither by using the NCBI tool ‘BLAST’ [33], nor by applying our self-created databases of the *LsH* intron sequences.

However, it seems that introns rarely exhibit elevated degrees of conservation detectable with conventional similarity searches [29]. Small conserved motifs like cis-regulatory elements which often contribute to transcriptional regulation [33, 34] are hard to detect within long intron sequences such as those deciphered in hemocyanin genes of *L. stagnalis* (min: 118 bp; max: 4,657 bp; mean value 531). To enable the identification of short functional sequence sections within long mostly non-functional intronic sequences, a significantly larger dataset would be needed. Besides these difficulties, many intron functions do not depend on sequence motifs. Some functions rather depend on intron length [35] or on their positions along the mRNA [29, 36, 37].

In fact, the large number of internal introns with strictly conserved positions in hemocyanin genes of Tectipleura suggests that they may also exert functions that depend on their positions within the genes. The multitude of introns we have identified enables us to analyze the positions of internal introns in detail by drawing comparisons between internal introns of different lineages as well as of different FUs of hemocyanins of Tectipleura.

### Recent evolution of internal introns

Generally, there are two possible scenarios of intron evolution: Introns within a nuclear gene can be very ancient or can be added later during evolution of the gene (see review [38]). The clear segmentation into functional units concatenated by linker introns in different molluscan lineages (Fig. 3) implies that the basic structure of molluscan hemocyanin genes evolved prior to or during the radiation of molluscs into different clades and then remained conserved. In fact, the origin of linker introns has already been described as ancient [16]. While they remained conserved during speciation events, internal introns changed independently from each other in different FUs and different taxa. Consequently, this led to clade-specific patterns of gene structures that became fixed more recently during evolution in a lineage-specific manner, as seen in Octopodoidea, Lepetellida and Tectipleura (Fig. 3).

**Fig. 4:**
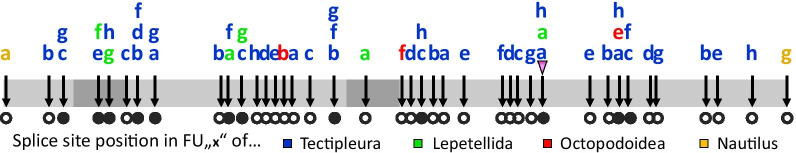
Distribution of splice sites in hemocyanin FUs. Splice site positions (arrows) are shown within the overlay of FU-coding sequences of hemocyanin genes (grey bar; darker parts: copper binding sites). They are spread over the sequence in clusters. Letters above indicate in which FU and in which molluscan group (color) they are located. About 48% of all internal introns are located at identical positions as introns in one or two other FUs (multiple splice site positions; marked with black dots). Positions that are unique for one splice site are marked with an open dot. The pink arrowhead shows the only internal introns which are located at the identical position within Tectipleura and Lepetellida (same position in ortholog FU (FU-a))

The overlay of hemocyanin functional units in Fig. 4 shows the positions of internal introns through all molluscan hemocyanin genes analyzed in the present study. More precisely, introns from all functional units of *N. pompilius*, Octopodoidea, Lepetellida and Tectipleura are marked together within one model FU-coding sequence. Due to ambiguities of the respective report, exon–intron architectures of hemocyanin genes of *P. canaliculata* [21] were not included in this figure.

Overall, the distribution of internal introns illustrated in the model FU in Fig. 4 exhibits a number of splice sites from different molluscan groups or different FUs which are located at equal positions with respect to the coding sequence (multiple splice site positions). About 48% of the splice sites occur at identical positions in two or three different FUs (of the same or of different hemocyanin genes, see color coding). Thereby, only one of them is observed at the same position within the same functional unit of hemocyanin genes from different groups (FU-a of Lepetellida and Tectipleura hemocyanins; pink arrowheads in Fig. 3 + 4). It is the only splice site that is located at the identical position concerning the whole coding sequence of the hemocyanin polypeptide within two different lineages. We call it an “ortholog intron” of Lepetellida and Tectipleura. Since both groups are gastropods, the introns can be real orthologs which arose before the gastropod radiation into the different lineages and remained conserved until today.

**Fig. 5:**
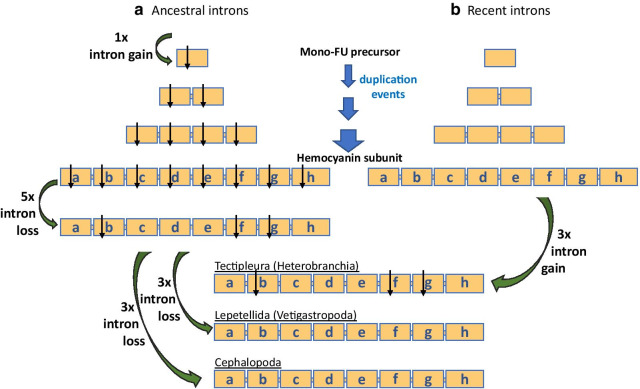
Scenarios of intron evolution in hemocyanin genes. Intron evolution is exemplified by evolution of introns at identical splice sites of FU-b, FU-f, FU-g (*cf.* Fig. 4). Shown are two scenarios: **a** The introns at the identical position are derived from an ancestral intron which evolved within the mono domain precursor. In this scenario the intron maintained at the identical position during the evolution of the eight-FU hemocyanin. It then stayed conserved within FU-b, FU-f and FU-g of Tectipleura hemocyanins until today, whereas it was lost within the remaining Tectipleura FUs and all FUs of Vetigastropoda and Cephalopoda. In scenario (**b**), the introns evolved more recently after the radiation events of Gastropoda into Vetigastropoda and Heterobranchia. The intron gains took place independently from each other at the identical position of FU-b, FU-f and FU-g of Tectipleura. Scenario **a** includes at least 12 intron gain/loss events, scenario **b** only three intron gains. Thus, the more recent intron gains are the more parsimonious scenario

All other multiple occurring splice sites, however, are located within different functional units of hemocyanin genes of the same or of different molluscan lineages. If these splice sites were homologous as well, they must be “paralogous” to each other because they would have derived from an ancient intron due to tandem duplications of FUs: Regarding the positions within different FUs as paralogs they must have been gained in the mono-FU hemocyanin ancestor and then duplicated during the evolution of the eight-FU protein (example represented in Fig. 5). Since this would implicate that these splice sites were lost within much more FUs than they would have stayed conserved, we do not support this hypothesis as origin. In contrast to that, independent intron gains within different FUs during more recent evolutionary times represent a much more parsimonious scenario (Fig. 5).

Additionally, 52% of the detected hemocyanin splice sites occur exclusively in one functional unit of one lineage-specific hemocyanin gene structure (unique splice sites). This can also be explained best by independent intron gains. Thus, we propose independent and recent gains for most internal introns of molluscan hemocyanin genes. This is further corroborated by a comparison of molluscan hemocyanin genes with splice sites of different Type-III copper protein genes (data not shown) that share the same origin with hemocyanins [39]. None of the 26 analyzed genes indicate ancestral intron evolution.

### Identical intron positions due to conserved proto-splice sites?

**Fig. 6:**
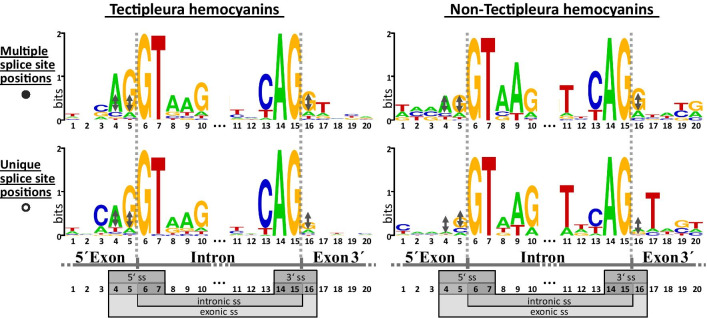
Nucleotide distribution at splice sites (ss) of internal introns. Five nucleotides up- and downstream of each splice site are shown in weblogos. The height of the nucleotide letters indicates the frequency of each nucleic acid at the respective position. Sequence logos at the left depict splice sites of hemocyanins of Tectipleura; those at the right of Lepetellida, Octopodoidea and *Nautilus pompilius* (taken together as non-Tectipleura hemocyanins). Internal introns are further distinguished in introns at multiple (top, black dot, *cf.* Fig. 4) and introns at unique splice site positions (bottom, open dot, *cf.* Fig. 4). Multiple splice site positions are defined as positions along the cDNA where at least one different intron is located (*cf.* Fig. 4). This can be located in a different FU of the same gene structure type or in any FU of a different gene structure type. At unique splice site positions, no introns of other FUs/hemocyanins are located at the identical position with respect to the coding sequence (*cf.* Fig. 4). The intronic splice site GT-AG is highly conserved throughout all hemocyanins. Additionally, weblogos display the existence of the (exonic) proto-splice site AG|G. For better comparison of the frequency of AG|G in the four different weblogos, black double arrows of the same height are given. While all proto-splice site nucleotides of multiple splice site positions of Tectipleura and non-Tectipleura (upper ones) are higher than those reference arrows, some of the unique splice site positions are much smaller

Our analysis revealed highly conserved sequence motifs of exon–intron boundaries. The weblogos in Fig. 6 show the nucleotide distribution of exon and intron boundaries of internal splice sites (five nucleotides up- and downstream of each splice site). 99.3% of the intron sequences we analyzed start with the dinucleotide GT and end with AG. Therewith, they fulfill the Chambon’s / GT-AG rule [40, 41]. This is in accordance with other studies that revealed GT-AG boundaries for over 98.5% of introns [42, 43]. As exceptions from this rule, the splice sites GC-AG were found twice in Hygrophila and Stylommatophora (Fig. 6). They represent 0.7% of all Tectipleura splice sites. This also fits to the results of Burset et al*.* [43] who described GC-AG as the second most frequent splice site covering 0.6% of all intron boundaries within their study.

In contrast to intronic splice sites, exonic splice sites vary greatly. However, an accumulation of proto-splice sites at exon boundaries can be detected within the consensus sequences represented by the weblogos (CAG at 3′ and GT at 5′ ends of the exons, Fig. 6). This tendency is stronger within Tectipleura hemocyanin genes than in those of Lepetellida, Octopodoidea and *N. pompilius* (taken together in Fig. 6). For Tectipleura as well as for the other three molluscan groups a distinction is made between splice site positions that occur just once (unique splice site positions) or in at least two different FU-coding sequences of the analyzed hemocyanin genes (multiple splice site positions; *cf.* open vs. filled dots in Fig. 4 + 6). Multiple splice site positions of both groups show an increased occurrence of AG|G proto-splice sites. In contrast, the presence of proto-splice sites at unique splice site positions is significantly less frequent in Tectipleura hemocyanins and not at all increased for non-Tectipleura hemocyanins.

These results display a larger frequency of proto-splice sites at positions that harbor introns more often. This is consistent with previous studies which showed that splice sites often include canonic nucleotide patterns like (C/A)AG|Gt [44–46]. Thereby, AG|G is significantly more frequent than any other exon boundary [47–50]. This is in accordance with our findings (Fig. 6). Although this splice site cannot be critical for correct splicing due to its relatively low conservation, a positive selection in its favor can be assumed. It has been hypothesized that canonical proto-splice sites might, for example, improve the efficiency of splicing while they simultaneously offer a wide flexibility for protein coding and the evolution of the coding sequence, if the respective introns are in phase 0 [50]. It is still unclear if the accumulation of specific splice site motifs arose due to more frequent intron gains or due to a higher positional conservation. Both reasons, however, might have led to the increased occurrence of multiple splice sites at such positions during the evolution of hemocyanin genes.

Thus, it can be assumed that internal introns which are located at identical positions in multiple FUs might have been gained convergently. This is in accordance with the maximum parsimony scenario of more recently and independent gains of internal introns described above. A correlation between highly conserved proto-splice sites and more frequent convergent intron gains has already been discovered by the studies on elongation factor genes of Hymenoptera by Klopfstein and Ronquist [51].

### Splice site positions of internal introns do not correlate with module boundaries

The distribution of splice sites along the sequence of a model FU shows an accumulation of introns in clusters, whereas other sequence sections are intron-less (Fig. 4). Alignments of amino acid sequences showed that splice sites lie in highly conserved as well as in rarely conserved regions (alignments in Additional files 5 and 6). Additionally, our analyses revealed that they are located within sequences coding for alpha helices and beta sheets as well as for random coils (videos in Additional file 7). We could not identify specific positional conservation of internal introns that correspond to hypothetical module boundaries in ancient proteins as described by de Souza et al*.* [52] and, for example, as for linker introns which lie between the single FUs [16]. Accordingly, the clusters of introns cannot be explained by structural features nor by high or low conservation patterns of the coding sequence.

These clusters, however, could result from the existence of linker introns which lead to a regular fragmentation of hemocyanins in similar composed FUs. The coexistence of regular size distribution of exons which we found within these FUs of Tectipleura hemocyanins (97 – 309 nucleotides) must consequently have led to an incorporation of introns at similar positions with respect to the coding sequences of the eight different but relatively equal constructed FU fragments. Thereby, the roughly regular subdivision of FUs by internal introns leads to clusters in the overlay of several FUs.

In spite of our analyses, we are not able to deduce any importance for positions of internal intron splice sites which might be crucial for their possible functions. They rather seem to be distributed in a roughly regular manner within the FUs with the exception of introns at proto-splice sites which most probably were gained several times convergently at those positions and remained there until today. Thus, it remains open why hemocyanin genes of Tectipleura comprise such a large number of introns in contrast to other molluscan hemocyanin genes.

### Evolutionary pressure, adaptive radiation and habitat shifts

The conservation of such an extensive number of introns throughout long periods of evolution within different molluscan lineages is especially remarkable for Tectipleura, since they cover a distinctly larger number of introns. Although this increases the probability of intron loss or sliding, we did find none of them in the hemocyanin genes of Tectipleura species we analyzed in this study. As already described, Hygrophila comprise an additional internal intron within FU-c which is only conserved within this clade. However, these hemocyanin genes also comprise all primordial Tectipleura introns.

Furthermore, Tectipleura, whose radiation can be traced back to ~ 230 ± 50 mya (according to molecular clocks of [1] and [9]) represent a conspicuously larger clade (~ 27,100 species) compared to Lepetellida (~ 950 species) or Octopodoidea (~ 240 species) which also possess conserved exon–intron structures in their hemocyanin genes (for numbers see [2]). This indicates that Tectipleura were subjected to a stronger degree of radiation and an increased evolutionary rate. Such preconditions are normally expected to also increase the probability of changes on gene structures unless no evolutionary pressure would have acted upon these introns.

In addition to a multitude of speciation events, Tectipleura have undergone a variety of habitat shifts during their evolution which led to radical changes of their living conditions. In contrast to Vetigastropoda, which include very early branching and exclusively marine living gastropods, Tectipleura comprise very diverse snails and slugs living in marine (e.g. *A. californica*), freshwater (e.g. *L. stagnalis*), terrestrial (e.g. *H. pomatia*) as well as intermediate environments (Fig. 2). Consequently, they must have undergone several adaptations such as an increased capacity for osmoregulation, tolerance to fluctuating temperatures and varying degrees of water availability, as well as the capacity to breath air [10]. This resulted in a range of diverse and complex species. The respiratory systems of Tectipleura, for example, were modified in many ways through the evolution of lungs and of pneumostomes [7, 10], or through duplications of hemocyanin genes that took place [11]. Regarding all these changes during the evolution of different Tectipleura lineages, the exact conservation of so many splice sites within these genes appears extraordinary, indicating a high degree of evolutionary pressure for their conservation. In the absence of evolutionary constraints, however, a more continuous alteration of exon–intron structures of hemocyanins would have been expected including less sudden extreme changes between different molluscan lineages followed by a long period of high conservation of special gene structures. For Tectipleura, for example, the gene structure including the multitude of internal introns evolved after the radiation of gastropods into their main groups (including Vetigastropoda and Heterobranchia) but within a common ancestor of Tectipleura. According to the molecular clock of Zapata et al. [1], this implies that this gene structure which varies in 45 introns from that of Vetigastropoda evolved between ~ 480 ± 50 mya and ~ 230 ± 50 mya and thus within 250 ± 100 million years, whereas it then stayed strongly conserved for at least ~ 230 ± 50 million years. These strongly contrasting evolutionary rates may have occurred from changed evolutionary pressures.

In spite of the many conserved internal intron positions, however, we could neither identify any conserved sequence motifs within introns nor within splice site positions that correlate with module boundaries indicating specific functions. Consequently, no particular evolutionary pressure can be recognized acting on the hemocyanin gene architecture, neither with respect to intron sequences nor to their positions within the respective hemocyanin genes. However, a selection pressure that led to the strong intron conservation within Tectipleura hemocyanin genes, at least, cannot be excluded. This selective pressure could have acted, for example, on the number of introns which may bring advantages due to different functions or regulatory capacities.

One function of introns which has been proposed by Bonnet et al. [53] is that they help to protect genomes of eukaryotes from ‘transcription-associated genetic instability’. The results of their study show that the recruitment of spliceosome onto the mRNA decrease R-loop formation during transcription which represent stable hybrids between mRNA and DNA that lead to single-stranded DNA. The aggregation of many R-loops which can result from high expression rates of a gene, however, can cause DNA damage. Thus, high degrees of introns in genes, which lead to spliceosome recruitments, can help to protect DNA stability. R-loop prevention due to introns has been shown to be conserved for yeast as well as for human [53]. Therefore, it might also play a role in molluscs and could lead to an evolutionary pressure lying on introns as it might be the case for hemocyanins of Tectipleura.

Against the background that hemocyanin genes of *P. canaliculata* also possess an increased number of introns which most probably evolved independently from those of Tectipleura, our results also suggest a connection between a high rate of introns in hemocyanin genes and an enormous diversity of species and habitats. The multitude of introns in hemocyanin genes of Apogastropoda were gained most probably after the radiation into Heterobranchia and Caenogastropoda independently within these clades. Thereby, they potentially could have offered an exceptionally great chance of regulation and based on this also an increased rate of adaptive radiation and speciation.

Previous studies have shown that introns in general can provide a variety of regulation possibilities like incorporation of noncoding RNA genes [54–56], controlling of mRNA transport [57] and enhancement of gene expression [58]. Eghbalnia et al. [59], for example, analyzed exon:intron expression to further understand the role of exon and intron counts and concluded that changed intron expression may be involved in regulation of physiological processes. Overall, splicing can have regulatory effects on all levels of gene expression and may help to adapt to new living conditions [60, 61]. Gotic et al*.* [62] identified temperature-mediated splicing efficiency of pre-mRNA as an important control mechanism of gene expression of β-globins. It might be a widespread post-transcriptional mechanism to regulate mRNA accumulation and protein biosynthesis due to splicing factors [63]. For example, temperature-dependent splicing controls flowering at distinct temperatures in plants [64] or helps *Drosophila* to adapt to different temperatures [65]. Referring to hemocyanins, the large number of internal introns may allow a graduated temperature-dependent splicing which might help to regulate gene expression of hemocyanins at different temperatures. Such regulatory mechanisms offer great adaptability which is particularly important for limnic and especially terrestrial gastropods because they underlie a much stronger fluctuation of temperatures and other environmental conditions than marine molluscs. Temperature, on the other hand, deeply influences the oxygen affinity of hemocyanins [3–6]. This illustrates the significant effects that habitat shifts can have on the respiratory system and reflects the necessity of adaptations of these oxygen transporters to new living conditions.

Intron positions that possibly correlate with transitions to terrestrial habitats have also been described for plants [66]. Our study now provides some indication that such a correlation may also exist for Tectipleura, and maybe for animals in general. To adapt to decreased oxygen affinities (which result from increased temperatures), hemocyanin expression might, for example, be upregulated to ensure the transport of a sufficient amount of oxygen.

Previous analyses of hemocyanin genes [11] have disclosed a number of gene duplications which took place multiple times independently in different Tectipleura lineages. This suggests that multiple hemocyanin genes, which can be expressed differentially, might help to adapt to varying ecological conditions. This hypothesis was corroborated by the results of Chiumiento et al*.* (2020) who identified four hemocyanin genes in the *P. canaliculata* which represent further examples of multiple hemocyanin gene duplications associated with adaptations to air breathing lifestyles. Differential expression of isoform genes is an essential mechanism to adapt to different environmental conditions and has already been shown for hemocyanin genes of *Sepia officinalis* (Cephalopoda) [67]. In connection with different hemocyanin isoforms that possibly exhibit different oxygen affinities [11], a temperature-dependent splicing would increase hemocyanin variability which not only could enhance gastropod adaptability but also biodiversity and radiation. Due to the increased chance of adaptive radiation, the extensive number of conserved introns in hemocyanin genes may have supported diversification and speciation within Caenogastropoda and Heterobranchia, which represent by far the largest and most diverse groups of gastropods.

## Conclusions

The hemocyanin genes of Tectipleura possess a significantly larger number of introns than any other analyzed molluscan hemocyanins. In this study we show that the majority of them most probably originated from intron gains which took place after the radiation into the major gastropod clades. Nevertheless, the exon–intron architectures of Tectipleura hemocyanin genes have been conserved for more than 200 million years concerning the number and positions of introns. The conservation of splice sites in these genes may be due to as yet unidentified functional roles. The increase in the number of introns in Tectipleura hemocyanin genes may have supported the frequent habitat shifts observed in this clade. For example, a multitude of introns could increase the possibilities for alternative gene regulation and help a species to adapt to new living conditions, such as strong temperature fluctuations. This potential genetic plasticity may have supported the transition to new environments (e.g. land and freshwater) and the radiation of the Tectipleura. Our findings outline another dimension of the importance and the fundamental functions of introns and suggest the need for additional research. Thereby, molluscan hemocyanins represent a good tool to further investigate the role of introns during habitat shifts because the link between changed habitats and the need of adaptations of respiratory systems, including hemocyanins, is obvious.

## Methods

### Animal sampling and DNA isolation

Adult snails of *Cornu aspersum* and *Helix pomatia* were obtained from a commercial dealer (Wiener Schneckenmanufaktur e.U., Vienna, Austria). Three individuals of the species *Lymnaea stagnalis* were collected from a pond in Mainz. Three individuals of each species were anesthetized on ice for 20 min and subsequently sacrificed by quickly cutting off the head to minimize pain. Hepatopancreatic tissue was isolated on an ice cooled aluminum plate. Tissue aliquots were stored in RNAlater™ (Invitrogen by Thermo Fisher Scientific, Waltham, MA, USA) at − 80 °C. Samples were homogenized with a Precellys® homogenizer (Bertin Instruments, Montigny-le-Bretonneux, France). Subsequently, DNA was isolated applying the DNeasy Plant Mini Kit (Qiagen, Hilden, Germany). DNA integrity was checked on a 0.8% agarose gel (Biozym Scientific GmbH, Hessisch Oldendorf, Germany) and quantified via Nanodrop (ThermoFisher).

### Compiling exon–intron structures of hemocyanin genes of *Cornu aspersum* and *Helix pomatia*

DNA samples from one adult snail of *C. aspersum* and *H. pomatia* were sent to StarSeq (Mainz, Germany) for NGS (Illumina Next Seq500) and library preparation. Bioinformatics were performed using Geneious 9.1.8 [68]. Sequencing adapters were removed and raw reads were quality trimmed. Processed genomic data were mapped to coding sequences of three known hemocyanins of *C. aspersum* (*CaH αD*: MH485355, *CaH αN*: MH485356, *CaH β*: MH485357) and *H. pomatia* (*HpH αD*: MH485358, *HpH αN*: MH485359, *HpH β*: MH485360). Sequence sections which were not covered by genomic NGS data or which were incongruous to cDNA sequences were used to separate nucleotide sequences in different parts which represent segments of different exons. Exon sequences were completed by repetitive mappings of genomic data to these sequence parts until non-cDNA sequences were assembled. At least ten base pairs of the 3′ and 5′ ends of each intron were assembled to assure that flanking sequences differ from those of neighboring cDNA sequences and therefore represent introns.

### Compiling exon–intron structures of hemocyanin genes of *Lymnaea stagnalis*

Sequences of the previously published coding sequences of two hemocyanins found in *L. stagnalis* (*LsH1*: MH485363; *LsH2*: MH485364) were searched within the draft genome of *L. stagnalis* [69] using BLAST. Genomic scaffolds which included hemocyanin isoforms were aligned to amino acid sequences using GeneWise [70] to derive the exon–intron architectures of *LsH1* and *LsH2*. Those gene structures were verified by mapping genomic raw data (SRA (NCBI): ERR1083352 to ERR1083359) to the deduced exon sequences. The *LsH1*-specific intron which was additional to those found in other Tectipleura hemocyanins (Fig. 3, star) was additionally confirmed via PCR (see below).

### Compiling exon–intron structures of hemocyanin genes of *Aplysia californica*

Large parts of the exon–intron architecture of *AcH1* have already been detected by Streit et al*.* [71] within the *Aplysia genome project* database Apl. Cal. 1.0. Undetected splice sites were determined via amplification and sanger sequencing. Now, we additionally verified this exon–intron structure by the version Apl. Cal. 2.0 of the *Aplysia genome project* database (released August 2006/ February 2009) and the UCSU Genome Browser [72]. Using the already published coding sequences (*AcH1*: BK010575 and *AcH2* BK010576), we furthermore determined the exon–intron structure of *AcH2*. This is a partial hemocyanin gene which has been identified in the genome, whereas no 3′ end (including a stop codon) was found. Also, no second hemocyanin could have been identified within the hemolymph of *Aplysia californica* [73]. Both exon–intron architectures have been fully verified with the help of genomic raw data (SRA (NCBI): SRX044044, SRX044049, SRX044050, SRX044058, SRX044081) as described for *LsH1* and *LsH2* (see above).

### Compiling exon–intron structures of hemocyanin genes of two Octopus species

Sequences of previously published predicted nucleotide sequences of hemocyanins found in *Octopus vulgaris* (*OvH*_*A*_: XM_029780310.1; *OvH*_*R*_: XM_029796515.1) and *Octopus bimaculoides* (*ObH*_*R*_: XM_014934350.1; XM_014934481.1) and corresponding scaffolds of genome assemblies (NC_043001.1; NC_043024.1; NW_014775317.1; NW_014779982.1) were used to deduce exon–intron architectures via GeneWise [70] as described for *L. stagnalis* (see above).

### Screening genomic NGS data of *Radix balthica* for Hygrophila-specific intron within FU-c

To verify whether the additional intron we found exclusively in *LsH1* is lineage-specific, we used genomic raw data of *Radix balthica* (SRA (NCBI): ERR2531849). We mapped the genomic NGS data to the additional exon as well as to the flanking exons of *LsH1* and to the corresponding sequence sections of *LsH2*. Afterwards, we analyzed whether bordering sequences represent hemocyanin-characteristic motifs which fit to cDNA sequences or if they constitute intronic parts.

### Sequence analysis of introns of hemocyanin genes of *Lymnaea stagnalis*

We extracted 107 intron sequences of a soon to be released genome of *L. stagnalis* and searched sequence similarities to published nucleotide sequences using the NCBI tool ‘BLAST’ [74]. Additionally, we compared all those introns with each other to check them for conserved sequence motifs. Thereby, we created a database of all *LsH* intron sequences and investigated them using BLAST via Geneious 9.1.8 [68].

### PCR confirmation of ambiguous hemocyanin sequence sections

For exon–intron borders which had a low assembly quality or which deviated from the Chambon’s / GT-AG rule (Breathnach et al., 1978; Jacob & Gallinaro, 1989) in hemocyanin genes of *H. pomatia* and *C. aspersum*, gene-specific primers (Additional file 8, Table S3A)) were designed (CLC main workbench, Version 6.9) and respective gene regions were confirmed via Long Distance (LD) PCR for *H. pomatia*. For hemocyanin genes of *L. stagnalis* the additional intron of FU-c which appears exclusively in *LsH1* was PCR confirmed, too.

Long Fragments were PCR-amplified applying the Platinum™ SuperFi™ Green PCR Master Mix (Invitrogen by Thermo Fisher Scientific, Waltham, MA, USA) whereas shorter fragments were generated using the Advantage® 2 Polymerase Mix (Takara Bio Europe, Saint-Germain-en-Laye, France) (for PCR parameters see Additional file 8: Table S4). PCR products were visualized on a 0.8% agarose gel (Biozym, Hessisch Oldendorf, Germany). PCR products were cleaned up directly using the PCR clean-up kit (Qiagen, Hilden, Germany) or gene-specific bands were cut out and purified with the QIAquick Gel Extraction kit (Qiagen, Hilden, Germany). If possible, clean gene-specific products were sequenced directly by Microsynth (Balgach, Switzerland) using the same primers for sequencing as applied for LD PCR (Additional file 8: Table S3 (A)). Otherwise, they were cloned using the TOPO™ XL-2 Complete PCR Cloning Kit (Invitrogen by Thermo Fisher Scientific, Waltham, MA, USA) for long fragments or the TOPO® TA Cloning® Kit (Invitrogen by Thermo Fisher Scientific, Waltham, MA, USA) for shorter fragments. Three plasmids of one or two individuals were purified using the QIAprep Spin Miniprep Kit (Qiagen, Hilden, Germany) and sequenced via Sanger sequencing by Microsynth (Balgach, Switzerland) (sequencing primers in Additional file 8, Table S3 (B)).

### Software used for sequence analysis

Alignments of amino acids (additional files 2, 5, 6) were compiled in MEGA 7 [75] using the muscle algorithm [76]. UCSF Chimera [77] was used to create illustrations of hemocyanins as well as the video that shows the overlay of splice site positions of hemocyanin FUs within the 3D reconstruction (additional file 7; PDB-ID: 1JS8). Sequence logos were generated by WebLogo Version 2.8 [78]. All sequences derived from LD PCR were analyzed with CLC main workbench (version 6.9).

### Analyzing orthologs of *Aplysia californica* and two *Octopus* species

To enable rough comparison of intron evolution of other genes, we analyzed the number of introns in ortholog genes of *Aplysia californica* and (i) *Octopus bimaculoides* and (ii) *O. vulgaris*. We used OrthoVenn2 [79] for orthologous gene comparisons of all entries of the protein database in NCBI for the three species (as of 25.09.2020). We compared polypeptide lengths and exon counts of the results, filtered them to obtain all orthologous genes which possess entries on exon count and do not differ in polypeptide length in more than 5%. Additionally, we filtered paralogous genes if they encompass the same exon count. Comparing the differences in exon counts between orthologs of *A. californica* and the two *Octopus* species, we have only included the exon count of FU-a – FU-g of the gene coding for *AcH* to enable comparability for hemocyanins, because hemocyanin genes of octopuses do not encompass FU-h.

## Supplementary Information

**Additional file 1: Figure S1.** Hemocyanin gene structures.

**Additional file 2: Figure S2.**Sequence comparison of hemocyanin-like proteins of Biomphalaria glabrata with hemocyanins of gastropods and cephalopods.

**Additional file 3. Table S1.** Identities of cephalopod hemocyanins.

**Additional file 4: Table S2.** Identities of cephalopod hemocyanins.

**Additional file 5: Figure S3.** Splice site comparison of full hemocyanins.

**Additional file 6: Figure S4.** Splice site comparison of functional units.

**Additional file 7.** Movie files. Distribution of splice sites positions in a 3D model of a hemocyanin FU.

**Additional file 8: Table S3.** Primer description.

## Data Availability

The gene structures generated during the current study are available in NCBI under the accession numbers of their nucleotide sequences: *HpH αD*: MH485358; *HpH αN*: MH485359; HpH β: MH485360; *CaH αD*: MH485355; *CaH αN*: MH485356; *CaH β*: MH485357; *LsH1*: MH485363; *LsH2*: MH485364; *AcH1*: BK010575; *AcH2*: BK010576. Furthermore, hemocyanin cDNA sequences and their annotations derived during this study as well as the generated genomic NGS data that cover exons and splice sites of hemocyanins of *Cornu aspersum* and *Helix pomatia* will be available in Dryad repository (10.5061/dryad.b2rbnzsdj). For more information on the intronic sequences of *Lymnaea stagnalis* please contact the authors of this study as long as the data are not available online.

## Abbreviations

FU: Functional unit
mya: Million years ago
NGS: Next Generation Sequencing
AcH: *Aplysia californica* Hemocyanin
BgHcl: *Biomphalaria glabrata* Hemocyanin-like
CaH: *Cornu aspersum* Hemocyanin
HdH: *Haliotis diversicolor* Hemocyanin
HpH: *Helix pomatia* Hemocyanin
HtH: *Haliotis tuberculata* Hemocyanin
KLH: Keyhole Limpet hemocyanin / *Megathura crenulata* hemocyanin
LsH: *Lymnaea stagnalis* Hemocyanin
NpH: *Nautilus pompilius* Hemocyanin
ObH: *Octopus bimaculoides* Hemocyanin
OdH: *Enteroctopus dofleini* Hemocyanin
OvH: *Octopus vulgaris* Hemocyanin
PcH: *Pomacea canaliculata* Hemocyanin
RbH: *Radix balthica* Hemocyanin

## References

[1] Zapata F, Wilson NG, Howison M, Andrade SCS, Jörger KM, Schrödl M (2014). Phylogenomic analyses of deep gastropod relationships reject Orthogastropoda. Proc Biol Sci.

[2] Flanders Marine Institute. MolluscaBase. 2020–04–17. http://www.molluscabase.org/aphia.php?p=browser. Accessed 17 April 2020.

[3] Brix O, Bårdgard A, Cau A, Colosimo A, Condò SG, Giardina B (1989). Oxygen-binding properties of cephalopod blood with special reference to environmental temperatures and ecological distribution. J Exp Zool.

[4] Brix O, Colosimo A, Giardina B (1995). Temperature dependence of oxygen binding to cephalopod haemocyanins: Ecological implications. Marine and Freshwater Behaviour and Physiology.

[5] Mangum CP. Gas Transport in the Blood. In: Gilbert DL, Adelman WJ, Arnold JM, editors. Squid as Experimental Animals. Boston, MA: Springer US; 1990. p. 443–468. doi:10.1007/978-1-4899-2489-6_2010.

[6] Burnett LE, Scholnick DA, Mangum CP (1988). Temperature Sensitivity of Molluscan and Arthropod Hemocyanins. The Biological Bulletin.

[7] Jörger KM, Stöger I, Kano Y, Fukuda H, Knebelsberger T, Schrödl M (2010). On the origin of Acochlidia and other enigmatic euthyneuran gastropods, with implications for the systematics of Heterobranchia. BMC Evol Biol.

[8] Schrödl M (2014). Opinion: Time to say "Bye-bye Pulmonata"?. SPIXIANA.

[9] Kano Y, Brenzinger B, Nützel A, Wilson NG, Schrödl M (2016). Ringiculid bubble snails recovered as the sister group to sea slugs (Nudipleura). Sci Rep.

[10] Mordan P, Wade C. Heterobranchia II: The Pulmonata. In: Ponder W, editor. Phylogeny and Evolution of the Mollusca: University of California Press; 2008. p. 409–426. doi:10.1525/california/9780520250925.003.0015.

[11] Schäfer GG, Pedrini-Martha V, Schnegg R, Dallinger R, Jackson DJ, Lieb B (2018). Hemocyanin genes as indicators of habitat shifts in Panpulmonata?. Mol Phylogenet Evol.

[12] Miller KI, Cuff ME, Lang WF, Varga-Weisz P, Field KG, van Holde KE (1998). Sequence of the Octopus dofleini hemocyanin subunit: structural and evolutionary implications. J Mol Biol.

[13] Boteva R, Severov S, Genov N, Beltramini M, Filipii B, Ricchelli F (1991). Biochemical and functional characterization of Rapana thomasiana hemocyanin. Comp Biochem Physiol B.

[14] Senozan N, Landrum J, Bonaventura J, Bonaventura C. Hemocyanin of the giant keyhole limpet, Megathura crenulata. In: Lamy J, Lamy J, editors. Invertebrate oxygen-binding proteins: Structure, active site, and function : Proceedings of a workshop sponsored by the European Molecular Biology Organization, held in Tours, France, August 20–24 1979. New York: Dekker; 1981. p. 703–717.

[15] Markl J (2013). Evolution of molluscan hemocyanin structures. Biochim Biophys Acta.

[16] Lieb B, Altenhein B, Markl J, Vincent A, van Olden E, van Holde KE, Miller KI (2001). Structures of two molluscan hemocyanin genes: significance for gene evolution. Proc Natl Acad Sci U S A.

[17] Altenhein B, Markl J, Lieb B (2002). Gene structure and hemocyanin isoform HtH2 from the mollusc Haliotis tuberculata indicate early and late intron hot spots. Gene.

[18] Bergmann S, Lieb B, Ruth P, Markl J (2006). The hemocyanin from a living fossil, the cephalopod *Nautilus pompilius*: protein structure, gene organization, and evolution. J Mol Evol.

[19] Altenhein B, Lieb B, Awenius C, Markl J. Gene Structure of Gastropod Hemocyanin. Zoology Suppl. III. Proceedings of the 93th Annual Meeting Bonn, Germany; 2000.

[20] Yao T, Zhao M-M, He J, Han T, Peng W, Zhang H (2019). Gene expression and phenoloxidase activities of hemocyanin isoforms in response to pathogen infections in abalone Haliotis diversicolor. Int J Biol Macromol.

[21] Chiumiento IR, Ituarte S, Sun J, Qiu JW, Heras H, Dreon MS (2020). Hemocyanin of the caenogastropod Pomacea canaliculata exhibits evolutionary differences among gastropod clades. PLoS ONE.

[22] Peña JJ, Adema CM (2016). The Planorbid Snail Biomphalaria glabrata Expresses a Hemocyanin-Like Sequence in the Albumen Gland. PLoS ONE.

[23] Lieb B, Dimitrova K, Kang H-S, Braun S, Gebauer W, Martin A (2006). Red blood with blue-blood ancestry: Intriguing structure of a snail hemoglobin. Proc Natl Acad Sci.

[24] Long M, Deutsch M (1999). Association of intron phases with conservation at splice site sequences and evolution of spliceosomal introns. Mol Biol Evol.

[25] Fedorov A, Suboch G, Bujakov M, Fedorova L (1992). Analysis of nonuniformity in intron phase distribution. Nucleic Acids Res.

[26] Figuerdo EA, Gomez MV, Heneine IF, Santos IO, Hargreaves FB (1973). Isolation and physicochemical properties of the hemoglobin of biomphalaria glabrata (Mollusca, Planorbidae). Comp Biochem Physiol B.

[27] Schell T, Feldmeyer B, Schmidt H, Greshake B, Tills O, Truebano M (2017). An annotated draft genome for Radix auricularia (Gastropoda, Mollusca). Genome Biol Evol.

[28] Mardis ER (2016). The challenges of big data. Dis Models Mech.

[29] Chorev M, Joseph Bekker A, Goldberger J, Carmel L (2017). Identification of introns harboring functional sequence elements through positional conservation. Sci Rep.

[30] Shah DM, Hightower RC, Meagher RB (1983). Genes encoding actin in higher plants: intron positions are highly conserved but the coding sequences are not. J Mol Appl Genet.

[31] Rogozin IB, Wolf YI, Sorokin AV, Mirkin BG, Koonin EV (2003). Remarkable Interkingdom Conservation of Intron Positions and Massive, Lineage-Specific Intron Loss and Gain in Eukaryotic Evolution. Curr Biol.

[32] Chorev M, Carmel L (2013). Computational identification of functional introns: high positional conservation of introns that harbor RNA genes. Nucleic Acids Res.

[33] Das D, Clark TA, Schweitzer A, Yamamoto M, Marr H, Arribere J (2007). A correlation with exon expression approach to identify cis-regulatory elements for tissue-specific alternative splicing. Nucleic Acids Res.

[34] Narlikar L, Ovcharenko I (2009). Identifying regulatory elements in eukaryotic genomes. Brief Funct Genomic Proteomic.

[35] Thummel CS, Burtis KC, Hogness DS (1990). Spatial and temporal patterns of E74 transcription during Drosophila development. Cell.

[36] Cheng J, Belgrader P, Zhou X, Maquat LE (1994). Introns are cis effectors of the nonsense-codon-mediated reduction in nuclear mRNA abundance. Mol Cell Biol.

[37] Nagy E, Maquat LE (1998). A rule for termination-codon position within intron-containing genes: when nonsense affects RNA abundance. Trends Biochem Sci.

[38] Rogozin IB, Carmel L, Csuros M, Koonin EV (2012). Origin and evolution of spliceosomal introns. Biol Direct.

[39] Decker H, Terwilliger N (2000). Cops and robbers: putative evolution of copper oxygen-binding proteins. J Exp Biol.

[40] Breathnach R, Benoist C, O'Hare K, Gannon F, Chambon P (1978). Ovalbumin gene: evidence for a leader sequence in mRNA and DNA sequences at the exon-intron boundaries. Proc Natl Acad Sci USA.

[41] Jacob M, Gallinaro H (1989). The 5' splice site: phylogenetic evolution and variable geometry of association with U1RNA. Nucleic Acids Res.

[42] Mount SM (1982). A catalogue of splice junction sequences. Nucleic Acids Res.

[43] Burset M, Seledtsov IA, Solovyev VV (2000). Analysis of canonical and non-canonical splice sites in mammalian genomes. Nucleic Acids Res.

[44] Dibb NJ, Newman AJ (1989). Evidence that introns arose at proto-splice sites. EMBO J.

[45] Rogers JH (1989). How were introns inserted into nuclear genes?. Trends Genet.

[46] Lehmann J, Eisenhardt C, Stadler PF, Krauss V (2010). Some novel intron positions in conserved Drosophila genes are caused by intron sliding or tandem duplication. BMC Evol Biol.

[47] Sverdlov AV, Rogozin IB, Babenko VN, Koonin EV (2004). Reconstruction of ancestral protosplice sites. Curr Biol.

[48] Rogozin IB, Sverdlov AV, Babenko VN, Koonin EV (2005). Analysis of evolution of exon-intron structure of eukaryotic genes. Brief Bioinformatics.

[49] Sverdlov AV, Rogozin IB, Babenko VN, Koonin EV (2005). Conservation versus parallel gains in intron evolution. Nucleic Acids Res.

[50] Ruvinsky A, Ward W (2007). Intron Framing Exonic Nucleotides: A Compromise Between Protein Coding and Splicing Constraints. TOEVOLJ.

[51] Klopfstein S, Ronquist F (2013). Convergent intron gains in hymenopteran elongation factor-1α. Mol Phylogenet Evol.

[52] de Souza SJ, Long M, Schoenbach L, Roy SW, Gilbert W (1996). Intron positions correlate with module boundaries in ancient proteins. Proc Natl Acad Sci U S A.

[53] Bonnet A, Grosso AR, Elkaoutari A, Coleno E, Presle A, Sridhara SC (2017). Introns protect eukaryotic genomes from transcription-associated genetic instability. Mol Cell.

[54] Baskerville S, Bartel DP (2005). Microarray profiling of microRNAs reveals frequent coexpression with neighboring miRNAs and host genes. RNA.

[55] Brown JWS, Marshall DF, Echeverria M (2008). Intronic noncoding RNAs and splicing. Trends Plant Sci.

[56] Rearick D, Prakash A, McSweeny A, Shepard SS, Fedorova L, Fedorov A (2011). Critical association of ncRNA with introns. Nucleic Acids Res.

[57] Valencia P, Dias AP, Reed R (2008). Splicing promotes rapid and efficient mRNA export in mammalian cells. Proc Natl Acad Sci U S A.

[58] Callis J, Fromm M, Walbot V (1987). Introns increase gene expression in cultured maize cells. Genes Dev.

[59] Eghbalnia HR, Wilfinger WW, Mackey K, Chomczynski P (2020). Coordinated analysis of exon and intron data reveals novel differential gene expression changes. Sci Rep.

[60] Parenteau J, Abou ES (2019). Introns: Good Day Junk Is Bad Day Treasure. Trends Genet.

[61] Jo B-S, Choi SS (2015). Introns: The Functional Benefits of Introns in Genomes. Genomics Inform.

[62] Gotic I, Omidi S, Fleury-Olela F, Molina N, Naef F, Schibler U (2016). Temperature regulates splicing efficiency of the cold-inducible RNA-binding protein gene Cirbp. Genes Dev.

[63] James AB, Calixto CPG, Tzioutziou NA, Guo W, Zhang R, Simpson CG (2018). How does temperature affect splicing events? Isoform switching of splicing factors regulates splicing of LATE ELONGATED HYPOCOTYL (LHY). Plant Cell Environ.

[64] Airoldi CA, McKay M, Davies B (2015). MAF2 Is Regulated by Temperature-Dependent Splicing and Represses Flowering at Low Temperatures in Parallel with FLM. PLoS ONE.

[65] Evantal N, Anduaga AM, Bartok O, Patop IL, Weiss R, Kadener S. Thermosensitive alternative splicing senses and mediates temperature adaptation in Drosophila. bioRxiv 2018. doi: 10.7554/eLife.44642. 10.7554/eLife.44642 PMC6890466 31702556

[66] Teich R, Grauvogel C, Petersen J (2007). Intron distribution in Plantae: 500 million years of stasis during land plant evolution. Gene.

[67] Thonig A, Oellermann M, Lieb B, Mark FC (2014). A new haemocyanin in cuttlefish (Sepia officinalis) eggs: Sequence analysis and relevance during ontogeny. Evodevo.

[68] Kearse M, Moir R, Wilson A, Stones-Havas S, Cheung M, Sturrock S (2012). Geneious Basic: an integrated and extendable desktop software platform for the organization and analysis of sequence data. Bioinformatics.

[69] Davison A, McDowell GS, Holden JM, Johnson HF, Koutsovoulos GD, Liu MM (2016). Formin Is Associated with Left-Right Asymmetry in the Pond Snail and the Frog. Curr Biol.

[70] Birney E, Clamp M, Durbin R (2004). GeneWise and Genomewise. Genome Res.

[71] Streit K-S (2008). Differentielle Expression und molekulare Evolution von Mollusken-Hämocyanin [Dissertation].

[72] Kent WJ, Sugnet CW, Furey TS, Roskin KM, Pringle TH, Zahler AM, Haussler AD (2002). The Human Genome Browser at UCSC. Genome Res..

[73] Lieb B, Boisguerin V, Gebauer W, Markl J (2004). cDNA sequence, protein structure, and evolution of the single hemocyanin from Aplysia californica, an opisthobranch gastropod. J Mol Evol.

[74] Altschul SF, Gish W, Miller W, Myers EW, Lipman DJ (1990). Basic local alignment search tool. J Mol Biol.

[75] Kumar S, Stecher G, Tamura K (2016). MEGA7: Molecular Evolutionary Genetics Analysis Version 7.0 for Bigger Datasets. Mol Biol Evol..

[76] Edgar RC (2004). MUSCLE: multiple sequence alignment with high accuracy and high throughput. Nucleic Acids Res.

[77] Pettersen EF, Goddard TD, Huang CC, Couch GS, Greenblatt DM, Meng EC, Ferrin TE (2004). UCSF Chimera–a visualization system for exploratory research and analysis. J Comput Chem.

[78] Crooks GE, Hon G, Chandonia J-M, Brenner SE (2004). WebLogo: a sequence logo generator. Genome Res.

[79] Xu L, Dong Z, Fang L, Luo Y, Wei Z, Guo H (2019). OrthoVenn2: a web server for whole-genome comparison and annotation of orthologous clusters across multiple species. Nucleic Acids Res.

