# Supplementary material for: The evolution of hemocyanin genes in Tectipleura: a multitude of conserved introns in highly diverse gastropods
Source: BMC Ecol Evol. 2021 Mar 4;21:36. doi: 10.1186/s12862-021-01763-3 (PMC7931591; doi:10.1186/s12862-021-01763-3)
Supplement: Supplementary file 1 — Additional file 1: Figure S1. Hemocyanin gene structures. [file 12862_2021_1763_MOESM1_ESM.pdf]

Additional file 1

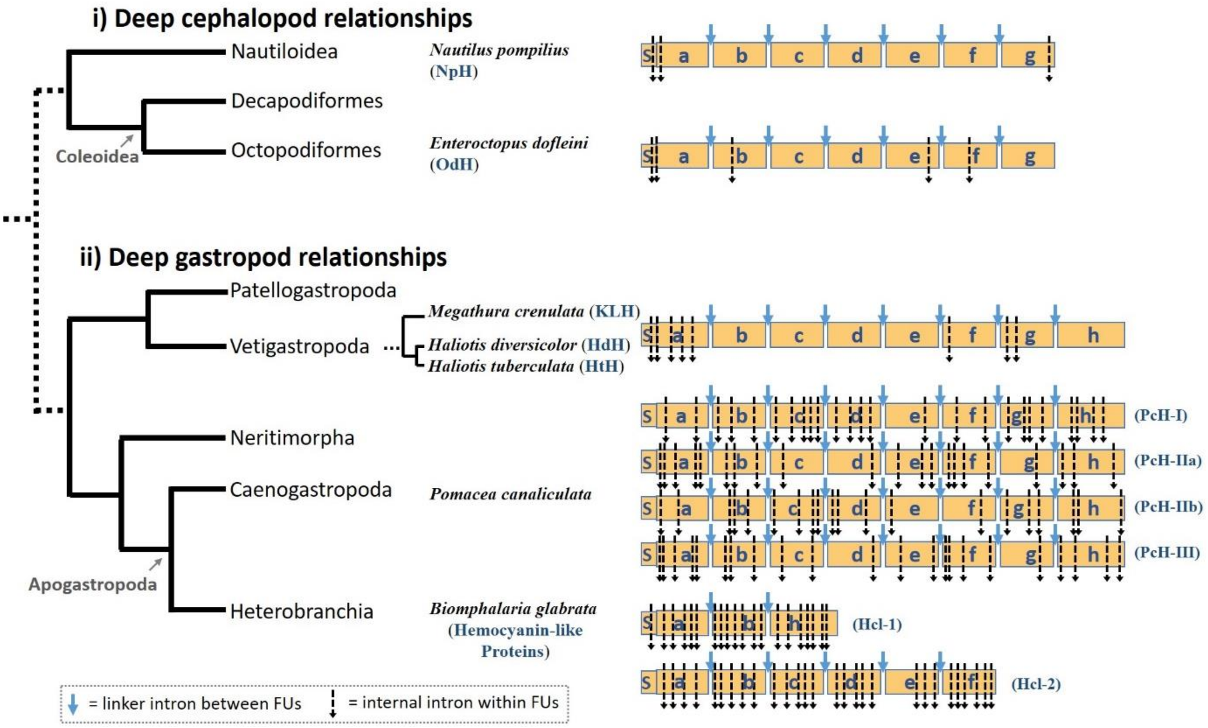

Figure S1: Hemocyanin gene structures. Species of gastropods and cephalopods with known hemocyanin gene structures and their relationships are shown on the left side. Exon-intron architectures of their hemocyanins (including hemocyanin-like proteins) are shown on the right site. Orange boxes represent the different functional units FU-a to FU-h and the signal peptide S. Blue arrows symbolize splice sites of linker introns that lie between the FUs (conserved at the same position for all species). Dotted black arrows symbolize splice sites of internal introns. Their number and positions vary among the different gene structures.
