# Supplementary material for: The evolution of hemocyanin genes in Tectipleura: a multitude of conserved introns in highly diverse gastropods
Source: BMC Ecol Evol. 2021 Mar 4;21:36. doi: 10.1186/s12862-021-01763-3 (PMC7931591; doi:10.1186/s12862-021-01763-3)
Supplement: Supplementary file 2 — Additional file 2: Figure S2.Sequence comparison of hemocyanin-like proteins of Biomphalaria glabrata with hemocyanins of gastropods and cephalopods. [file 12862_2021_1763_MOESM2_ESM.pdf]

<u>Additional file 2</u>

Figure S2: Sequence comparison of hemocyanin-like proteins of *Biomphalaria glabrata* with hemocyanins of gastropods and cephalopods. The amino acid sequences of functional units included in this multiple sequence alignment come from hemocyanins of Tectipleura, Lepetellida and Cephalopoda (abbreviations see below). Colors indicate conservation scores: Red/blue: conservation of at least 95%/80% over all FUs; grey: FU-specific conservation of 95% (e.g. within FU-a, FU-b, …); yellow: exception of BgHcl1/2 sequence to conservations described above. “X” symbolizes not known amino acids. Signal peptides as well as tail extensions of FU-h are not included. Included hemocyanins: AcH1+2 (*Aplysia californica*), LsH1+2 (*Lymnaea stagnalis*), BgHcl1+2 (*Biomphalaria glabrata* hemocyanin like proteins), HpHaD + HpHaN + HpHb (*Helix pomatia* alphaD, alphaN, beta), CaHaD CaHaN CaHb (*Cornu aspersum* alphaD, alphaN, beta); HtH1+2 (*Haliotis tubercularа*), KLH1+2 (*Megathura crenulata*), OdHG + OdHA (*Octopus vulgaris*), NpH (*Nautilus pompilius*).

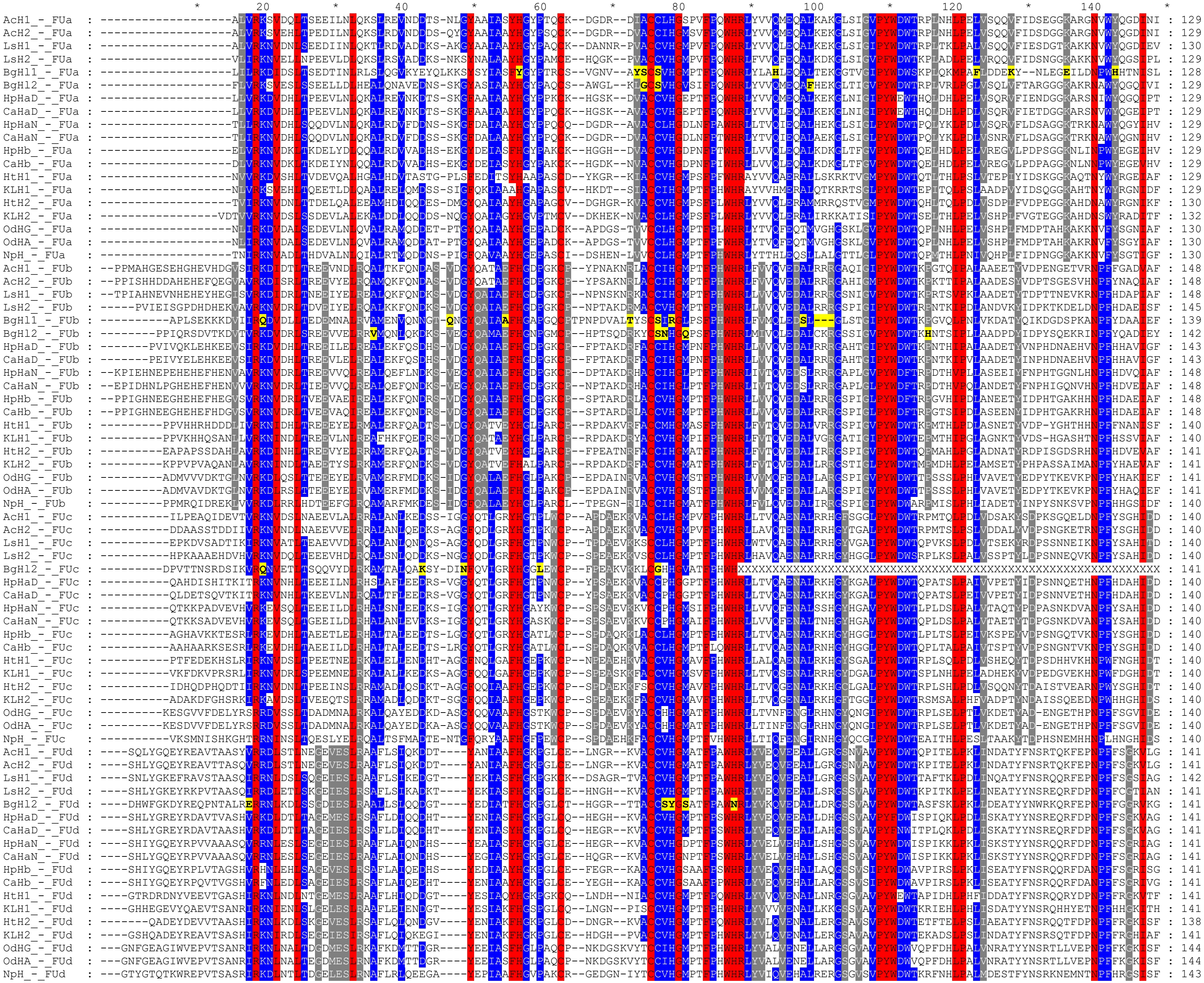

AcH1_-_FUa : 129
AcH2_-_FUa : 129
LsH1_-_FUa : 130
LsH2_-_FUa : 129
BgH11_-_FUa : 128
BgH12_-_FUa : 129
HpHaD_-_FUa : 129
CaHaD_-_FUa : 129
HpHaN_-_FUa : 129
CaHaN_-_FUa : 129
HpHb_-_FUa : 129
CaHb_-_FUa : 129
HtH1_-_FUa : 129
KLH1_-_FUa : 129
HtH2_-_FUa : 130
KLH2_-_FUa : 132
OdHG_-_FUa : 130
OdHA_-_FUa : 130
NpH_-_FUa : 130
AcH1_-_FUb : 148
AcH2_-_FUb : 148
LsH1_-_FUb : 148
LsH2_-_FUb : 145
BgH11_-_FUb : 139
BgH12_-_FUb : 142
HpHaD_-_FUb : 143
CaHaD_-_FUb : 143
HpHaN_-_FUb : 148
CaHaN_-_FUb : 148
HpHb_-_FUb : 148
CaHb_-_FUb : 148
HtH1_-_FUb : 140
KLH1_-_FUb : 140
HtH2_-_FUb : 141
KLH2_-_FUb : 141
OdHG_-_FUb : 141
OdHA_-_FUb : 141
NpH_-_FUb : 140
AcH1_-_FUc : 140
AcH2_-_FUc : 140
LsH1_-_FUc : 140
LsH2_-_FUc : 140
BgH12_-_FUc : 141
HpHaD_-_FUc : 140
CaHaD_-_FUc : 140
HpHaN_-_FUc : 140
CaHaN_-_FUc : 140
HpHb_-_FUc : 140
CaHb_-_FUc : 140
HtH1_-_FUc : 140
KLH1_-_FUc : 140
HtH2_-_FUc : 140
KLH2_-_FUc : 140
OdHG_-_FUc : 140
OdHA_-_FUc : 140
NpH_-_FUc : 140
AcH1_-_FUd : 141
AcH2_-_FUd : 141
LsH1_-_FUd : 142
LsH2_-_FUd : 141
BgH12_-_FUd : 141
HpHaD_-_FUd : 141
CaHaD_-_FUd : 141
HpHaN_-_FUd : 141
CaHaN_-_FUd : 141
HpHb_-_FUd : 141
CaHb_-_FUd : 141
HtH1_-_FUd : 141
KLH1_-_FUd : 141
HtH2_-_FUd : 138
KLH2_-_FUd : 141
OdHG_-_FUd : 144
OdHA_-_FUd : 144
NpH_-_FUd : 143

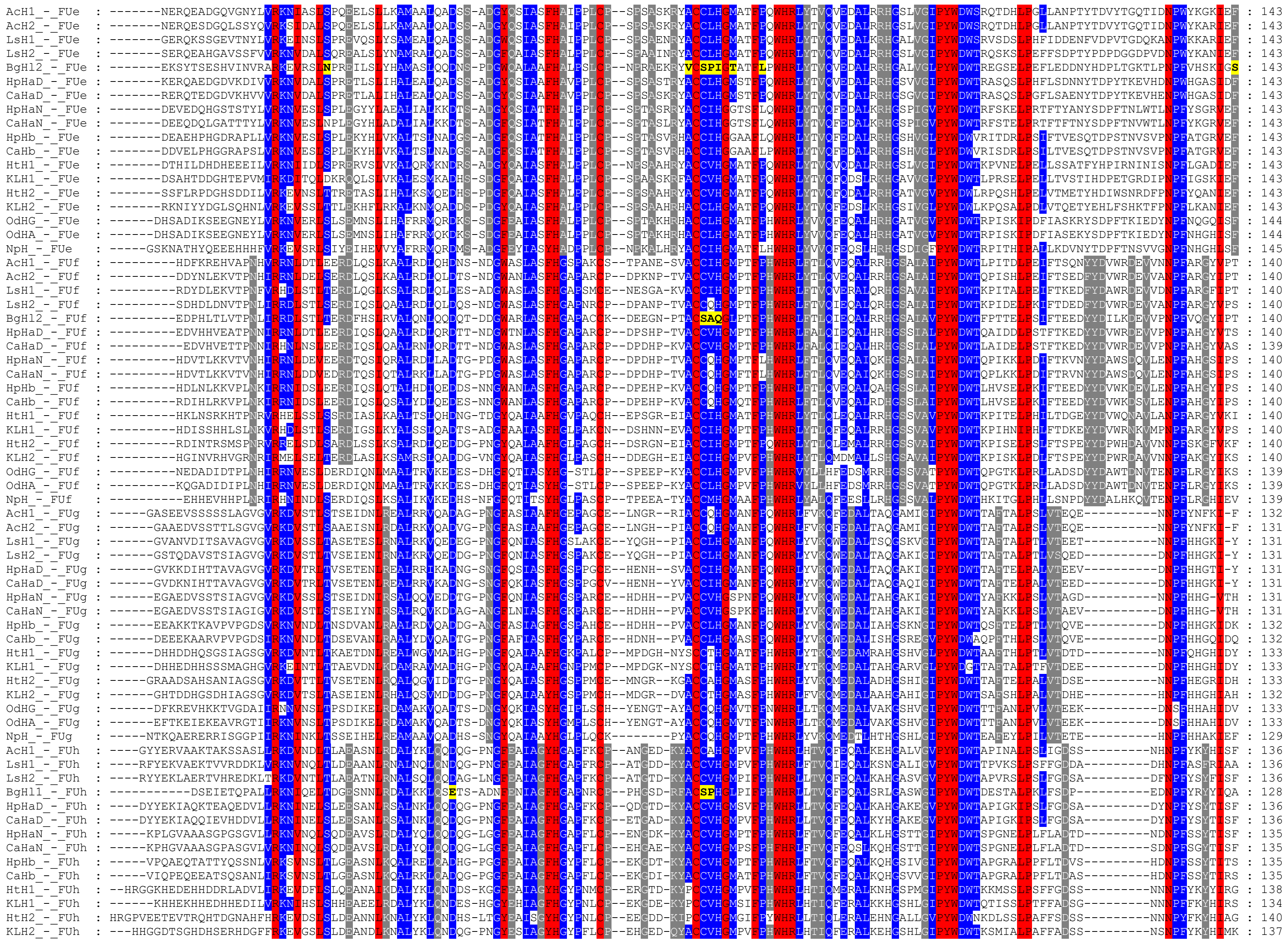

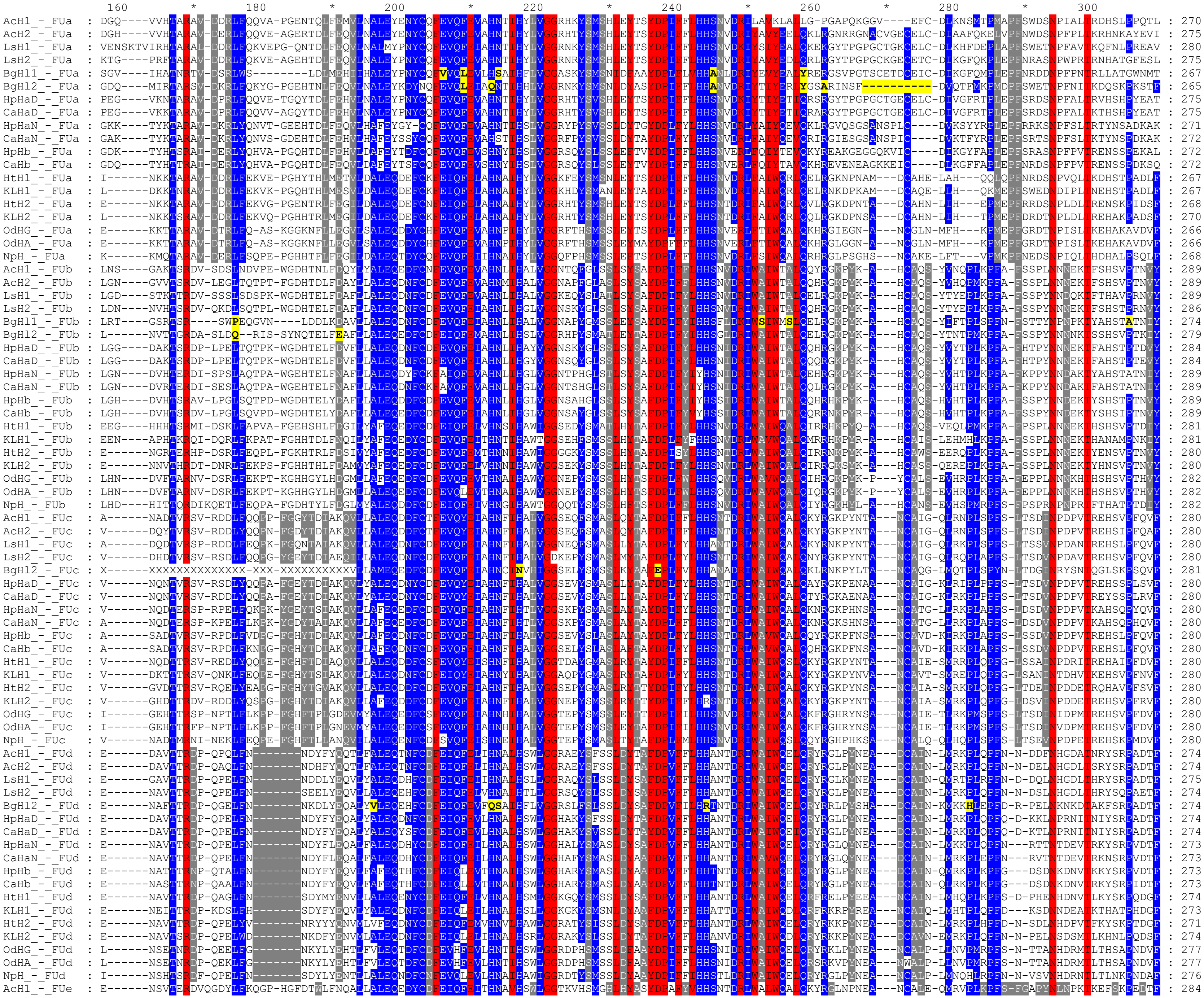

```
AcH2_-_FUe  : E------NTTTERDVQGDYLFKQGP-HGFDTWLFNQALLALEQEDYCDFEIQFEITHNAVHSWLGGTKVHSMGHLHYASYDPAFYVHHSNTDRLWALWQALQKYRGHNPNEA---NCALE-QMREPLKPFS-FGSPYNLNEMTKEYSKPEDTF : 284
LsH1_-_FUe  : E------NSETEREVVSDRLFKRGP-HGWDTWLFNQALLALEQEDYCNFEIQFEITHNAIHSWVGGSKEHSLGHLHYASYDPAFFVHHSNTDRLWAVWQALQKYRGYDPNEA---NCALE-QMKDPLKPFS-FGPPYNLNPLTKEHSRPEDTF : 284
LsH2_-_FUe  : E------HAVTERDVKEEFLFKVGK-HGWDTWLFNQALYALEQEDYCDFEVQFEITHNAIHSWLGGSKNYSLGHLHYASYDPAFFIHHSNTDRLWAVWQALQKYRGHDPNEA---NCALE-LMREPLKPFS-FGPPYNLNQMTQDNSRPEDTF : 284
BgHl2_-_FUe : E------AVYTDRDILVDKMFKMGP-LGWDTWMFEQALFALEQDNYCDFQVQLEVISQVLSVWIGGSETHSMSHLHFASYDPLFFVHHANVDRLWAVWQALQKHRGHDPDKS---NCALE-MMKKPLKPFS-FGSPYNLNEVTREKSLPTAVF : 284
HpHaD_-_FUe : E------HSHTERDIQSAELFKLGP-HGWDTWLFEQALLALEQEDYCDFEIQFEITHNAIHSWVGGSKEHSLAHLHYASYDPAFYIHHSNTDRLWAIWQALQKHRGYSPNEA---NCALE-HLKDSLKPFS-FGPPYNLNKLTTKYSHPEDTF : 284
CaHaD_-_FUe : E------NSHTERDIQSAELFKLGP-HGWDTWLFEQALLALEQEDYCDFEIQFEITHNAIHSWVGGSKEHSLAHLHYASYDPAFYIHHSNTDRLWAIWQALQKHRGYNPNEA---NCALE-HIKDSLKPFS-FGPPYNLNKLTTKYSHPEDTF : 284
HpHaN_-_FUe : E------HVDTERDVQEDKLFKRGP-HGWDTWLYTQVLFALEQEDFCDFEVQLEIAHNAIHSWIGGSKEHSLAHLHYASYDPAFFIHHSNTDRLFAVWQKLQTYRGHDANSA---NCALE-QLREPLKPFS-FGPPYNLNKVTQDNSRPEDTF : 284
CaHaN_-_FUe : E------HVDTERDVQEDKLFKSGPH-GWDTWLYTQVLFALEQEDFCDFEVQLEIAHNAIHSWIGGSKEHSLAHLHYASYDPAFFIHHSNTDRLFAVWQKLQQYRGHDPNSA---NCALE-QLREPLKPFS-FGPPYNLNKVTQEYSRPQDTF : 284
HpHb_-_FUe  : E------HEDVHRDI-QSNLLFKRGPHGWDTWLYNQALYALEQEDYCDFAVQLEITHNSIHAWIGGSQKHSMAHLHYASYDPIFFIHHSNTDRLWAVWQALQEYRGHSANEV---NCALE-KLREPLKPFS-FGPPYNLNPVTLENSHPGDTF : 284
CaHb_-_FUe  : E------HEDVHRDI-QSDLLFKRGPHGWDTWLYNQALYALEQEDYCDFAVQLEITHNSIHAWIGGSQKHSMAHLHYASYDPIFFIHHSNTDRLWAVWQALQKHRGHNAYEV---YCSLE-KLREPLKPFS-FGPPYNLNPVTRQNAHSGDTF : 284
HtH1_-_FUe  : EGP----GVHTERHINTERLFHSGDHDGYHNWFFETVLFALEQEDYCDFEIQFEIAHNGIHTWIGGSAVHGMGHLHYASYDPIFYIHHSQTDRIWAIWQELQKYRGLSGSEA---NCAIE-HMRTPLKPFS-FGPPYNLNSHTQEYSKPEDTF : 287
KLH1_-_FUe  : EGE----NVHTKRDINRDRLFQGST-KTHHNWFIEQALLALEQTNYCDFEVQFEIMHNGVHTWVGGKEPYGIGHLHYASYDPLFYIHHSQTDRIWAIWQSLQRFRGLSGSEA---NCAVN-LMKTPLKPFS-FGAPYNLNDHTHDFSKPEDTF : 286
HtH2_-_FUe  : EGE----NITTEREVIADKLFVKGG-HVFDNWFFKQAILALEQENYCDFEIQFEILHNGVHTWVGGSRTYSIGHLHYAFYDPLFYLHHSQTDRIWAIWQELQEQRGLSGDEA---HCALE-QMREPLKPFS-FGAPYNWNQLTQDFSRPEDTF : 286
KLH2_-_FUe  : EGE----GVTTERDVDAEHLFAKGN-LVYNNWFCNQALYALEQENYCDFEIQFEILHNGIHSWVGGSKTHSIGHLHYASYDPLFYIHHSQTDRIWAIWQALQEHRGLSGKEA---HCALE-QMKDPLKPFS-FGSPYNLNKRTQEFSKPEDTF : 286
OdHG_-_FUe  : ISE----DTETKREV-SEYLFEHPV-LGKQTWLFDNIALALEQTDYCDFEIQLEIVHNAIHSWIGGKEEHSLNHLHYAAYDPIFYLHHSNVDRLWVIWQELQKLRGLNAYES---HCALE-LMKVPLKPFS-FGAPYNLNDLTTKLSKPEDMF : 286
OdHA_-_FUe  : ISE----DTETKREV-SEYLFEHPV-LGKQTWLFDNIALALEQTDYCDFEIQLEIVHNAIHSWIGGKEEHSLNHLHYAAYDPIFYLHHSNVDRLWVIWQELQKLRGLNAYES---HCALE-LMKVPLKPFS-FGAPYNLNDLTTKLSKPEDMF : 286
NpH_-_FUe   : ISP----EVMTQRDV-QDKLFKQPK-LGRNTWLHDNVILALEQTDYCNFEVQFEIVHNAIHAWVGGSQEHSLSHLHFAAYDPAFYLHHSNVDRIWVIWQELQKIRGLSAYEA---NCALE-LMNEPLKPFA-FGSPYNLNEMTRKYSLPEDTF : 287
AcH1_-_FUf  : E------DVYTVRDI-RPEIRNKNQ-AGDHSAIFDLVLSALEQTDYCDFEVQFEVMHNAIHFLVGGLQTYSLSSLEYSAYDPLFFIHHSFTDKIWAVWQELQTRRHLPANKA---DCALN-YMSQPMRPFF-F-EGFNLNKFTMDHAVPNTVF : 279
AcH2_-_FUf  : E------KAYTVRDV-RPEIHLNDR-EGRHSAIFDLVLLALEQTDYCDFEVQFEVMHNAIHFLVGGLQTYSMSTLEYSAYDPMFLIHHSFTDKIWIVWQELQKRRHLPATSA---ACAVN-YMNQPMKPFY-F-EGFNLNGFTKSHALPSSVF : 279
LsH1_-_FUf  : E------NTYTVRDI-RPELFQTSK-DGSHSDIFELVLSALEQTDYCDFEVQFEVMHNAIHYLVGGLQTYSLSSLEYSAYDPIFFIHHSFVDKIWVVWQELQQRRHLPSNTA---NCALN-YLAEPMKPFN---LDLNINAFTKKHAVPRDVF : 278
LsH2_-_FUf  : E------KVYTVRET-KSELYDKGR-GGKNSAIIYYVISALEQTDYCDFEVQFEVIHNSIHYLVGGEQTYSLSSLEYSAYDPIFFIHHSFTDKIWAVWQELQKRRRLPYLKA---NCALN-YMAEPMKPFS-I-DGLNLNKFTKEHAVPDKLF : 279
BgHl2_-_FUf : E------DTYTVRDV-RHELFXXXX-XXXXXXXXXXXXXXXXXXXXXXXXAQFQVVHSTILNSLGGDQLYSLSTLEYSAYDPIFIIHNSFMDKIWLIWQELQKERHLPSLGT---ECALN-YMNQPLQPFN---SELNMNTFTKEHSLPKDLF : 278
HpHaD_-_FUf : E------DTYTVRDI-QETLHDRHV-DGKHSFLLYGVLEVLEQTDYCDFEVHFEVVHNAIHYLIGGHQTYSLSSLEYSAYDPIFFIHHSFTDKLWAVWQELQKRRHFPYNRA---DCALN-YVNEPLKPFS-F-EGFNLNKFTREHAVPNTLF : 279
CaHaD_-_FUf : E------DTYTVRDI-QSPLHK-KH-DGKHSFLLHGVLDVLEQTDYCDFEVQFEVVHNAIHYLIGGHQTYSLSSLEYSAYDPIFFIHHSFTDKIWAVWQELQKRRNLPYNRA---DCALN-YVNEPLKPFS-F-EGLNLNKFTREHAVPNTLF : 277
HpHaN_-_FUf : E------KAQTVRDV-QPELFETTK-DGKHSTLFPLLLEALEQTSYCDFAVQFEVLHNAIHYLVGGHQKYSLSSLEYSAYDPIFFIHHSFSDKLWVVWQELQQRRHLPYHTA---DCAVS-AMSEPMKPFS-F-ETFNTNKFTRDHAGPNSLF : 279
CaHaN_-_FUf : E------KAQTVRDV-QPELFETTE-DGKHSTLFPLLLEALEQTSYCDFAVQFEVLHNAIHYLVGGHQKYSLSSLEYSAYDPIFFIHHSFSDKLWVVWQELQQRRHLPYHTA---DCAVN-AMSEPMKPFS-F-ETFNTNKFTREHSVPNTVF : 279
HpHb_-_FUf  : E------GAYTVRDV-YQHFFRAYG-DGKHSFLFYIALEALEQTDYCDFEVQFEILHNAIHYLVGGEQKYSLSSLEYSAYDPLFFIHHSFVDKIWVVWQKLQQRRHLPYNRA---DCAVA-AVNQPMKPFT-F-ESINPNKFTREHALPSSVF : 279
CaHb_-_FUf  : E------DAYTVRDV-YPGFYKSFG-DGKHSFLFIIALQALEETDYCNFEVQFEILHNAIHYIVGGEQKYSLSSLEYSAYDPLFFIHHSFTDKIWVVWQELQQRRHLPYNRA---DCAVV-AMNQPMKPFT-F-ESLNLNKFTREHALPSSVF : 279
HtH1_-_FUf  : K------DAFTVRNV-QESLFKMSS-FGKHSLLFDQALLALEQTDYCDFEVQFEVMHNTIHYLVGGRQTYAFSSLEYSSYDPIFFIHHSFVDKIWAIWQELQSRRHLQFRTA---DCAVG-LMGQAMRPFN---KDFNHNSFTKKHAVPNTVF : 278
KLH1_-_FUf  : H------DTYTVRDV-QEGLFHLTS-TGEHSALLNQALLALEQHDYCDFAVQFEVMHNTIHYLVGGPQVYSLSSLHYASYDPIFFIHHSFVDKVWAVWQALQEKRGLPSDRA---DCAVS-LMTQNMRPFH-Y--EINHNQFTKKHAVPNDVF : 278
HtH2_-_FUf  : A------NTYTVRDP-QEMLFQLCE-HG-ESILYEQTLLALEQTDYCDFEVQFEVLHNVIHYLVGGRQTYALSSLHYASYDPFFFIHHSFVDKMWVVWQALQKRRKLPYKRA---DCAVN-LMTKPMRPFD---SDMNQNPFTKMHAVPNTLY : 277
KLH2_-_FUf  : E------DAYTVRDP-QDILYHLQD-ETGTSVLLDQTLLALEQTDFCDFEVQFEVVHNAIHYLVGGRQVYALSSQHYASYDPAFFIHHSFVDKIWAVWQALQKKRKRPYHKA---DCAIN-MMTKPMRPFA---HDFNHNGFTKMHAVPNTLF : 278
OdHG_-_FUf  : E------DTYTVRDV-KPELFE-IG-GGEGSTLYQQVLLMLEQEDYCDFEVQFEVVHNSIHYLVGGHHKYAMSSLVYSSFDPIFYVHHSMVDRLWAIWQALQEHRHLPFDKA---YCALE-QLSFPMKPFV---WDSNPNLHTRAASTPQHLF : 276
OdHA_-_FUf  : E------DTYTVRDV-KPELFE-IG-GGEGSTLYRQVLLMLEQEDYCDFEVQFEVVHNSIHYLVGGHQKYAMSSLVYSSFDPIFYVHHSMVDRLWAIWQALQEHRHLPFDKA---YCALE-QLSFPMKPFV---WESNPNLHTRAASTPQHLF : 276
NpH_-_FUf   : A------ETYTVRNT-QPELFEPSK-DGKHSLLYDEILLALEQRDYCDFEVQFEVVHNAIHYLVGGHQRYSMSSLEYASYDPLFFIHHSNVDRLWAIWQALQQYRRIPYNKA---YCALE-QMTHAMKPFD---RETNPNKNTHDHAIPNSVF : 277
AcH1_-_FUg  : N------GEVTSRAP-REQLFNDPE-FGSESFFYRQALLAFEQTDYCDFEVQYEITHNAIHSWTGGRSPYGMSTLEFTAYDPLFLLHHSNADRQFAIWQALQKFRGLPYNNA---NCAIQ-KLREPMKPFS---NLDNINPTTRANARAIDAF : 270
AcH2_-_FUg  : N------GEVTSRAP-REQLFNDPE-FGSESFFYRQALLAFEQTDYCDFEVQYEITHNAIHSWTGGRSPYGMSTLEFTAYDPLFLLHHSNVDRQFAIWQALQKFRGLPYNNA---NCAIQ-KLREPMKPFS---NSDNINPTTRANARATDAF : 269
LsH1_-_FUg  : N------GEITTRAP-RDQLFNDPE-FGSESFFYRQALFTFEQTDYCNFEVQFEITHNAIHSWTGGSSQYGMSSLEYTAYDPLFLLHHSNVDRQFAIWQALQKFRGLPYNSA---NCAIQ-HLRQPMRPFS---DENNLNAITRANSRAIDAF : 269
LsH2_-_FUg  : N------GEVTTRAP-RDKLFNDPE-FGAESFFYRQVLLAFEQTDYCDFEVQFEITHNAIHSWTGGSSPYGMSTLEYTAYDPLFLLHHSNVDRQFAIWQALQKFRGLPYNSA---NCAIQ-HLRQPMRPFS---DESNINAITRANSRAIDAF : 269
HpHaD_-_FUg : N------GEITTRAP-RDKLFNDPE-FGKESFFYRQVLLAFEQTDYCDFEVQYEISHNAIHSWTGGQSPYGMSTLEYTAYDPLFLLHHSNVDRQFAIWQALQKFRGLPYNSA---NCAIQ-LLHQPMRPFS---DADNVNPVTRTNSRARDVF : 269
CaHaD_-_FUg : N------GQNTTRAP-RGRLFNHPE-FGRESFFYRQILLAFEQTDYCDFEVQYEISHNAIHSWTGGKSPYGMSTLEYTAYDPLFLLHHSNVDRQFAIWQALQKFRGLPYNSA---NCAIQ-LLHQPMRPFS---DSDNINPVTRTNSRARDVF : 269
HpHaN_-_FUg : D------GHITTRAP-RSLLFNDPE-FGDESFFYRQVLLAFEQTDYCDFEVQFEITHNAIHSWVGGQSPYGLSTLEYTAYDPLFLLHHSNVDRQFSIWQALQKYRGLPYNTA---NCAIQ-DLRRPLRPFS---DPANVNPTTRANSRAIDAF : 269
CaHaN_-_FUg : D------GHVTTRAP-RSLLFNDPE-FGDESFFYRQVLLAFEQTDYCDFEVQFEITHNAIHSWVGGQSPYGLSTLEYTAYDPLFLLHHSNVDRQFAIWQALQKHRGLPYNTA---NCAIQ-ELRRPLRPFS---DKENVNPTTRANSRAIDAF : 269
HpHb_-_FUg  : -------DHNTTRSP-RPQLFSDPA-SGNESFFYRQVLLAFEQTDYCDFEVQFEFAHNAIHSWTGGRSPYGMSTLEYTAYDPLFLLHHSNVDRQFAIWQALQKFRGLPYNSA---NCAIQ-LLHQPMRPFS---DSDNVNPTTRAHSTASEAF : 269
CaHb_-_FUg  : -------DHNTTRSP-RPQLFSDPE-HGEESFFYRQVLLAFEQTDYCDFEVQFEFAHNAIHSWIGGQSPYSMSTLEYTAYDPLFLLHHSNVDRQFAIWQALQKFRGLPYNSA---NCAIQ-LLHQPMRPFS---DADNVNPTTRSHSTASEAF : 269
HtH1_-_FUg  : L------NVSTTRSP-RDMLFNDPE-HGSESFFYRQVLLALEQTDFCKFEVQFEITHNAIHSWTGGHSPYGMSTLDFTAYDPLFWLHHSNTDRIWAVWQALQEYRGLPYNHA---NCEIQ-AMKTPLRPFS---DDINHNPVTKANAKPLDVF : 271
KLH1_-_FUg  : L------GVDTTRSP-RDKLFNDPE-RGSESFFYRQVLLALEQTDFCQFEVQFEITHNAIHSWTGGLTPYGMSTLEYTTYDPLFWLHHANTDRIWAIWQALQEYRGLPYDHA---NCEIQ-AMKRPLRPFS---DPINHNAFTHSNAKPTDVF : 271
HtH2_-_FUg  : L------GVTTSRSP-RDMLFNDPE-QGSESFFYRQVLLALEQTDYCQFEVQFEITHNAIHSWTGGRSPYGMSTLEFTAYDPLFWLHHSNTDRIWAVWQALQKYRGLPYNEA---HCEIQ-VLKQPLRPFN---DDINHNPITKTNARPIDSF : 271
KLH2_-_FUg  : R------NVDTSRSP-RDMLFNDPE-HGSESFFYRQVLLALEQTDFCQFEVQFEITHNAIHSWTGGHIPYGMSSLEYTAYDPLFYLHHSNTDRIWAIWQALQKYRGFQYNAA---HCDIQ-VLKQPLKPFS---ESRNPNPVTRANSRAVDSF : 270
OdHG_-_FUg  : A------NTDTTRSP-RAQLFDDPE-KGDKSFFYRQIALALEQTDFCDFEIQFEIGHNAIHSWVGGSSPYGMSTLHYTSYDPLFYLHHSNTDRIWSVWQALQKYRGLPYNTA---NCEIN-KLVKPLKPFN---LDTNPNAVTKAHSTGATSF : 271
OdHA_-_FUg  : A------NTDTTRSP-RAQLFDDPE-KGDKSFFYRQIALALEQTDFCDFEIQFEIGHNAIHSWVGGSSPYGMSTLHYTSYDPLFYLHHSNTDRIWSVWQALQKYRGLPYNTA---NCEIN-KLVKPLKPFN---LDTNPNAVTKAHSTGATSF : 271
NpH_-_FUg   : I------NETTSRNP-RPQLFTDQR-YGETSFFYRQILLALEQTDFCDFEIQFEVGHNAIHSWVGGSTQYGMSTLHFTSYDPLFYLHHSNTDRIWAIWQALQRYRGLPYNSA---NCEIH-LLKKPLEPFS---FESNPNHETHNHSTGNAVF : 267
AcH1_-_FUh  : V------NQDTTRDI-QDSLFNPRT-INGYNYLYYLALSTLEEDSFCDFEIQYEILHNEIHGLIGGHGTYSMSTLDYSAFDPLFMIHHSSIDRIWAIWQQLQKLRRKPFNSA---RCGGA-IMEEPLQPFS-Y-SQINTNDFTRMNSQPSKVF : 275
LsH1_-_FUh  : A------NEKTTRQV-QSELYSERK-VHGFPYLFYLALTTLEEDNYCDFEVQFEVLHNEIHADIGGSGTYSMATLDYSAFDPFFMIHHSSIDRIWVIWQELQKLRHKPFNGA---SCAGH-IMERPLQPFS-Y-PEVNKNEFTRLNSVPNVVF : 275
LsH2_-_FUh  : V------GKKTTRDV-KDELFNPPT-INGFNQLYYLALSTLEEDNYCDFEVQYEILHNQIHALIGGTGTYSMATLDYSAFDPFFMIHHSSIDRIWVIWQELQKIRKKPFNAA---HCGGH-LMETPLQPFS-Y-PEVNPNDLTRLNAVPNLVF : 275
BgHl1_-_FUh : E------KEWTDREV-NLKQLNLLD-PEGTKMLFHSALSILEEDQFCDFAVQFELLHYRLHALMGGTKKYSLATLDFSAFDPLFMILQSSFDRLWTMWQQLQYLRQKTVS-G---QCVYK-HVDSSMEPFR-N-PDINVNKMTRENSLPGLVS : 266
HpHaD_-_FUh : N------NQRTTRDI-QSELYNPHQ-INGYNYLYYLALSTLEEDNFCDFEVQYEVLHNEIHALIGGNGTYSMATLDFSAFDPFFMIHHSSLDRIWIIWQELQKLRHKPFNYA---HCGGH-VLDDPLHPFS-F-GEINKNDLTRLNSQPSSVF : 275
CaHaD_-_FUh : N------NQRTTRDI-QGELYNPHT-INGFNYIYYLALSTLEEDNFCDFEVQYEVLHNEIHALIGGNGTYSMATLDFSAFDPFFMIHHSSLDRIWIIWQELQKLRHKPFNYA---QCGGH-VLDDPLHPFS-F-GDINKNDLTRLNSLPSSVF : 275
HpHaN_-_FUh : V------GQRTSRNP-LGALFSTNT-SAGTSLLYQLTLDALEEDDYCHFEIMLEFLHNRIHFLIGGTETYSMSTLDYSAFDPIFMIVHSGMDRLWVLWQELQKLRRKPFVAI---ECGEK-SLHEPLHPFD---YDINTIALTREHAVPDTLF : 273
CaHaN_-_FUh : A------GQRTSRNP-LEALFSTNT-SAGTSLLYQLTLDALEEDDYCQFEIMLEFFHNRIHFLIGGSETYSMSTLDYSAFDPIFMIVHSGMDRLWVLWQELQKLRRKPFVAI---ECGEK-SLHEPLHPFD---YDINTISLTRENAVPDSIF : 273
HpHb_-_FUh  : A------GKTTTRSP-LEALFSANT-SRGHTILYDLTLDALEEEDYCHFEASLEFLHNRIHFFIGGTGTYSMSTLDYSAFDPLFMVVHSGMDRIWDLWEQLQKLRHKSYTPV---DCGGH-TFDKPLHPFD-F-PAINTIALTRQNAIPELLF : 274
CaHb_-_FUh  : V------GQTTTRSP-LQALFSANT-TQGHTILYDLTLNALEEEDYCHFETSLEFLHNRVHFFIGGSGTYSMSTLDYSAFDPLFMIVHSGMDRIWDLWEGLQKLRHKPYKPV---SCAGH-KFDKPLHPFD-F-PAINTIALTRQNAIPEQLF : 274
HtH1_-_FUh  : V------QHETTRDV-NQRLFNQTK-FGEFDYLYYLTLQVLEENSYCDFEVQYEILHNAVHSWLGGTGQYSMSTLEYSAFDPVFMIHHSSLDRIWILWQKLQKIRMKPYYAL---DCAGDRLMKDPLHPFN-Y-ETVNEDEFTRINSFPSILF : 278
KLH1_-_FUh  : I------NQDTVRDV-NEAIFQQTK-FGEFSSIFYLALQALEEDNYCDFEVQYEILHNEVHALIGGAEKYSMSTLEYSAFDPYFMIHHASLDKIWIIWQELQKRRVKPAHAG---SCAGD-IMHVPLHPFN-Y-ESVNNDDFTRENSLPNAVV : 273
HtH2_-_FUh  : V------GHDTVREP-TSLIYNQPQ-IHGYDYLYYLALTTLEENNYCDFEVQYEILHNAVHSWLGGSQKYSMSTLEYSAFDPVFMILHSGLDRLWIIWQELQKIRRKPYNFA---KCAYH-MMEEPLAPFS-Y-PSINQDEFTRANSKPSTVF : 279
KLH2_-_FUh  : A------GHDTARSP-SDLLFNQPQ-LHGYDYLYYLALSTLEEDNYCDFEVHYEILHNAVHLWLGGTETYSMSSLAFSAYDPVFMILHSGLDRLWIIWQELQKLRKKPYNAA---KCAGH-MMDEPLHPFN-Y-ESANHDSFTRANAKPSTVF : 276
```

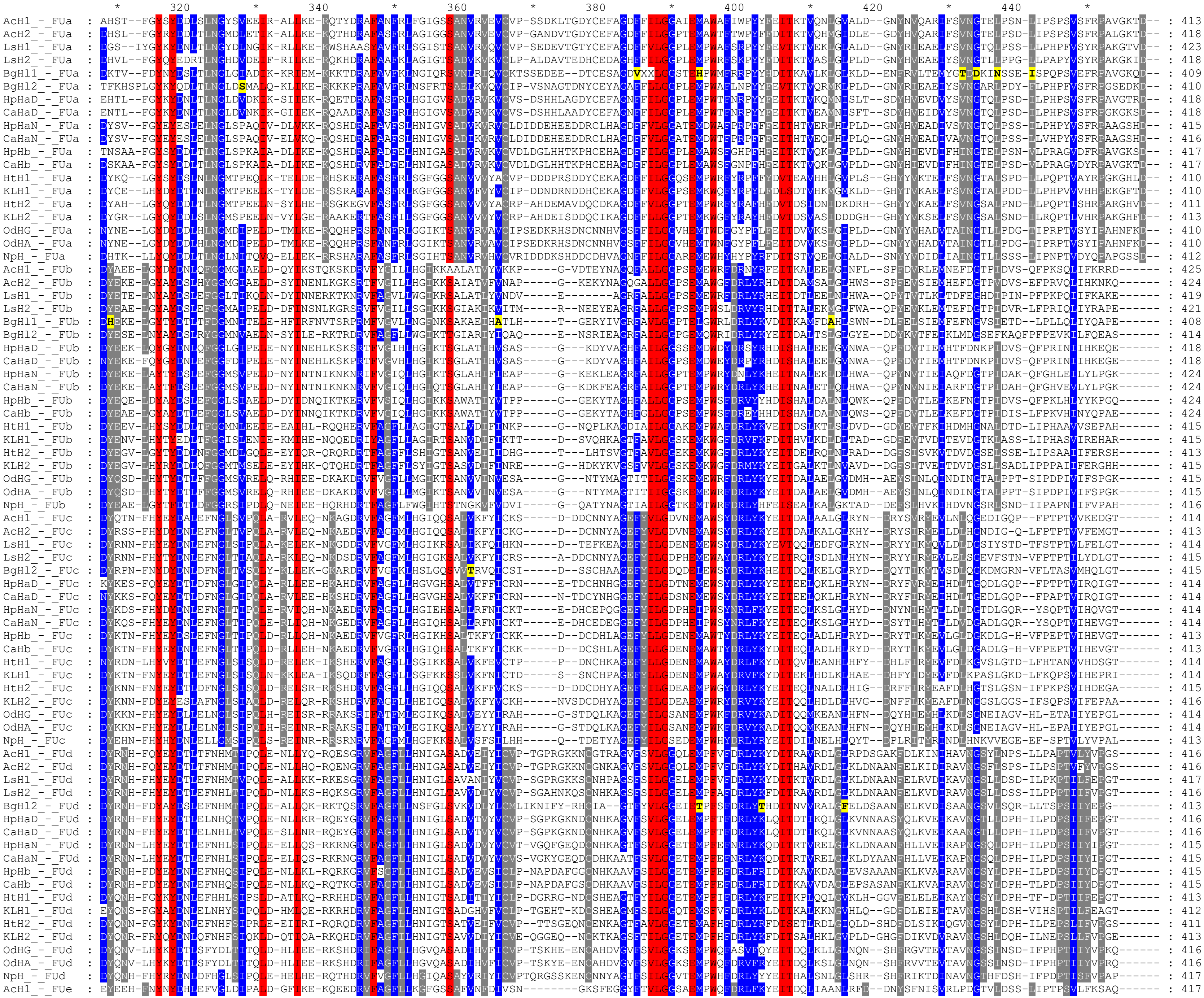
320
340
360
380
400
420
440
AcH1_-_FUa : 413
AcH2_-_FUa : 418
LsH1_-_FUa : 423
LsH2_-_FUa : 418
BgH11_-_FUa : 409
BgH12_-_FUa : 410
HpHaD_-_FUa : 418
CaHaD_-_FUa : 418
HpHaN_-_FUa : 415
CaHaN_-_FUa : 416
HpHb_-_FUa : 417
CaHb_-_FUa : 417
HtH1_-_FUa : 410
KLH1_-_FUa : 410
HtH2_-_FUa : 411
KLH2_-_FUa : 413
OdHG_-_FUa : 410
OdHA_-_FUa : 410
NpH_-_FUa : 412
AcH1_-_FUb : 425
AcH2_-_FUb : 424
LsH1_-_FUb : 419
LsH2_-_FUb : 421
BgH11_-_FUb : 408
BgH12_-_FUb : 414
HpHaD_-_FUb : 418
CaHaD_-_FUb : 418
HpHaN_-_FUb : 424
CaHaN_-_FUb : 424
HpHb_-_FUb : 424
CaHb_-_FUb : 424
HtH1_-_FUb : 415
KLH1_-_FUb : 415
HtH2_-_FUb : 413
KLH2_-_FUb : 415
OdHG_-_FUb : 415
OdHA_-_FUb : 415
NpH_-_FUb : 416
AcH1_-_FUc : 414
AcH2_-_FUc : 413
LsH1_-_FUc : 414
LsH2_-_FUc : 415
BgH12_-_FUc : 415
HpHaD_-_FUc : 414
CaHaD_-_FUc : 414
HpHaN_-_FUc : 414
CaHaN_-_FUc : 414
HpHb_-_FUc : 413
CaHb_-_FUc : 413
HtH1_-_FUc : 414
KLH1_-_FUc : 414
HtH2_-_FUc : 415
KLH2_-_FUc : 416
OdHG_-_FUc : 414
OdHA_-_FUc : 414
NpH_-_FUc : 413
AcH1_-_FUd : 416
AcH2_-_FUd : 416
LsH1_-_FUd : 417
LsH2_-_FUd : 416
BgH12_-_FUd : 413
HpHaD_-_FUd : 416
CaHaD_-_FUd : 416
HpHaN_-_FUd : 415
CaHaN_-_FUd : 415
HpHb_-_FUd : 415
CaHb_-_FUd : 415
HtH1_-_FUd : 413
KLH1_-_FUd : 412
HtH2_-_FUd : 411
KLH2_-_FUd : 413
OdHG_-_FUd : 416
OdHA_-_FUd : 416
NpH_-_FUd : 417
AcH1_-_FUe : 417

```
AcH2_-_FUe  : DYEGH-FNYRYDNLEFVGMNIPRLD-AFIKE-RRERSRVFAGFLLKGFGSSAMVTEDICTK-----GMESC-FQGGYFTVLGGAAEMPWQFDRLFKYEITDQLSEHNLRPD--DDYHFHVHVKYIDGTELDHS-LVPNPSVIIEPAS------ : 419
LsH1_-_FUe  : NYADH-FHYRYDNLEFVGLSIPQLD-AFIKE-RHEHDRVFAGFLLKGFKKSALVDEDICAN-------GNC-FPGGYFTVLGGSAEMPWQFDRLFKYEITDQLNEHKLRFD--DEYTFQIKIRAPDGTELSSD-LIGTPSVILEPFK------ : 417
LsH2_-_FUe  : NYEDH-FHYRYDDLQFVGMSIPRLD-AFIKE-KQEHDRVFAGFLLKGFGQSALVSEEICND-----DMSNC-FEGGYFTVLGGSQEMPWRFDRLYKYEITEQLEEHNLRYD--DNYHFHVHVKNIDGTMLPGK-LIPDPTVILVPAQ------ : 419
BgH12_-_FUe : KYETN-FQYRYDNLELAGMSISQLD-AYIKG-KIKHDRVFAGFQLNGLRLSAFVDEEICTV-------RKC-TEGGYFAVLGGAEELPWQFDRLYKYDITQQLKSRALRFD--DDYEIKVHVKSMNGSQLSSS-LIASPNIIFVPSK------ : 417
HpHaD_-_FUe : AYEDH-FEYQYDSLEFVGMNIPALD-AYIKE-RQEHDRVFAGFLLHGLGTSASVNFTICDA------FKKC-FEGGHFTVLGGELEKPWQFDRLYRYPITDVLKENHIHYD--DDYHFHIHIKALNGTELDSK-LIPEPSVLFVAAH------ : 418
CaHaD_-_FUe : AYEEH-FEYQYDSLEFVGMTIPALD-AYIKE-RQEHDRVFAGFLLRGLGTSATVDFTICDA------FKKC-FDGGHFTVLGGEQEKPWQFDRLYRYPITDVLKENHIHYD--DDYHFHIHIKALNGTELDSK-LIPEPSVLFVAAH------ : 418
HpHaN_-_FUe : AYKEH-FHYKYDALEFVGMNIPTLD-AYIKE-RQEHDRVFAGFLLKGIGQSASVKEEICID-----ATGVC-FDGGEFSILGGSAEMPWQFNRLYKYEITDQLESHHVHYD--DEYHLTVHLKSINGTELDSH-LIHEPTVIYVPAK------ : 419
CaHaN_-_FUe : AYKEH-FHYKYDALEFVGMNIPTLD-AYIKE-RQEHDRVFAGFLLKGIGQSATVEEEICSD-----ATGDC-FDGGEFSILGGSAEMPWQFNRLYKYEITDQLESHHVHYD--DEYHLTVHLKSINGTELDSH-LVHEPTVIYVPAK------ : 419
HpHb_-_FUe  : DYERH-FHYRYDALEFVGMNIPTLD-AYIKE-RQEHDRVFAGFLLKGIHQSATVDEDICKG-------NNC-FEGGYFSILGGPAEMEWEFDRLYNYEITDQLKEHHVRYD--DDYNFKIQIIAPDGAILDSH-LLHEPTVSFVPAR------ : 417
CaHb_-_FUe  : YYESH-FHYRYDALEFGGMNIPTLD-AFIKE-RQEHDRVFAGFLLKGIHQSATVDEDICKG-----DTDNC-FPGGYFSILGGPAEAEWEFDRLYNYEITDQLKEHHVRYD--DDYHFKIQIKAPDGTILDSS-LLREPTVSFAPAK------ : 419
HtH1_-_FUe  : DYKK--FGYRYDSLELEGRSISRID-ELIQQ-RQEKDRTFAGFLLKGFGTSASVSLQVCRV------DHTC-KDAGYFTILGGSAEMPWAFDRLYKYDITKTLHDMNLRHE--DTFSIDVTITSYNGTVLSGD-LIQTPSIIFVPGR------ : 420
KLH1_-_FUe  : DYQK--FGYIYDTLEFAGWSIRGID-HIVRN-RQEHSRVFAGFLLEGFGTSATVDFQVCRT------AGDC-EDAGYFTVLGGEKEMPWAFDRLYKYDITETLDKMNLRHD--EIFQIEVTITSYDGTVLDSG-LIPTPSIIYDPAH------ : 419
HtH2_-_FUe  : DYRK--FGYEYDNLEFLGMSVAELD-QYIIE-HQENDRVFAGFLLSGFGGSASVNFQVCRA------DSTC-QDAGYFTVLGGEQAEMAWAFDRLYKYDITETLEKMHLRYD--DDFTISVSLTANNGTVLSSS-LIPTPSVIFQRGH------ : 419
KLH2_-_FUe  : DYHR--FGYEYDSLEFVGMSVSSLH-NYIKQ-QQEADRVFAGFLLKGFGQSASVSFDICRP------DQSC-QEAGYFSVLGGSSEMPWQFDRLYKYDITKTLKDMKLRYD--DTFTIKVHIKDIAGAELDSD-LIPTPSVLLEEGK------ : 419
OdHG_-_FUe  : RYKDN-FHYEYDILDINSMSINQIE-SYIRH-QRDHDRVFAGFLLSGFGSSAYATFEICIE------GGEC-HEGSHFSVLGGSTEMPWAFDRLYKIEITDILSDMNLAFD--SAFTIKTKLVAQNGTELPAS-ILPEATVIRIPPS------ : 420
OdHA_-_FUe  : RYKDN-FHYEYDILDINSMSINQIESSYIRH-QKDHDRVFAGFLLSGFGSSAYATFEICIE------GGEC-HEGSHFAVLGGSTEMPWAFDRLYKIEITDVLSDMHLAFD--SAFTIKTKIVAQNGTELPAS-ILPEATVIRIPPS------ : 421
NpH_-_FUe   : NYKDH-FDYEYDMLEFSGMTIPQLE-DYIKE-SLHHERIFAGFLLHGIGQSANVDEVCDN------QNHC-EDACYFSILGGSLEMPWAFNRLYRYDITHTLETMHLNYD--DNFHINIHIHDMAGNDLDSH-LLQKATILRQPAA------ : 421
AcH1_-_FUf  : DYEH--LGYSYDDLSIGGYDLDGLE-QVIAD-RQSKARVFAGFLLKGVKTSGSVVINICLR------NNVC-NYAGRFNLLGGPTEMAWAFDRLFTYDITSALEQSGINPEDVFDAEALFTLDIKVFDVEGHALPVSSVLPEPTIIYKAAV-- : 419
AcH2_-_FUf  : DHEK--LGYAYDDLTIGGYDLEGLE-DLITA-RRSHARVFAGFLLKGAKTSGSAVIDICRP------NNEC-AYGGRFNVLGGPLEMDWAFNRLFKFDITRALEEAGLHPEDVFDSEALFTLRVKIFNAEGQAVALKSVMPEPTIIFEPAH-- : 419
LsH1_-_FUf  : NHEE--LGYHYDNLNIGGYNLDQLE-QLIAQ-HQSRPRVFAGFLLKGIKTSGSVVLNVCKG-------SQC-TYAGRFNLLGGPSEMPWAFDRLYKKDITWALEAADIKPESVFDADSLFTLDVKVFDVEGHAINVKSVFPTPSIIYDPAK-- : 417
LsH2_-_FUf  : DYEE--LGYAYDTLEIGGYSLEQIE-EIIAD-NKKTARVYAGFLLKGIKTSGSVIIKACKD-------SEC-AFAGRFNLLGGPTEMPWAFDRLFKADITWALRNLGLTADDVTDVDSHFHLEVDITDVKGRSLNRKSVMPEPTIIFRPAE-- : 418
BgH12_-_FUf : NHDL--LGYQYDHLKIGGHSVNDLE-YLIND-KKMKNRILAGFLLKSVNTSVSVKLHVCNC----QECSVC-IYAGGFSLLGGPTERTWAFDHLFKKDITHAVEEAGIEVSDSADFFLDVKVYDRAGKPMKDRSVFPTPSIILVPTKVKCL-- : 420
HpHaD_-_FUf : NHED--LGYAYDNFNIGGYNLDELE-KLIHD-RQIKPRIFAGFLLKGIKTSGSVNLKVCKF-------EEC-TPAGFFNLLGGPLEMPWAFDRLYKKDITWAIAKLGLNPDDIHNTDSGFKLEVQAFNVEGTALPLSQVMPTPSVIYKPAL-- : 418
CaHaD_-_FUf : NHED--LGYAYDNFNIGGYDLDQLE-KLIHD-RQIKPRIFAGFLLKGIKTSGSINLKVCKL-------EEC-TSGGFFNLLGGPLEMPWAFDRLYKKDITWAIANIGLNPDDIHKTDSGFRLEVQAFNVEGTALPLSQVMPTPSIIYKPAF-- : 416
HpHaN_-_FUf : DHEN--LGYTYDHLKVGGYDLGQLE-ELIHD-NQQHARVFAGFLLHGIKTSGSVKIKLCQG-------DKC-TSAGQFNLLGGPLESPWAYNRLYKRDITQYLEDLNLHPEDVFDPILQVHLEVEVRDVQDHLLVAQDVLPQPTIIFDPPH-- : 418
CaHaN_-_FUf : DHEN--LGYTYDNLNVGGYSLDQLE-ELIHD-NQQHARVFAGFLLHGIKTSGSVKIKLCQG-------DKC-TSAGQFNLLGGPLESPWAYNRLYKRDITQYLAALNLHPEDVFDPTLQVHLEVEVRDVQDHVLEAQNVLPQPTIIFDPPH-- : 418
HpHb_-_FUf  : DHEN--LGYGYDNLNIGGYTIEELE-ALIHD-REKEDRVFVGFLLKGIGTSGVVTINICLR------SGNC-TFSGRFSLLGGPLEAPWAYDRLYKKEITHYLADIHIEPK--DVLNASVYLKIEVDDVEGRTLSPKAVFPNPTIIFKAGH-- : 417
CaHb_-_FUf  : DHED--LGYGYDNLNIGGYTIDELE-ELIHE-RQKEDRVFAGFLLKGIHTSGVVTINVCVK------SGNC-TFAGRFSLLGGPLEAPWAYDRLYKREITQYLGEIHIKPR--DILNANVYLKIEVDDVEGRTLSPKAVFPHPTLIFEPGH-- : 417
HtH1_-_FUf  : DYED--LGYNYDNLEISGLNLNEIE-ALIAK-RKSHARVFAGFLLFGLGTSADIHLEICKT------SENC-HDAGVIFILGGSAEMHWAYNRLYKYDITEALQEFDINPEDVFHADEPFFLRLSVVAVNGTV-IPSSHLHQPTIIYEPGE-- : 417
KLH1_-_FUf  : KYEL--LGYRYDNLEIGGMNLHEIE-KEIKD-KQHHVRVFAGFLLHGIRTSADVQFQICKT------SEDC-HHGGQIFVLGGTKEMAWAYNRLFKYDITHALHDAHITPEDVFHPSEPFFIKVSVTAVNGTV-LPASILHAPTIIYEPGL-- : 417
HtH2_-_FUf  : DYET--LYYSYDNLEIGGRNLDQLQ-AEIDR-SRSHDRVFAGFLLRGIGTSADVRFWICRN------ENDC-HRGGIIFILGGAKEMPWSFDRNFKFDITHVLENAGISPEDVFDAEEPFYIKVEIHAVNKTM-IPSSVIPAPTIIYSPGE-- : 416
KLH2_-_FUf  : DFQD--LFYTYDNLEIAGMNVNQLE-AEINR-RKSQTRVFAGFLLHGIGRSADVRFWICKT------ADDC-HASGMIFILGGSKEMHWAYDRNFKYDITQALKAQSIHPEDVFDTDAPFFIKVEVHGVNKTA-LPSSAIPAPTIIYSAGE-- : 417
OdHG_-_FUf  : DDNK--LGYKYDNLEFHGMNIDQLE-NAIHK-QQNKDRVFASFLLFGIKTSADVHLKLCKD-------ETC-EDAGVVFILGGDNEMPWHFDRTYKKDITHVLHQMHIPLEDLYVHGSTILLEVEIETVDGKV-LDSSSLPAPSMIYVPAK-- : 414
OdHA_-_FUf  : DYNK--LGYKYDDLEFHGMNIDQLE-NAIHK-TQNKDRVFASFLLFGIKTSADVHLKLCKD-------ETC-EDAGVVFVLGGDNEMPWHFDRTYKMDITNVLHKMHIPLEDLYVHGSTIHLEVEIESVDGKV-LDSSSLPVPSMIYVPAK-- : 414
NpH_-_FUf   : NYDD--LGYSYDNLLFDGLTITELE-EKIHS-RQSHPRVFAGFLLHAIGTSADVHVSVCHH------GNNC-TSAGLFFILGGKHEMPWKYDRLFRYDITDAVTKAGFSPEDVFNDNLLYKIHTEINAVNGSH-IDSHIMHEPTLIFVPGR-- : 416
AcH1_-_FUg  : DYDR--FNYQYDNLNFHGLTISELN-DLLEK-RKEEDRVFAEFLLAGFGGSGDVVENLCSE-------EEC-AFAGTFAVLGGAIEMPWAFDRLFKYDVTNVFRKLNLRPD--DVYHFEVKIVAVNGTELYPG-LIRPPSVQFVPGVK----- : 403
AcH2_-_FUg  : DYDR--FNYQYDNLNFHGLTISELN-DLLEK-RKEEDRIFAEFLLAGFGGSADVIENLCSE-------TEC-TFAGTFAVLGGAIEMPWAFDRLFKYDVTNVFMKLNLRPD--DVYHFEVEIVAVNGTHLDSG-LIRPPSVQFVPGVK----- : 402
LsH1_-_FUg  : DYDR--LNYQYDNLNFHGLTISQLN-DLLEK-RKEEDRIFAEFLLRGFSASADVIEKLCDE------KGHC-EFAGTFAVLGGLLEMPWAFDRLFKYDVTNVFNKLNLRTD--SDYHFDVEIKAINGTVLDSN-LIRSPSVQFVPGVK----- : 403
LsH2_-_FUg  : DYDR--FNYQYDNLNFHGLTISQLN-DLLEK-RIEEDRIFAEFLLIGFGASADVTFQLCDA------ENHC-AFAGTFAVLGGPLEMPWALDRLFKYDVTNVFNQLHLKPD--SKYHFEVKIVAVNGTELDST-IIRPPTVQFVPGVK----- : 403
HpHaD_-_FUg : NYDR--LNYQYDDLNFHGLSISELN-DVLER-RKEKARIFAEFLLHGIGASADVTFDLCDS------HDHC-EFAGTFAILGGPLEHPWAFDRLFKYDVTDVFSKLHLRPD--SEYHFNIHIVSVNGTELDSH-LIRSPTVQFVPGVK----- : 403
CaHaD_-_FUg : NYDR--LNYQYDNLNFHGLSISELN-DVLER-RKEKARIFAEFLLHGLGASADITFDLCDS------HDHC-EFAGTFAILGGPLEHPWAFDRLFKYDVTDVFSKLHLRPD--SEFHFNIHIVAVNGTELDSH-LIRSPTVQFVPGVK----- : 403
HpHaN_-_FUg : NADS--LHYQYDNLNFHGHSIAELE-EILQH-RQEEDRVFAEFLLHGLKTSADVSFDLCDE------RGHC-EFAGTFAILGGYLEMDWEFDRLFRYDVTNVFNKLHLQPD--SDYHFVDHITAVNGTELDSQ-LIRPPSVHFVPGVK----- : 403
CaHaN_-_FUg : SSDR--LHYQYDNLDFHGHSIVELE-EILQH-RQEEDRVFAEFLLHGLKTSADVTFDLCDE------RNHC-EFAGTFAILGGDLEMDWEFDRLFKYDVTNVFNKLHLQPD--SAYHFVDHITAVNGTELDSN-LIRPPSVHFVPGVK----- : 403
HpHb_-_FUg  : NYEQ--LHYHYDNLNFHGDTISQLV-NVIDE-RKSHDRIFAEFLLHSIGTSADVTFELCDE------HNHC-EFAGTFAILGGPLEMAWSTDRLFRYDVTDVFEKLHLQAD--SEYHFVDHIVAVNGTELDSH-LIKPPSVRFVPGTK----- : 403
CaHb_-_FUg  : NYEQ--LHYQYDNLNFLGHTISELV-DIIEE-RKSHDRIFAEFLLHSIGTSADVTFELCDE------RNRC-AFAGTFAILGGPLEMGWANDRLFKYDVTDVFQKLHLQAD--SEYHFVDHIVAVNGTELDSH-LIHPPSVRFVPGTR----- : 403
HtH1_-_FUg  : EYNR--LSFQYDNLIFHGYSIPELD-RVLEE-RKEEDRIFAAFLLSGIKRSADVVFDICQP------GHEC-VFAGTFAILGGELEMPWSFDRLFRYDITKVMKQLHLRHD--SDFTFRVKIVGTDDHELPSD-SVKAPTIEFEPGV------ : 404
KLH1_-_FUg  : EYSR--FNFQYDNLRFHGMTIKKLE-HELEK-QKEEDRTFAAFLLHGIKKSADVSFDVCNH------DGEC-HFAGTFAILGGEHEMPWSFDRLFRYDITQVLKQMHLEYD--SDFTFHMRIIDTSGKQLPSD-LIKMPTVEHSPGG------ : 404
HtH2_-_FUg  : DYER--FNYQYDTLSFHGKSIPELN-DLLEE-RKREERTFAAFLLRGIGCSADVVFDICRP------NGDC-VFAGTFAVLGGELEMPWSFDRLFRYDITRVMNQLHLQYD--SDFSFRVKLVATNGTELSSD-LLKSPTIEHELGA------ : 404
KLH2_-_FUg  : DYER--LNYQYDTLTFHGHSISELD-AMLQE-RKKEERTFAAFLLHGFGASADVSFDVCTP------DGHC-AFAGTFAVLGGELEMPWSFERLFRYDITKVLKQMNLHYD--SEFHFELKIVGTDGTELPSD-RIKSPTIEHHGGD------ : 403
OdHG_-_FUg  : DYHK--LGYDYDNLNFHGMTIPELE-EHLKE-IQHEDRVFAGFLLRTIGQSADVNFDVCTK------DGEC-TFGGTFCILGGEHEMFWAFDRLFKYDITTSLKHLRLDAH--DDFDIKVTIKGIDGHVLSNK-YLSPPTVFLAPAKTTH--- : 407
OdHA_-_FUg  : DYHK--LGYDYDNLNFHGMTIPELE-EHLKE-IQHEDRVFAGFLLRTIGQSADVNFDVCTK------DGEC-TFGGTFCILGGEHEMFWAFDRLFRYDITTSLKHLHLDAH--DDFDIKVTIKGIDGHVLPNK-YLSPPTVFLAPAKTN---- : 406
NpH_-_FUg   : DYHQ--LGYEYDTLEFHGHNIPQLA-VYLDS-YHEHDRVFAGFLLHGIKRSADVNFDICSL------NGDC-TNAGTFALLGGEHEMPWAFDRLFKYDITKPLQNLRLHST--DDFSINIHIVAINGEELDSH-LIPTPSIIFSPGRGAQH-- : 404
AcH1_-_FUh  : DYAH--LGYEFDNLELNGHDVNDLN-NIINR-LRDQDRVYLAFNNCGKQGSFAFDVHFETA-------ENNVVPLGRFYVLGGEREMPWCFERVFKYDVTEVLQANNVDIH--KPVKIGGQLFRYDGEHVQNF-TTYYATFIERPAGVDYD-- : 412
LsH1_-_FUh  : DSER--LGYKYDKLELNGNSVEEIN-NIIKN-LHHQERIFVGILAFGHQKSLTIHISLIND-------NDEAFDGGNIHILGGEKEMPWAYERIMKLDVTEAIRKGKISTD--HAVKARFTSTDYQGNLNHQD-TDYAIIVERHAEQDY---- : 410
LsH2_-_FUh  : DSER--FGYEFDKLELNGHDVSELN-GIIQR-LRGNTRYFLGFVLYGVRQTVGVEVTLISN--------KKRYPAGNFYVLGGEKEMPWAYERIAKYDVTDVFHKAKLSDD--KPVVGEFYSSLYNGQA-NNN-VTEEVIVVEKPSDVNYD-- : 410
BgH11_-_FUh : DHRR--LGYKFDKLNLNVFSLKDLE-DKIKL-QKSKNRTYAGLMLRGVKNSVTLEVYLQDN------------QVGTVNILGGPNEKPWVFERPYKIDVTDAMKGAQLTTD--KPVKLHLKTGTYDGSSSSEK-DM-EVFIIERPSGSHHD-- : 397
HpHaD_-_FUh : DYTH--FGYEFDKLELNGHDVQGID-DIIHN-LRHGNRVYLGFVLFGQQSSLEYKIDLIDD-------AGQAHTAGSFHLLGGEREMPWAYERLFKYDVTDVAKQFGITTD--HPIKVKVTSTYYNGEPHQEY-TDEIVAVERHADTDYD-- : 411
CaHaD_-_FUh : DYTH--FGYEFDKLELNGHDVQGID-NVIHN-LRHGNRVYLGFVLFGQQSSLEYKIDLIDD-------EGQAHTAGSFHLLGGEREMPWAYERLFKYDVTDVAKKFGITTD--HPIKVKVTSTYYNGEPHQEY-TDEIVAVERHADTDYD-- : 411
HpHaN_-_FUh : DHHL--LGYEYDTTEISEHDAAEVL-EIIRR-RHSETRVYLGGAAYGHGQSFRTAAWVLND-------AGEEFDAGTNFLLGSSKEMPWANEILWKFDITTAVHQAGVSTD--KNIKFHFKIETYNGTLFASE-SAYAIFLVREAYTDY---- : 408
CaHaN_-_FUh : DHHH--LQYDYDTTEISGHDAAEVL-EIIRR-RHSETRVYLGGAAQGYGESYRTETWVLND-------AGEKFSVGTNFLLGSAKEMPWSNEILWKFDITNAVRQAGVSTD--KNIKFHFIAETYNGTLHHDQ-VAYGIFVIREAYTDYLT-- : 410
HpHb_-_FUh  : EHDR--LGYEYDNLRISGYDVEQVN-EILKK-RHAETRIYYSEAAAGYGVSVSYEVWVLDE-------EGHEFPADTGYILGSSKEMPWTYEILFKYDITDAIHKANVSLD--HPVKFKWRTVNYDGTLIEEA-SEYGIVVVREAKTDYL--- : 410
CaHb_-_FUh  : EHDN--LGYEYDNLRISGYDVGQVN-EILKK-RHAETRIYYSEAASGFGVSVSYEVWVLDD-------EGHEIPADTGFVLGSSKEMPWTYEILFKYDITDAFNKANLSLD--HPVKFKWRMVNYDGTFLEEA-SEYGIVVVREANTDYL--- : 410
HtH1_-_FUh  : DHYR--FNYEYDNMRIRGQDIHELE-EVIQE-LRNKDRIFAGFVLSGLRISATVKVFIHSK-----NDTSHEEYAGEFAVLGGEKEMPWAYERMLKLDISDAVHKLHVK-D--EDIRFRVVVTAYNGDVVTTR--LSQPFIVHRPAHVAHD-- : 415
KLH1_-_FUh  : DSHR--FNYKYDNLNLHGHNIEELE-EVIRS-LRLKSRVFAGFVLSGIRTTAVVKVYIKSG------TDSDDEYAGSFVILGGAKEMPWAYERLYRFDITETVHNLNLT-D--DHVKFRFDLKKYDHTELDAS-VLPAPIIVRRPNNAVFD-- : 410
HtH2_-_FUh  : DQHK--FGYHYDNLNVRGHSIQELN-TIIND-LRNTDRIYAGFVLSGIGTSASVKIYLRTD--------DNDEEVGTFTVLGGEREMPWAYERVFKYDITEVADRLKLSYG--DTFNFRLEITSYDGSVVNKS--LPNPFIIYRPANHDYD-- : 414
KLH2_-_FUh  : DSHK--FNYHYDNPDVRGNSIQEIS-AIIHD-LRNQPRVFAGFVLSGIYTSANVKIYLVRE-------GHDDENVGSFVVLGGPKEMPWAYERIFKYDITEVANRLNMHHD--DTFNFRLEVQSYTGEMVTHH--LPEPLIIYRPAKQEYD-- : 412
```
