# Supplementary material for: The evolution of hemocyanin genes in Tectipleura: a multitude of conserved introns in highly diverse gastropods
Source: BMC Ecol Evol. 2021 Mar 4;21:36. doi: 10.1186/s12862-021-01763-3 (PMC7931591; doi:10.1186/s12862-021-01763-3)
Supplement: Supplementary file 3 — Additional file 3. Table S1. Identities of cephalopod hemocyanins. [file 12862_2021_1763_MOESM3_ESM.pdf]

Additional file 3

Additional file 3a

Table S1: Identities of cephalopod hemocyanins. The table shows the identities of amino acid sequences of hemocyanins of the cephalopods *Enteroctopus dofleini* (OdH Type-A+G), *Octopus vulgaris* (OvH Type-A+R), *Octopus bimaculoides* (ObH Type R) and *Nautilus pompilius* (NpH). Accession numbers are included in the very left column. Darker colors indicate higher identities.

	OdH Type-A	OdH Type-G	OvH Type-A	OvH Type-R	ObH Type-R	NpH
OdH Type-A<br>AAU84460.1	x	97.1	89.3	70.0	69.9	58.9
OdH Type-G<br>AAK28276.2	97.1	x	88.9	69.9	69.7	58.8
OvH Type-A<br>XP_029636170.1	89.3	88.9	x	70.8	70.8	60.2
OvH Type-R<br>XP_029652375.1	70.0	69.9	70.8	x	95.8	58.2
ObH Type-R<br>XP_014789836.1;<br>XP_014789967.1	69.9	69.7	70.8	95.8	x	58.2
NpH<br>CAF03590.1	58.9	58.8	60.2	58.2	58.2	x

Additional file 3b

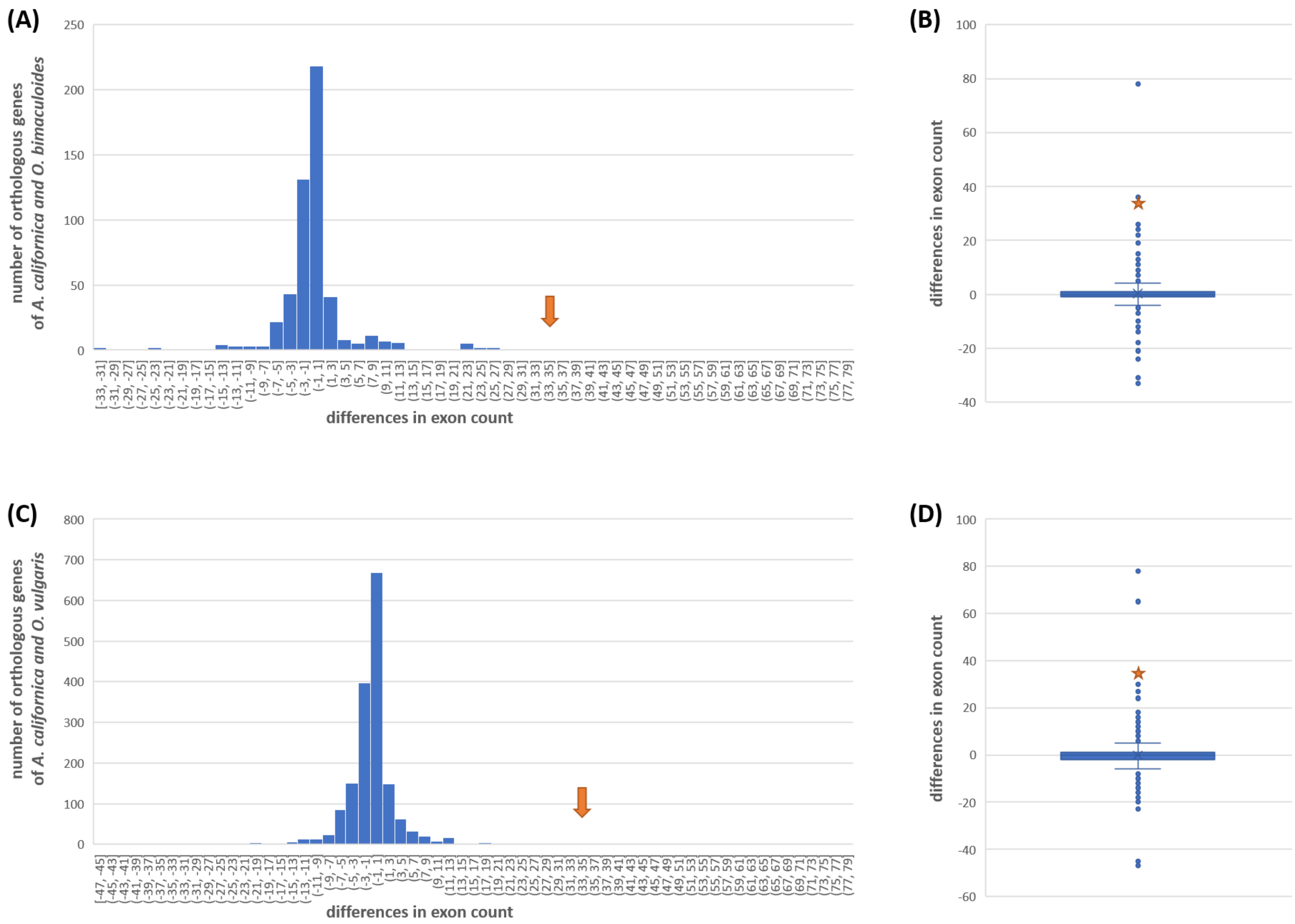

Figure S3: Differences in exon count between orthologous genes. Orthologous genes of *Aplysia californica* and *Octopus bimaculoides* (A+B) / *O. vulgaris* (C+D) were identified via OrthoVenn2 and filtered as described in Materials. The differences between the exon counts of orthologous genes (exon count$_{(Aplysia)}$ – exon count$_{(Octopus)}$) are depicted in histograms (A+C) and box-plots (B+D). For positive values, the genes of *A. californica* encompasses more exons; for negative values, those of *Octopus* species. Hemocyanins are not included in the data but their differences in exon counts for the gene sections coding for FU-a to FU-g are marked with orange arrows in the histograms and orange stars in the box-plots. Histograms show that genes mainly vary in up to 15 exons, many of them even only in up to three. In the box plots, the upper and lower box boundaries represent the 75th and 25th percentiles; the cross inside the median; and the upper and lower error lines the 90th and 10th percentiles. Filled circles represent data falling outside these percentiles. The values for hemocyanin genes (orange stars) clearly fall outside the 10th and 90th percentiles. These diagrams are based on data published at NCBI that resulted from different studies and thus their comparability is limited (see 1.3 in Results / Discussion). Some, for example, are based on assemblies of only genomic NGS data, others in contrast on transcriptomic and genomic data; some resulted from older analysis with a higher error rate, others were obtained by newer techniques or were double checked.

Table S2: Orthologous genes with differences of exon counts > 25. Numbers of the difference in exon counts are color coded: blue (only identified in *O. vulgaris*), green (only identified in *O. bimaculoides*) and black (identified in both *O. species*).

orthologous genes	Differences in exon count
cytoplasmic dynein 2 heavy chain 1	-47; -45; -33
rootletin	-31
dynein heavy chain 3, axonemal	+26
WD repeat-containing and planar cell polarity effector protein fritz h	+27
bloom syndrome protein homolog isoform X1	+27
rho GTPase-activating protein 26 isoform X12	+30
cytoplasmic dynein 1 heavy chain 1	+36
serine/threonine-protein kinase ATR	+65
ubiquitin carboxyl-terminal hydrolase 34	+78
