# Supplementary material for: The evolution of hemocyanin genes in Tectipleura: a multitude of conserved introns in highly diverse gastropods
Source: BMC Ecol Evol. 2021 Mar 4;21:36. doi: 10.1186/s12862-021-01763-3 (PMC7931591; doi:10.1186/s12862-021-01763-3)
Supplement: Supplementary file 4 — Additional file 4: Table S2. Identities of cephalopod hemocyanins. [file 12862_2021_1763_MOESM4_ESM.pdf]

Additional file 4

Table S2: Identities of cephalopod hemocyanins. The table shows the identities of amino acid sequences of hemocyanins of the cephalopods *Enteroctopus dofleini* (OdH Type-A+G), *Octopus vulgaris* (OvH Type-A+R), *Octopus bimaculoides* (ObH Type R) and *Nautilus pompilius* (NpH). Accession numbers are included in the very left column. Darker colors indicate higher identities.

	OdH Type-A	OdH Type-G	OvH Type-A	OvH Type-R	ObH Type-R	NpH
OdH Type-A<br>AAU84460.1	x	97.1	89.3	70.0	69.9	58.9
OdH Type-G<br>AAK28276.2	97.1	x	88.9	69.9	69.7	58.8
OvH Type-A<br>XP_029636170.1	89.3	88.9	x	70.8	70.8	60.2
OvH Type-R<br>XP_029652375.1	70.0	69.9	70.8	x	95.8	58.2
ObH Type-R<br>XP_014789836.1;<br>XP_014789967.1	69.9	69.7	70.8	95.8	x	58.2
NpH<br>CAF03590.1	58.9	58.8	60.2	58.2	58.2	x
