# Supplementary material for: The evolution of hemocyanin genes in Tectipleura: a multitude of conserved introns in highly diverse gastropods
Source: BMC Ecol Evol. 2021 Mar 4;21:36. doi: 10.1186/s12862-021-01763-3 (PMC7931591; doi:10.1186/s12862-021-01763-3)
Supplement: Supplementary file 5 — Additional file 5: Figure S3. Splice site comparison of full hemocyanins. [file 12862_2021_1763_MOESM5_ESM.pdf]

Additional file 5

Figure S3: Splice site comparison of full hemocyanins. Amino acid sequences of functional units included in this multiple sequence alignment comprise all hemocyanins of Tectipleura with a known gene structure as well as one example each of the hemocyanin gene structures known of Lepetellida and Cephalopoda (abbreviations see below). Colors indicate conservation scores: Red/blue: conservation of at least 95%/80% over all FUs; green/yellow: splice site positions of internal/linker introns (amino acids which are coded by the first nucleotides at the beginning of new exons are highlighted). "X" symbolizes not known amino acids. Included hemocyanins: AcH1+2 (*Aplysia californica*), LsH1+2 (*Lymnaea stagnalis*), HpHaD + HpHaN + HpHb (*Helix pomatia* alphaD, alphaN, beta), CaHaD CaHaN CaHb (*Cornu aspersum* alphaD, alphaN, beta); KLH1 (*Megathura crenulata*), OdHG (*Octopus vulgaris*), NpH (*Nautilus pompilius*).

```
                *        20         *        40         *        60         *        80         *       100         *       120         *       140         *       160
AcH1   : -----MVGYLGQALMALLLLALSNAALVRKSVDQLTSEEILNLQKSLREVNDDTSNLGYAAIASYHGYPTQC-KDGDRDIACCLHGSPVFPQWHRLYVVQMEQALKAKGLSIGVPYWDWTRPLNHLPELVSQQVFIDSEGGKARGNVWYQGDINI----D : 150
AcH2   : ------XXXXXXXXXXXXXMGTSNAALVRKSVEHLTPEDILNLQKSLRDVNDDDSQYGYAAIAAYHGYPSQC-KDGDRDVACCIHGMSVFPQWHRLYVVQMEQALKEKGLSIGVPYWDWTRPLNHLPELVSQQVFIDSDGGKARGNVWYQGDINI----D : 136
LsH1   : -----MALLWSFLALALFVLSFGDAALVRKNVDNLSEEDIINLQKTLRDVADDKSAKGYAAIAAYHGYPAQCKDANNRPVACCVHGMPVFPQWHRLYVVQLEQALKEKGLSIGIPYWDWTRPLTKLPALVSQQVFIESDGTKAKKNVWYQGDIEVVENSK : 155
LsH2   : ---------MLLLLQLAALLSFSGAVLIRKNVELLNPEEVLDLQKSLRSVADDKSNKSYNALAAYHGYPAMC-EHQGKPVACCVHGMPVFPQWHRLFVVQFEQALKDKGLSIGVPFWDWTKPLADLPELVRQQVFIDSNGGKAKKNVWYQGSIPL----K : 146
HpHaD  : ------MAPTIVWLAFTFMLVSSDALLVRKDVDHLTPEEVLNLQKALREVNKDTSSKGFAAIAAYHGYPPKC-KHGSKDVACCVHGEPTFPQWHRLYVVQMEQALKEKGLNIGIPYWEWTHQLDHLPELVSQRVFIENDGGKARSNIWYQGQIPT----P : 149
CaHaD  : ------MAPIMVWLWFTCMLVSSNALLVRKDVDHLTPEEVLNLQKALREVNKDTSSKGFAAIAAYHGYPPQC-KHGSKAVACCVHGEPTFPQWHRLYVVQLEQALKEKGLNIGIPYWEWTHQLDHLPDLVSQRVFIETDGGKARSNVWYQGEIPT----P : 149
HpHaN  : ------MSQLWFLISLGLLVWVCQATLLRKNVDHLSQQDVLNLQKALRDVFDDNSSKGFDAIAAYHGYPPQC-QDGDRAVACCLHGDLNFPAWHRLLTVQIEQALHEKGLSIGLPYWDWTPQLYKLPDLVSQRVFLDSAGGKTRKNAWYQGYIHV----G : 149
CaHaN  : ------MSQLWFLISLGLLVWVCQATLIRKNVDHLSQQDVLNLQKALRDVFDDSSSKGFDALAAYHGYPPQC-KDGDRDVACCVHGDLNFPTWHRLYTVQIEQALAEKGLSIGLPYWDWTPQLYKLPDLVSNRVFLDSAGGKTRKNAWYQGNIHV----G : 149
HpHb   : ------MAKLWFALSLALLICLGGCELVRKNVDKLTKDELYDLQQALRDVVADHSEKGYDEIASFHGYPAKC-KHGGHDVACCVHGDPNFPTWHRLLVVQLEQALKDKGLTFGVPYWDWTQELHDLPELVREGVLPDPSGGKNLINPWYEGEIHV----G : 149
CaHb   : ------MAKLWFALSLALLISLGGGDLVRKNVDKLTKDEIYNLQQALRDVVADHSEKGYDEIASYHGYPAKC-KHGDKNVACCVHGDPNFPIWHRLLVVQLEQALKDKGLTFGVPYWDWTQELHDLPELVREGVLPDPAGGKNLNNPWYEGEVHV----G : 149
KLH1   : --------MLSVRLLIVVLALANAENLVRKSVEHLTQEETLDLQAALRELQMDSSSIGFQKIAAAHGAPASC-VHKDTSIACCIHGMPTFPHWHRAYVVHMERALQTKRRTSGLPYWDWTEPITQLPSLAADPVYIDSQGGKAHTNYWYRGNI------G : 145
OdHG   : ---MKILCLFAFVFAFWLSGQSAEGNLIRKDVDALSEDEVLNLQVALRAMQDDETPTGYQAIAAYHGEPADCKAPDGSTVVCCLHGMPTFPLWHRLYTVQFEQTMVGHGSKLGVPYWDWTQPLNHLPELVSHPLFMDPTAHKAKKNVFYSGNI------A : 151
NpH    : MATHWHSLLLFSLQLLVFTYATSDPTNIRKNVADLTHDVALNLQIALRTMQDDNSPIGFQAVAAYHGEPASCIDSHENLVVCCLHGMPTFPHWHRLYTTHLEQSLLALGTTLGLPYWDWTQPMTHLPNIVQHPLFIDPNGGKAKKNVFYSGTI------G : 154

                *       180         *       200         *       220         *       240         *       260         *       280         *       300         *       320
AcH1   : GQVVHTARAVDDRLFQQVAPGENTQLFEMVLNALEYENYCQFEVQFEVAHNTIHYLVGGRHKYSMSHLEYTSYDPIFFLHHSNVDRILAVKLALLGPGAPQKGG-----VEFCDLKNSMTPMAPFSWDSNPIALTRDHSLPPQTLAHS-TFGYSYDDLSL : 304
AcH2   : GHVVHTARAVDERLFQQVEAGERTDLFEQVLNALEYENYCQFEVQFEVAHNTIHYLVGGRHTYSMSHLEYTSYDPIFFLHHSNVDRIYAVYEELQKLRGNRRGNACVGECELCDIAAFQKELVPFNWDSNPFPLTKRHNKAYEVIDHS-LFGYRYDDLTL : 295
LsH1   : TVIRHTARALDDRLFQKVEPGQNTDLFEQVLNALMYPNYCQFEVQFEIAHNTIHYLVGGRNKYSMSHLEYTSYDPIFFLHHSNVDKIYSIYEALQKEKGYTPGPGCTGKCELCDLKHFDEPLAPFSWETNPFAVTKQFNLPREAVDGS-IYGYKYDNLTL : 314
LsH2   : TGPRFTARAVDDRLFQKVEAGEHTDLFEQVLNALEYPNYCQFEVQFEVAHNTIHYLVGGRNRYSMSHLEYTSYDPIFFLHHSNVDRIYAVYEDLQRLRGYTPGPGCKGECETCDIKGFQKPLEPFNRASNPWPRTRNHATGFESLDHV-LFGYQYEDRTL : 305
HpHaD  : EGVKNTARAVDPRLFQQVETGQYTDLFEHVLNALEYPNYCQFEVQFEVAHNTIHYLVGGRHTYSVSHLEYTSYDPIFFLHHSNVDKIYTIYETIQRSRGYTPGPGCTGECELCDIVGFRTPLEPFSRDSNPFALTRVHSHPYEATEHT-LFGYKYDNLTL : 308
CaHaD  : EGVKKTARAVDPRLFQQVVAGQYTDLFEHVLNALEYPNYCQFEVQFEVAHNTIHYLVGGRHTYSVSHLEYTSYDPIFFLHHSNVDKIYTIYETIQRARGYTPGPGCTGECELCDIVGFRTPLEPFSRDSNPFALTKTHSHPYEATENT-LFGYKYDNLTL : 308
HpHaN  : KKTYKTARAVDKRLYQNVTGEEHTDLFEQVLHAFEY-GYCQFEVQFEVAHNTIHSLVGGRYPYSVSSLDYTGYDPLFYLHHSNVDRLYAIYQEVQKLRGVQSGS---ANSPLCDVKSYYRPLEPFRRKSNPFSLTRTYNSADKAKDYS-VFGYEYESLEL : 304
CaHaN  : AKTYKTARAVDKRLYQNVSGDEHTDLFEHVLHAFEYSSYCQFEVQFEVAHSTIHSLVGGRFPYSVSSLDYTAYDPLFYLHHSNVDRLFAIYQEVQRIRGIESGS---ANSPICDVKTFYRPLEPFSRTSNPFSLTKTYNSPDKAKDYS-VFGYEYESLEL : 305
HpHb   : DKTYHTSRAIDERLYQHVAPGQHTDLFEHVLDAFEYTDFCQFEVQFEVSHNYIHSLVGGRSQYSLSSLEFTVYDPIFFLHHSNVERLFQIYTEVQKYREAKGEG---GQKVICDIKGYFAPLEPFSRASNPFPVTRENSSPEKALTNSAAFGYSYDDLTL : 306
CaHb   : DQTYHTTRAIDERLYQHVAPGQHTDLFEQVLDAFEYTSFCQFEVQFEVSHNYIHSLVGGRSQYSLSSLEYTVYDPIFFLHHSNVERLFQIYTAVQKHREVENEA---GKKEICDLKGFFAPLEPFNRDSNPFPVTRENSSPDKSQDSKAAFGYSYDDLTL : 306
KLH1   : FLDKKTNRAADDRLFEKVKPGQHTHLMESVLDPLEQDEFCKFEIQFELPHNAIHYLVGGKHDYSMANLEYTAYDPIFFLHHSNVDRIFAIWQRLQELRNKDPKA------MDCAQELLHQKMEPFSWEDNDIPLTNEHSTPADLFDYR-QLHYDYDTLNL : 298
OdHG   : FEKKTTARAVDTRLFQASK-GGKNFLLEGVLSALEQDDYCHFEVQFEVAHNPIHYLVGGRFTHSMSSLEYTSYDPLFFLHHSNVERLFTIWQALQKHRGI-EGN------ANCGLNMFHKPMEPFGRDTNPISLTKEHAKAVDVFNYN-ELGYDYDDLHL : 302
NpH    : FKKMQTARAVDERLFSQPEPGHHTFLFEGILDALEQTDYCQFEVQFEIIHNAIHYLVGGQFPHSMSSLEFTSYDPLFFLHHSNVDRLFSIWQALQRHRGLPSGH------SNCAKELFTVPMKPFNEDSNPIQLTHDHALPSQLFDHT-KLLYQYDDLTL : 307

                *       340         *       360         *       380         *       400         *       420         *       440         *       460         *       480
AcH1   : NGYSVEEIRALLKERQTYDRAFANFRLFGIGSSANVRVEVCVP-SSDKLTGDYCEFAGDFFILGGAIEMAWAFIWPYYFEITKTVQNLGVALDGNYNVQARIFSVNGTELPSNLIPSPSVSFRPAVGKTDPPMAHGESEHGHEVHDGVSIRKDIDTLTRE : 463
AcH2   : NGMDLETIKALLKEKQTHDRAFASFRLAGIGGSANVRVEVCVP-GANDVTGDYCEFAGDFFILGGPTEMAWTFTWPYFFDITKTVQHMGIDLEGDYHVQARIFSVNGTELPSDLIPSPSVSFRPALGKTDPPISHHDDAHEHEFQEGVAVRKDISRLTRE : 454
LsH1   : NGYDLNGIKRLLKEKWSHAASYAVFRLSGISTSANVRVQVCVP-SEDEVTGTYCEFAGDFFVLGGPLEMPWAFSRPYYFEVTKTVEKLGLALDGNYRIEAELFSVNGTQLPSSLLPHPFVSYRPAKGTVDTPIAHNEVNHEHEYHEGISVRKDIDRLTNE : 473
LsH2   : NGHDVDEIFRILKERQKHDRAFAAFKLAGIGTSADVKVKVCLP-ALDAVTEDHCEDAGHFFILGGPLEMPWRFSKPYYFDITKTVASLGLPLDGNYHVEAEIYSVNGTLLPPGLLPAPYVSYRPAKGKIDPVIEISGPDHD---HEKVAVRKNVDRLTDV : 461
HpHaD  : NGLDVDKIKSIIEKRQETDRAFASFRLHGIGVSADVRVKVCVS-DSHHLAGDYCEFAGNFFILGGPLEMPWTFNRPYYFEITKTVKQMNISLTSDYHVEVDVYSVNGTQLPSDILPHPSVSFRPAVGTRDPVIVQKLEH-----KEEVSIRKDVDHLTRE : 462
CaHaD  : NGLDVNKIKGIIEKRQAADRAFASFRLHGIGVSADVRVKVCVS-DSHHLAADYCEFAGNFFILGGPLEMPWTFNRPYYFEITKTVEAMNISFTSDYHVEIDVYSVNGTQLPSDILPHPSVSFRPGKGKSDPEIVYELEH-----KEEVSIRKDVDHLTRE : 462
HpHaN  : NGLSPAQIVDLVKKRQSHDRAFAVFSLHNIGVSADVKVRVCLDIDDEHEEDDRCLHAGDFFVLGGATEMDWAFPRPFFFEITHTVERLHLPLDGNYHVEADIVSVNGTQLPSSILPHPYVSFRPAAGSHDKPIEHNEPEHEHEFHENVAVRKNVDRLTRE : 464
CaHaN  : NGLSPAQIVELVKQRQSHDRAFAAFSLHNIGVSADIKVRVCLDIDDEHEEDDRCLHAGDFFVLGGATEMDWTFPRPFFFEITHTVEQLHLPLQGNYHVEADIVAVNGTQLPSSLLVHPYVSFRPAAGSHDEPIDHNLPGHEHEFHENVVVRKNVDRLTIE : 465
HpHb   : NGLSPKAIADLIKEKQSHDRAFADFELHNIGASADVRVKVCVDLDGLHHTKPHCEHAGDFFILGGPLEMAWSFGHPFHFEITKTVQKLGLPLDGNYHIEVDIFHINGTELPSNVLPRAGVDFRPAVGKSDPPIGHNEEGHEHEFHEGVSVRKNVDRLTVE : 466
CaHb   : NGLSPKAIADLIKEKQSHDRVFADFELHNIGASADVRVKVCVDLDGLHHTKPHCEHAGDFFILGGPLEMAWSFGNPFHFEITKTVQKLGVPLDGNYHIEVDIFHINGTELPSDVLPRAGVDYRPAVGKTDPPIGHNEEGHEHEFHDGVSVRKNVDRLTVE : 466
KLH1   : NGMTPEELKTYLDERSSRARAFASFRLKGFGGSANVFVYVCIP-DDNDRNDDHCEKAGDFFVLGGPSEMKWQFYRPYLFDLSDTVHKMGMKLDGHYTVKAELFSVNGTALPDDLLPHPVVVHHPEKGFTDPPVKHHQ-------SANLLVRKNINDLTRE : 450
OdHG   : NGMDIPELDTMLKERQQHPRSFANFRLGGIKTSANVRVAVCIPSEDKRHSDNCNNHVGSFFILGGVHEMTWDFGYPFLFEITDVVKSLGIPLDGNYYVHADVTAINGTLLPDGTIPRPTVSYIPAHNFKDADMVVVD-------KTGLNVRKDLQSLTTE : 455
NpH    : NGLNITQVQELIEKRRSHARAFASFRLSGIHTSANVRVHVCVRIDDDKSHDDCDHVAGNFFILGGAREMEWHYHYPYRFDITSTVQELGIALDSNYYVDIDLIAINGTLLSSSLIPNPTVDYQPAPGSSDPPMRQID-------REKLVVRKDLRRLHDT : 460

                *       500         *       520         *       540         *       560         *       580         *       600         *       620         *       640
AcH1   : EVNDLRQALTKFQNDASVDGYQATAEFHGDPGKCPYPNAKNRLACCIHGMPTFPHWHRLFVVQVEDALRRRGAQIGIPYWDWTKPGTQIPALAAEETYVDPENGETVRNPFFGADVAFLNSGAKTSRDV-SDSLNDVPEWGDHTNLFDQYLYALEQEDFC : 622
AcH2   : EVYELRQAMQKFQNDASVDGYQATAEFHGDPGKCPYPSAKNRLACCIHGMPTFPHWHRLFVVQVEDALRRRGAHIGMPYWDWTKPKTTIPALAADESYVDPTSGESVHNPFYNAQIAFLGNGVVTSRDV-LEGLTQTPTFGDHTDLFDAYLLALEQDNFC : 613
LsH1   : EIYELREALQKFQNDKSVDGYQAIAEFHGDPGKCPNPNSKNRKACCIHGMPTFPHWHRLLVVQVEDALRRRGSNIGVPYWDWTKPRTSVPKLAADETYTDPTTNEVKRNPFHDAPIAFLGDSTKTTRDV-SSSLSDSPKWGDHTELFDAFLLALEQDNFC : 632
LsH2   : EIYELRQALKKFQNDKSVDGYQAIAEFHGDPGKCPNPTAKDRMACCVHGMPTFPHWHRLLVVQVEDALRRRGSPIGIPYWDWTRPNTKIPDLANDVKYIDPKTKDELDNPFHDAEISFLDNNVHTSRDV-QKDLSQTPLWGDHTELFDAFLLALEQDNFC : 620
HpHaD  : EILELREALEKFQSDHSVDGYQAIAEFHGDPGKCPFPTAKDRFACCIHGMPNFPHWHRLLVVQVEDALRRRGAHTGIPYWDWTKPNTHIPALAADETYVNPHDNAEHVNPFHHAVIGFLGGDAKTSRDP-LPELTQTPKWGDHTELFDVFLLALEQDNFC : 621
CaHaD  : EVLELREALEKFQSDHSVDGYQAIAEFHGDPGKCPFPTAKDRFACCIHGMPNFPHWHRLLVVQVEDALRRRGAHTGIPYWDWTKPNTKIPVLAADETYINPHDNAEHVNPFHHAVIGFLGGDAKTSRDP-LPELTQTPKWGDHTELFDVFLLALEQDNFC : 621
HpHaN  : EVVQLREALQEFLNDKSVEGYQAIAEFHGDPGKCPDPTAKDRHACCIHGLPTFPHWHRLIVTQVEDSLRRRGAPLGVPYWDFTRPDTHVPLLASEEIYFNPHTGGNLHNPFHDVQIAFLGNDVHTERDI-SPSLAQTPAWGEHTELFNAFLLALEQDYFC : 623
CaHaN  : EVVQLREALQEFQNDKSVEGYQAIAEFHGDPGKCPNPTAKDRHACCIHGLPTFPHWHRLIVTQVEDSLRRRGAPLGLPYWDFTRPDTHVPQLANDETYFNPHIGQNVHNPFHDVEIAFLGNDVRTERDI-SESLAQTPAWGEHTELFNAFLLALEQDNFC : 624
HpHb   : EVAEIREALEKFQNDRSVDGYQAIAEFHGDPGKCPSPTARDRLACCVHGMPTFPHWHRLLVVQVEDALRRRGSPIGLPYWDFTRPGVHIPDLANEETYIDPHTGAKHHNPFHDAEIAFLGHDVHTSRAV-LPGLSQTPDWGDHTELYDAFLLALEQDDFC : 625
CaHb   : EVAQIREALEKFQNDRSVDGYQAIAEFHGDPGKCPSPTARDRLACCVHGMPTFPHWHRLLVVQVEDALRRRGSPIGLPYWDFTRPGTSIPDLASEENYIDPHTGAKRHNPFHDAEIAFLGHDVHTSRDV-LPGLSQVPDWGDHTELYDAFLLALEQEDFC : 625
KLH1   : EVLNLREAFHKFQEDRSVDGYQATAEYHGLPARCPRPDAKDRYACCVHGMPIFPHWHRLFVTQVEDALVGRGATIGIPYWDWTEPMTHIPGLAGNKTYVDSH-GASHTNPFHSSVIAFEENAPHTKRQI-DQRLFKPATFGHHTDLFNQILYAFEQEDYC : 608
OdHG   : EEYELRVAMERFMDDKSIDGYQALAEFHGLPAKCPEPDAINRVACCVHGMSTFPHWHRLVVMQFEDALLARGSPIGVPYWDWTTPSSSLPHLVAVETYEDPYTKEVKPNPFYHAKIEFLHNDVFTARNV-DSRLFEKPTKGHHGYLHDGMLLAFEQEDFC : 614
NpH    : EEFGLRQAMARFMKDESIDGFQALAEYHGLPARCLTPEG-NRIACCIHGMATFPHWHRLFVLQVEDALIRRGSPIGIPYWDWARPMISLPHLIADETYINPYSNKSVPNPFHHGSIDFLHDHITTQRDIKQETLFEQPAFGDHTYLFDGLMYALEQDDFC : 619
```

```
                      *       660         *       680         *       700         *       720         *       740         *       760         *       780         *       800
AcH1   : NFEVQFEVAHNLIHALVGGNTQFGLSSLSYSAFDPIFFLHHSNVDRIWAIWTALQQYRGKPYKAHCAQSYVNQPLKPFAFSSPLNNNEKTFSHSVPTNVYDYAEELGYDYDNLQFGGMGIAELDQYIKSTQKSKDRVFYGILLHGIKKAALATVYVKKPG :  782
AcH2   : DFEVQFEVAHNMIHALVGGNAPFGLASLSYSAYDPIFFLHHSNVDRIWAIWTALQQHRGKPYKAHCAQSYVHQPMKPFAFSSPLNNNEKTFSHSVPTNVYDYEKELGYAYDSLHYGGMGIAELDSYINENLKGKSRTFVGILLHGIKKSAIATVFVNAPG :  773
LsH1   : DFEVQFEVAHNLIHALVGGKEQYSLATLSYSAFDPIFYLHHSNVDRIWAIWTALQELRGKPYKAHCAQSYTYEPLKPFAFSSPYNNDQKTFTHAVPRNVYDYETELNYAYDSLEFGGLTIKQLNDYINNERKTKNRVFAGVLLWGIKRSALATLYVNDVE :  792
LsH2   : DFEVQFEIAHNLIHGLVGGNSKYGLSSLSYSAFDPIFYIHHSNVDRIWAIWTALQQHRGKPYKAHCAQSYTYEPLKPFAFSSPYNNNEKTFSHSTPRNVYDYEAELGYAYDSLEFGGMAIPELDDFINSNLKGRSRVFVGILLSGIKKSGIAKIKVITMR :  780
HpHaD  : DFEVQFEIAHNLIHAYVGGNSKYGLSSLSYSAFDPIFYLHHSNIDRIWAIWTALQQHRGKPYKAHCAQSYVYTPLKPFAFHTPYNNNEKTFAHSTPTDVYDYEEKLQYGYDNLQFGGLGIPELENYINEHLKSKSRTFVGIHLHGIKTSGLATIHVSASG :  781
CaHaD  : DFEVQFEIAHNLIHGYVGGNSQYGLSSLSYSAFDPIFYLHHSNIDRIWAIWTALQQQRGKPYKAHCAQSYVYTPLKPFAFHTPYNNNEKTFAHSTPTEVYNYEKEFQYGYDNLEFGGFDIPELENYINEHLKSKPRTFVGVHLHGIKTSGLATIHVSASG :  781
HpHaN  : KFAIQFEVAHNLIHGLVGGNTPHGLSTLSYSAFDPIFYIYHSNIDRIWAIWTALQEHRGKPYKAHCAQSYVHTPLKPFAFKPPYNNDAKTYAHSTATNIYDYEKELAYTYDSLEFGGMSVPELDNYINTNIKNKNRIFVGIQLHGIKTSGLAHIFIEAPG :  783
CaHaN  : KFAVQFEVAHNLIHGLVGGNTSHGLSTLSYSAFDPIFYIYHSNIDRIWAIWTALQQLRGKPYKAHCAQSYVHTPLKPFAFKPPYNNDAKTFAHSTATNIYDYEKELAYTFDSLEFGGMSVPELDNYINTNIKNKNRVFVGIQLHGIQTSGLAHIYIEAPG :  784
HpHb   : DFEVQFEVAHNLIHGLVGGNSAHGLSSLSYSAFDPIFYIYHSSIDRIWAIWTALQQHRHKPYKAHCAQSHVHTPLKPFAFSSPYNNDEKTYSHSTPTNVYDYEQELDYAYDSLEFGGLSIAELDDYIDNQIKTKERVFVSIQLHGIKKSAWATIYVTPPG :  785
CaHb   : DFEVQFEIAHNLIHGLVGGNSAYGLSSLSYSAFDPIFYVYHSSIDRIWAIWTALQQRRNKPYRAHCAQSHVHTPLKPFAFSSPYNNDEKTYSHSIPTNVYDYEAELGYAYDTLEFGGLSVAELDDYINNQIKTKDRVFVGIQLHGIKKSAWATIYVTPPG :  785
KLH1   : DFEVQFEITHNTIHAWTGGSEHFSMSSLHYTAFDPLFYFHHSNVDRLWAVWQALQMRRHKPYRAHCAISLEHMHLKPFAFSSPLNNNEKTHANAMPNKIYDYENVLHYTYEDLTFGGISLENIEKMIHEN-QQEDRIYAGFLLAGIRTSANVDIFIKTTD :  767
OdHG   : DFEVQFEVTHNAIHAWVGGNEPYSMSSLHYTSFDPLFWLHHSQVDRLWAVWQALQIHRGKPYKPYCALSEVHRPLKPFAFEPPLNNNKHTHSHSVPTHVYDYQSDLHYTYDTLFFGGMSVRELQRHIEED-KAKDRVFVGFLLMGIKTSANVVINVESAG :  773
NpH    : DFEIQFEIVHNGIHAWTGGQQTYSMSSLHFTSFDPLFFLHHSNVDRIWAIWQALQIYRGKHYLAHCANSEVHSPMRPFSFPSPRNPNPRTFTHATPTDIYDYEAELGYTFDTLDFGGRSIPDLQNEIEEH-QRHDRTFAGFLFWGIHTSTNGKVFVDVIG :  778

                      *       820         *       840         *       860         *       880         *       900         *       920         *       940         *       960
AcH1   : VDTEYNAGQFALLGGPSEMEWRFDRNYRFEITKALEELGINFLSPFDVRLEMNEFDGTPIDVSQFPKSQLIFKRRDILPEAQIDEV-TVRKNVDSLNAEEVLALRRALANLKEDSSIGGYQTLGRYHGTPLWCPAPDAEKKVACCLHGMPVFPHWHRLLT :  941
AcH2   : KE-KYNAGQGALLGGPSEMAWGFDRLYRHDITDAMSALGLHWSSPFEVSIEMHEFDGTPVDVSEFPRVQLIHKNKQDDAASSTDDI-IVRKNVNDLNAEEVVELRRALANLQEDKSAGGFQDLGRYHGTPKWCPAPDAEKKVACCVHGMPVFPHWHRLLA :  931
LsH1   : A------GRFALLGGPSEMEWRFDRLYRHEITDTLEELNLHWAQPYTVTLKLTDFEGHDIPINPFPKPQIIFKAKEEPKDVSADTI-KIRKNVATLTEAEVVDLRQALANLQNDQSAGGYQDLGRFHGTPNWCPTPDAEVKKSCCLHGMPTFPHWHRLWT :  945
LsH2   : NE-EYEAGRFALLGGPSEMPWSLDRVYRHDITTALEKMGLFWAQPYEVKLEMTEFDGTPIDVRLFPRIQLIYRAPEHPKAAAEHDV-HVRKNVDQLTEEEVHDLRQALSNLQDDKSNGGYQDLGRFHGTPKWCPSPEAEKKVSCCLHGMPTFPHWHRLHA :  938
HpHaD  : K--DYVAGHFAILGGSSEMDWDYDRSYRHDISHALEELGVNWAQPFDVTIEMHTFDNKPIDVSQFPRINIIHKEQEQAHDISHITK-ITRKNVNHLTEEEILNLRHSLAFLEEDRSVGGYQTLGRFHGTPNWCPYPSAEKKVACCPHGGPTFPHWHRLLT :  938
CaHaD  : K--DYVAGHFAILGGPSEMDWEYDRPYRHDISHALEELGVNWAQPFDVTIEMHTFDNKPIDVSQFPRINIIHKEGEQLDETSQVTK-ITRKNVNHLTKEEVLNLRQALTFLEEDRSVGGYQTLGRFHGTPNWCPYPSAEKKVACCPHGGPTFPHWHRLLT :  938
HpHaN  : KE-KLEAGRFAILGGPTEMPWRYDNLYKHEITNALEKLDLHWAQPYNVTIEIAQFDGTPIDAKQFGHLEILYLPGKQTKKPADVEV-HVRKEVSQLTEEEILDLRHALSNLEEDKSIGGYQTLGRYHGAYKWCPSPSAEVKKVCCPHGMSIFPHWHRLLV :  941
CaHaN  : KD-KFEAGRFAILGGPTEMPWRYDRLYKHEITNALETLQLHWAQPYNVNIEIARFDGTPIDAHQFGHVEVLYLPGKQTKKSADVEV-HVRKEVSQLTGEEILDLRHALANLEVDKSIGGYQTLGRYHGASKWCPSPSAEVKKVCCPHGMAIFPHWHRLLV :  942
HpHb   : GE-KYTAGHFALLGGPSEMPWSFDRVYYHDISHALDALNLQWKQPFDVTLELKEFNGTPIDVSQFPKLHLYYKPGQAGHAVKKTES-RLRKEVDHLTAEETLELRHALTALEEDTSLGGYQTLGRYHGATLWCPSPDAQKKLACCLHGMPTFPHWHRLLT :  943
CaHb   : GE-KYTAGHFGLLGGPSEMPWSFDREYHHDISHALDALNLQWSQPFDVTLELKEFDGTPIDISLFPKVHINYQPAEAAHAARKSES-RLRKEVDHLTAEEILDLRHALTALEEDTSLRGYQTLGRYHGATLWCLSPDAQKKVACCLHGMPTFLQWHRLLT :  943
KLH1   : SV-QHKAGTFAVLGGSKEMKWGFDRVFKFDITHVLKDLDLTADGDFEVTVDITEVDGTKLASSLIPHASVIREHARVKFDKVPRSR-LIRKNVDRLSPEEMNELRKALALLKEDKSAGGFQQLGAFHGEPKWCPSPEASKKFACCVHGMSVFPHWHRLLT :  925
OdHG   : N--TYMAGTITILGGSKEMEWRFDRLYKYEITDALAELGVDMHAEYSINLQINDINGTALPPTSIPDPIVIFSPGKKESGVVFDELYRSRRDVSSLTDADMNALRKALQAYEDDKDASGYQQVAAFHGSTKWCPSPDAEVKYACCHHGMATFPHWHRLLT :  931
NpH    : QA-TYNAGTIAILGGTKEMTWRFDRLYHFEISEALKALGKTADDEFSLHVKIHDVNGSRLSNDIIPAPNIIFVPAHVKSMNISHKG-HTRRNINSLTQESLYELRQALTSFMADTENTGFQRYAAFHGFPEWCPSPDAEHKFACCVHGMPTFVHWHRLLT :  936

                      *       980         *      1000         *      1020         *      1040         *      1060         *      1080         *      1100         *      1120
AcH1   : VQAENALRRHGFSGGLPYWDWTRPMTQLPDLVDSAKYSDPKSGQELDNPFYSGHIDDANADTVRSVRDDLFQQPPFGGYTDIAKQVLLALEQDDFCTFEVQYEIAHNFIHALVGGSEQFSMASLQYTAFDPIFFLHHSNTDRIWAIWQALQKYRGKPYNT : 1101
AcH2   : VQTENALRRHGYTGALPYWDWTRPMTSLPSLVDDALYVDPSNGKETRNPFYSGHIDDVDQYTVRSVRDDLYQQPNFGDYTDIAKQVLLALEQDDFCTFEVQYEIAHNFIHALVGGSEQFSMASLQYTAFDPIFFLHHSNTDRLWAIWQALQKYRGKPYNT : 1091
LsH1   : VQAENALRRHGYVGAIPYWDWTQPVKSLPDLVTSEKYRDPSSNNEIKNPFYSAHITDADQDTVRSPRDDLFEQPKFGQNTAIAKQVLLALEQDNFCDFEVQFEIAHNFIHALVGGNEQFSMASLLYTAFDPLFYLHHANTDRLWAIWQALQKYRNKPYNT : 1105
LsH2   : VQAENALRRHGYHGGLPYWDWSRPLKSLPALVSSPTYLDPSNNEQVKNPFYSAHIDDVDHDTVRSVRSDLFQQPGYGEYTDIAEQILLALEQDNFCDFEVQFEIAHNFIHALVGDKEPYSMASLMYTAFDPLFYLHHSNTDRIWAIWQALQNYRGKPYNT : 1098
HpHaD  : VQAENALRKHGYKGALPYWDWTQPATSLPAIVVPETYIDPSNNQETHNPFHDAHIDDVNQNTVRSVRSDLYQQPAFGEYTDIAKQVLYALEQDNYCDFEVQYEIAHNFIHALVGGSEVYSMASLLYTAFDPLFYLHHSNTDRIWAIWQALQAYRGKPENA : 1098
CaHaD  : VQAENALRKHGYKGALPYWDWTQPATSLPEIVVPETYIDPSNNVETHNPFHDAHIDDVNQNTVRSVRDDLYQQPAFGEYTDIAKQVLYALEQDNYCDFEVQYEIAHNFIHALVGGSEVYSMASLLYTAFDPLFYLHHSNTDRIWAIWQALQTYRGKAENA : 1098
HpHaN  : VQFENALSSHGYHGAVPYWDWTLPLDSLPALVTAQTYPDPASNKDVANPFYSAHIDDVNQDTERSPRPELFQKPKYGEYTSIAKQVLLAFEQEDFCDFEVQFEIAHNFIHTLVGGSKPYSMASLAYTAYDPIFYLHHSNTDRLWAIWQALQKNRGKPHNS : 1101
CaHaN  : VQFENALTNHGYHGAVPYWDWTQPLDSLPALVTAETYTDPGSNKDVANPFYSAHIDDANQDTERSPKPELFLKPKYGDYTAIAKQVLLAFEQEDFCDFEVQFEIAHNFIHTLVGGSKPYSMASLAYTAYDPLFFLHHSNTDRLWAIWQALQKNRGKSHNS : 1102
HpHb   : VQAENALRKHGYHGGLPYWDWTLPLTSLPEIVKSPEYVDPSNGQTVKNPFYSGHIDDASADTVRSVRPDLFVDPGFGHYTDIAKQVLLAFEQDNFCDFEVQFEIAHNFIHALVGGSEVYSLASLAYTAYDPLFYLHHSNTDRLWAVWQALQQYRGKPFNS : 1103
CaHb   : VQAENALRNHGYHGGLPYWDWTQPLTALPAIVTSPTYVDPSNGNTVKNPFYSGHIDDASADTVRSVRPDLFKNPGFGHYTDIAKQVLLAFEQDNFCDFEVQFEIAHNFIHALVGGSELYSLASLAFTAYDPLFYLHHSNTDRLWAVWQALQQHRGKPFNS : 1103
KLH1   : VQSENALRRHGYDGALPYWDWTSPLNHLPELADHEKYVDPEDGVEKHNPWFDGHIDTVDKTTTRSVQNKLFEQPEFGHYTSIAKQVLLALEQDNFCDFEIQYEIAHNYIHALVGGAQPYGMASLRYTAFDPLFYLHHSNTDRIWAIWQALQKYRGKPYNV : 1085
OdHG   : VNFENGLRHNGYQNGIPYWDWTRPLSELPTLVKDETYAD-ENGETHPNPFFSGVIDEIGEHTTRSPNPTLFLKPPFGHFTPLGDEVMYALEQEDFCSFEVQFEIAHNHIHALVGGTEPYSMSSLEYTTFDPIFILHHSNVDRIWAIWQALQKFRGHRYNS : 1090
NpH    : IQFENGIRNHGYQCGLPYWDWTEAITHLPESLTAAKYTDPHSNEMHHNPLHNGHIDSVNADTMRNINEKLFEQPEFGHFTLLANQVILALEQENFCDFSVQFEISHNEIHALVGGTEPYSMASLTYTAFDPLFMLHHSNVDRIWAIWQSLQQYRGHPHKS : 1096

                      *      1140         *      1160         *      1180         *      1200         *      1220         *      1240         *      1260         *      1280
AcH1   : ANCAIGQLRNPLSPFSLTSDINPDPVTREHSVPFQVFDYQTNFHYEYDALEFNGLSVPQLARVLEQNKAGDRVFAGFMLHGIQQSALVKFYICKSDDDCNNYAGEFYVLGDVNEMAWSYDRLYKYEITDALAALGLRYNDRYSVRYEVLNLQGEDIG-QP : 1260
AcH2   : ANCAIGQLRNPLSPFSLTSDINPDPVTREHSIPFQAFDYRSSFHYDYDNLEFNGLTVPQLARVLEQNKAEDRVFAGFMLHGIQQSALIKFYICKG-DDCNNYAGEFYVLGDVNEMAWSYDRLYKYEITDALKALGLKHYDRYSIRYEILDLHGNDIG-QL : 1249
LsH1   : ANCAIGQLRKPLSPFSLTSSVNPDANTREHSLPFQVFDYRNNFHYEYDNLEFNGLSIPQLAKELEKNKGDDRVFVGFMLHGIKRSALIKFQIHKNDTEFKEA-GEFYILGDENEMEWSYDRLYKYEVTQQLEDFGLRYNDRYYIKYQVLDLDGSIYSTDT : 1264
LsH2   : ANCAIGQLRQPLAPFSLTSDVNPDAVTREHSVPFQVFDYRNNFHYTYDNLEFNGLTIAQLARKLEQNKDSDRVFAGFMLHGIGKSALVKFYICRSADDCNNYAGEFYILGDPHEMEWAYDRLYKYEITRQLSDLNLHYNDRYYIRYEVLELSGEVFSTNV : 1258
HpHaD  : ANCAIGKLRIPLPPFSLTSDVNPDPVTREHSSPLSVFKYKESFQYEYDTLDFNGLGIPQLARLLEEHKAHDRVFAGFLLHGVGHSALVTFFICRNETDCHNHGGEFYILGDTNEMEWSYDRLYKYEITEELQKLHLRYNDRYFVRYEIHDLTGQDLG-QP : 1257
CaHaD  : ANCAIGKLRKPLPPFSLTSDVNPDPVTREYSSPLRVFNYKKSFQYEYDTLDFNGLGIPQLARVLEEHKSHDRVFAGFLLHGVGHSALVKFFICRNNTDCYNHGGEFYILGDSNEMEWSYDRLYKYEITEELQKLHLRYNDRYFIRYEIHDLTGEDLG-QP : 1257
HpHaN  : ANCATGLLRKPLAPFSLDSDVNPDPVTKAHSQPYQVFDYKDSFHYDYDNLEFNGLTIPQLERVIQHNKAEDRVFAGFLLHGIEHSALLRFNICKTEDHCEPQGGEFYILGDPHEIPWSYNRLFKYEITEQLKSLGLHYDDNYNIHYTLLDLDGTDLG-QR : 1260
CaHaN  : ANCATGLLRKPLAPFSLDSDVNPDPVTKAHSQPHQVFDYKQSFHYEYDNLEFNGLTIPQLERLIQHNKGEDRVFAGFLLHGIQHSALLRFNICKTEDHCEDEGGEFYILGDPHEIPWSYNRLFKYEITEQLKSLGLHYDDSYTIHYTLLDVDGADLG-QR : 1261
HpHb   : ANCAVDKIRKPLAPFSLSSDVNPDPVTREHSLPFQVFDYKTNFHYEYDSLEFNGLTIPQLDRLLQHNKAEDRVFVGFRLHGIKHSALTKFYICKKDD-CDHLAGEFYLLGDENEMAWTYDRLYKYEITEQLADLHLRYDDRYTIKYEVLGLDGKDLG-HV : 1261
CaHb   : ANCAVDKIRKPLAPFSLSSDVNPDPVTREHSLPFQVFDYKTNFHYEYDNLEFNGLTIPQLDRLLEHNKAEDRVFVGFRLHGIQHSALTKFYICKKDD-CSHLAGEFYLLGDENEMAWTYDRLYKYDITEQLADLHLRYDDRYTIKYEVLGLDGADLG-HV : 1261
KLH1   : ANCAVTSMREPLQPFGLSANINTDHVTKEHSVPFNVFDYKTNFNYEYDTLEFNGLSISQLNKKLEAIKSQDRFFAGFLLSGFKKSSLVKFNICTDSSNCHPA-GEFYLLGDENEMPWAYDRVFKYDITEKLHDLKLHAEDHFYIDYEVFDLKPASLGKDL : 1244
OdHG   : ANCAIETLRKPMSPFSLTSDINIDPMTREHSVPFDVFDYKKNFHYEYDLLELNGLSIPQLHREISRRRAKSRIFATFMLEGIKQSALVEYYIRAHGSTDQLKAGEFYILGSANEMPWKFDRVYKADITQQMKEANLHFNDQYHIEYHLKDLSGNEIAGVH : 1250
NpH    : ANCALQQLHQPLSPFSLTSEVNPDPETREHSKPMDVFDYEHNFHYHYDNLELLGMSIPQLSREINRRRSRNRVFAGFMLHGFKKSALVSFSILHHQTDETYA-GEFYILGSEDEMPWHYDRLYRYEITDILNELHLQYTDPLRITYRINDLHNKVVEGEE : 1255
```

```
                       *       1300          *       1320          *       1340          *       1360          *       1380          *       1400          *       1420          *       1440
AcH1  : FPTPTVVKEDGTSQLYGQ-EYREAVTAASYVRRDLSTLNEGEVESLRAAFLSIQKDDTYANIAAFHGKPGLCELN-GR-KVACCVHGMATFPAWHRLYVEQVEEALLGRGSNVAVPYWDWTQPITELPKLINDATYFNSRTQKFEPNPFFSGKVLGEDAVT : 1418
AcH2  : FPTPTVVFEMGTSHLYGQ-EYREAVTTASQVRRDLSTLNEGEVESLRAAFLSIQKDDTYANIAAFHGKPGLCELN-GR-KVACCVHGMATFPAWHRLYVEQVEEALLGRGSNVAVPYWDWTKPITELPKLINDATYFNSRTQKFEPNPFFSGKVLGEDAVT : 1407
LsH1  : FSTPTVLFELGTSNLYGK-EFRAVSTAASQIRRNLDSLSQGEIESLRAAFLSIKEDGTYEKIASFHGKPGKCKDSAGR-TVACCVHGMPTFPAWHRLYVEQVEEALLGRGSSVAVPYWDWTTAFTKLPDLINQATYFNSRQQRFEPNPFFSGSIAGEGAVT : 1423
LsH2  : FPTPTILYDLGTSHLYGK-EYRKPVTAASQIRRDLETLSKGEIESLRSAFLSIKADKTYEKIASFHGKPGLCEHN-GH-KVACCVHGMPTFPAWHRLYVEQVEEALLSRGSSVAVPYWDWTKPITKLPELINEATYYNSREQRFDPNPFFCGTIANENAVT : 1416
HpHaD : FPTPTVIRQIGTSHLYGR-EYRDAVTVASHVRKDLDTLTAGEMESLRSAFLDIQQDHTYENIASFHGKPGLCQHE-GH-KVACCVHGMPTFPSWHRLYVEQVEEALLDHGSSVAVPYFDWISPIQKLPDLISKATYYNSREQRFDPNPFFSGKVAGEDAVT : 1415
CaHaD : FPAPTVIRQIGTSHLYGR-EYRDAVTAASHVRKDLDTLTAGEIESLRSAFLDIQQDHTYENIASYHGKPGLCQHE-GR-KVACCVHGMPTFPSWHRLYVEQVEEALLAHGSSVAVPYFNWITPLQKLPDLISKATYYNSREQRFDPNPFFSGKVAGEDAVT : 1415
HpHaN : YSQPTVIHQVGTSHIYGQ-EYRPVVAAASQVRRNLESLSEGEIESLRAAFLAIQNDHSYEAIASFHGKPGLCEHE-GR-KVACCVHGDPTFPSWHRLYVELVEHALLSHGSSVAVPYWDWISPIKKLPKLISKSTYYNSRQQRFDPNPFFSGRIAGENAVT : 1418
CaHaN : YSQPTVIHEVGTSHLYGQ-EYRPVVAAASQVRRNLESLSEGEIESLRAAFLAIQNDHSYEGIASYHGKPGLCEHQ-GR-KVACCVHGNPTFPSWHRLYVELVEHALLSHGSSVAVPYWDWISPIKKLPKLISKSTYYNSRQQRFDPNPFFSGRIAGENAVT : 1419
HpHb  : FPEPTVIHEVGTSHIYGQ-EYRPLVTAGSHVRHNLEHLSAGEVESLRSAFLAIQEDHSYEDIAAYHGKPGLCEFE-GR-KAACCVHGSAAFPSWHRLYVEQVEHALLAQGSSVSIPYWDWAVPIRSLPKLISEATYFNSRQQRFDANPFFSGRISGENATT : 1419
CaHb  : FPEPTVIHEVGTSHIYGQ-EYRPQVTVGSHVRFNLEDLSAGEIESLRSAFLAIQEDHTYENIAAYHGKPGLCEYE-GH-KAACCVHGSAAFPPWHRLYVEQVEHALLARGSSVSIPYWDWAVPIRSLPKLISEATYFNSRQQRFDANPFFSGRIVGENAST : 1419
KLH1  : FKQPSVIHEPRIGHHEGE-VYQAEVTSANRIRKNIENLSLGELESLRAAFLEIENDGTYESIAKFHGSPGLCQLN-GN-PISCCVHGMPTFPHWHRLYVVVVENALLKKGSSVAVPYWDWTKRIEHLPHLISDATYYNSRQHHYETNPFHHGKITHENEIT : 1402
OdHG  : LE-TAIIYEPGLGNFGEAGIWVEPVTSANRIRKNLNALTDGDMESLRKAFKDMTTDGRYEEIASFHGLPAQCPNKDGSKVYTCCIHGMPTFPHWHRLYVALVENELLARGSGVAVPYWDWVQPFDHLPALVNRATYYNSRTLLVEPNPFFKGKISFLNSET : 1410
NpH   : FS-PTVLYVPALGTYGTQTKWREPVTSASRIRKDLNTLTDGELESLRNAFLRLQEEGAYEPIAAFHGVPAKCRGEDGN-IYTCCVHGMPTFPHWHRLYVIQVEHALRERGSGVSVPYWDWTKRFNHLPALMDESTFYNSRKNEMNTNPFHRGSISFINSHT : 1414

                       *       1460          *       1480          *       1500          *       1520          *       1540          *       1560          *       1580          *       1600
AcH1  : TRDPQPQLFNNDYFYQQTLFALEQTNFCDFEIQFELIHNALHSWLGGRAEYSFSSLDYTAFDPVFFLHHANTDRIWAIWQELQRYRGLPYNEADCAINLMRKPLQPFNNDDFNHGDATNRYSRPADTFDYRNHFQYEYDTLTFNHMTIPQLENLLYQRQE : 1578
AcH2  : TRDPQAQLFNNDHFYQQTLFALEQTNFCDFEIQFELIHNALHSWLGGRAEYSFSSLDYTAFDPVFFLHHANTDRIWAIWQELQRYRGLPYNEADCAINLMRKPLQPFNNDELNHGDLTSRYSRPADTFDYRNHFQYEYDTLTFNHMTIPQLENLLHQRQE : 1567
LsH1  : TRDPQPELFNNDDLYEQVLYALEQDHFCDFEIQFEIIHNALHSLLGGRAQYSLSSLDYSAFDPVFFLHHANTDRIWAIWQELQRYRGLKYNEADCAINQMRTPLRPFNDDQLNHGDLTHRYSRPADTFDYRNHFHYEYDTLEFNHMTVPQLEALLKKRKE : 1583
LsH2  : TRDPQPELFNSEELYEQVLLALEQEHFCDFEIQFELVHNALHTLLGGRSQYSLSSLDYSAFDPVFFLHHANTDRIWAIWQELQRYRGLPYNEADCAINLMRKPLQPFNNDSLNHGDLTHRYSQPAETFDYRNHFHYEYDTLEFNHLTIPQLDNLLKSHQK : 1576
HpHaD : TRDPQPELFNNDYFYEQALYALEQDNFCDFEIQFEVLHNALHSWLGGHAKYSFSSLDYTAFDPVFFLHHANTDRLWAIWQELQRYRGLPYNEADCAINLMRKPLQPFQDKKLNPRNITNIYSRPADTFDYRNHFHYEYDTLELNHQTVPQLENLLKRRQE : 1575
CaHaD : TRDPQPELFNNDYFYEQALYALEQYSFCDFEIQFEVLHNALHSWLGGHAKYSVSSLDYTAYDPVFFLHHANTDRLWAIWQEIQRYRGLPYNEADCAINLMRKPLQPFQDKTLNPRNITNIYSRPADTFDYRNNFHYEYDTLELNHLTVPQLESLLNRRQE : 1575
HpHaN : TRDPQPELFNNDYFLEQALFALEQDHYCDFEIQFEILHNALHSWLGGHALYSMASLDYSAFDPVFFLHHANTDRIWAIWQELQRYRGLQYNEADCAINLMKKPLQPFNRTT-NTDEVTRKYSRPVDTFDYRNNLHYEYDTLEFNHLSIPQLEELLQSRKR : 1577
CaHaN : TRDPQPELFNNDYFLEQALFALEQDHYCDFEIQFEILHNALHSWLGGHAFYSMASLDYAAFDPVFFLHHANTDRIWAVWQELQRYRGLQYNEADCAINLMRKPLEPFNRVT-NTDEVTRKNSRPVDTFDYRNNLHYEYDTLEFNHLSIPQLEELIQSRKR : 1578
HpHb  : TRNPQTALFNNDYFYEQVLFAFEQTHFCDFEIQLEVTHNAIHSWLGGHARYSMASLDYAAFDPVFFLHHTNTDRLWAIWQELQRYRGLPYNEADCAINQMRKPLKPFGDSD-NKDNVTKKYSRPVDTFDYRNHFDYEYDNLEFNHQSIPQLENLLKLRQR : 1578
CaHb  : TRNPQAALFNNDYFYEQVLFAFEQTHFCDFEIQLEVTHNAIHSWLGGHARYSMASLDYAAYDPVFFLHHTNTDRLWAIWQELQRYRGLPYNEADCAINQLRKPLKPFGDSD-NKDNVTRKYSRPIDTFDYRNHFDYEYDNLEFNHQSIPQLENLLKQRQT : 1578
KLH1  : TRDPKDSLFHSDYFYEQVLYALEQDNFCDFEIQLEILHNALHSLLGGKGKYSMSNLDYAAFDPVFFLHHATTDRIWAIWQDLQRFRKRPYREANCAIQLMHTPLQPFDKSD-NNDEATKTHATPHDGFEYQNSFGYAYDNLELNHYSIPQLDHMLQERKR : 1561
OdHG  : NRDPQEELFGNKYLYEHTLFVLEQTDFCDFEVHFEVLHNTIHSWLGGRDPHSMSSLDFAAYDPIFFLHHSNIDRIWAIWQELQRYRKLPYNEANCALPLLNVPMRPFSNTTANHDRMTLTHSAPNDVFDYQNVLHYKYDTLSFYDLTITQLDHLIEERKS : 1570
NpH   : SRDFQPELFNSDYLYENTLLALEQNDFCNFEVQLEVLHNAIHAWIGGRDTYSMSSLDYAAYDPIFFIHHSTLDRIWAIWQELQRYRGLPYNEANCALPLMNQHLRPFNNVSVNHDRNTLTLNKPNDAFDYQNHFHYRYDNLDFHGLSIPQLEHELHERQT : 1574

                       *       1620          *       1640          *       1660          *       1680          *       1700          *       1720          *       1740          *       1760
AcH1  : SGRVFAGFLLHNIGASADVEIYICVPTGP-RGKKNCGTRAGVFSVLGGQLEMPFVFDRLYKYDITRAVRDLGLRPDSGAKFDLKINIHAVNGSYLNPSLLPAPTVLYVPGSN--ERQEADGQVGNYLVRKNIASLSPQEELSLLKAMAALQADSSADGYQ : 1735
AcH2  : NGRVFAGFLLHNIGASADVEIYICVPTGP-RGKKNCGNKAGVFSVLGGQLEMPFVFDRLYKYDITHAVRDLGLKLDNAANFELKIDIRAVNGSFLSPSILPSPTVFYVPGSN--ERQESDGQLSSYQVRKSIDALSPQEELSLLKAMAALQADSSADGYQ : 1724
LsH1  : SGRVFAGFLLHNIGLSAVANIYVCVPSGP-RGKKSCNHPAGSFSILGGELEMPFVFDRLYKYDITKTVRDLGLKLDNAANFELRVDIKAVNGSLLDSDILPKPTILFEPGTG--ERQKSSGEVTNYLVRKEINSLSPREVQSLYSAMEALQADSSADGWQ : 1740
LsH2  : SGRVFAGFLLHNIGLTAVVDIYVCVPSGA-HNKQSCNHKAGSFSILGGELEMPFAFDRLYKFDITRAVRDLGLKLDNAANFELKVDIRAVNGSLLDSSILPPPTIIFVPGTS--ERQEAHGAVSSFVVRKNVDALSQREALSLYNAMRALQADSSADGFQ : 1733
HpHaD : YGRVFAGFLIHNIGLSADVTVYVCVPSGP-KGKNDCNHKAGVFSVLGGELEMPFTFDRLYKLQITDTIKQLGLKVNNAASYQLKVEIKAVNGTLLDPHILPDPSIIFEPGTK--ERQAEDGDVKDIVVRKNVDALSPRETLSLIHALEALQADSSADGYQ : 1732
CaHaD : YGRVFAGFLIHNIGLSADVTVYVCVPSGP-KGKNDCNHKAGVFSVLGGELEMPFTFDRLYKLQITDTIKQLGLKVNNAASYQLKVEIKAANGTLLDPHILPDPSIIFVPGTR--ERQTEDGDVKHVVVRKNVDALSPRETLALIHALEALQADSSADGYQ : 1732
HpHaN : NGRVFAGFLIHNIGLSADVDVYVCVTVGQ-FGEQDCNHKAGTFSVLGGETEMPFEFNRLYKQDITRTVRELGLKLDNAANFHLLVEIRAPNGSQLDPHILPDPSIIYIPGTD--EVEDQHGSTSTYLVRKNVESLSPLEGYYLAEALIALKKDTSADGYQ : 1734
CaHaN : NGRVFAGFLIHNIGLSADVDVYVCVSVGK-YGEQDCDHKAATFSVLGGETEMPFEFNRLYKQDITRTVRELGLKLDYAANFHLLVEIKAPNGSQLDPHILPDPSIIYIPGTD--EEQDQLGATTTYLVRKNVESLNPLEGYHLADALIALKKDTSADGFQ : 1735
HpHb  : KGRVFSGFLIHNIGLSADVEVSICLPNAP-DAFGGCNHKAAVFSILGGETEMPFEFDRLFRIDITKAVKDAGLEVSAAANFKLKVAIRAVNGSYLDPHILPDPSIIYDPGTD--EAEHPHGDRAPLLVRKNVESLSPLEKYHLVKALTSLNADGSADGFQ : 1735
CaHb  : KGRVFAGFLIHNIGLSADVEVSICLPNAP-DAFGSCDHKAAVFSILGGETEMPFEFDRLFRIDITKAVVDAGLEPSASANFKLKVAIRAVNGSYLDPHTLPDPSIIYDPGTD--DVELPHGGRAPSLVRKNVESLSPLEKYHLVKALTSLNADGSADGFQ : 1735
KLH1  : HDRVFAGFLLHNIGTSADGHVFVCLPTG--EHTKDCSHEAGMFSILGGQTEMSFVFDRLYKLDITKALKKNGV--HLQGDFDLEIEITAVNGSHLDSHVIHSPTILFEAGTD--SAHTDDGHTEPVMIRKDITQLDKRQQLSLVKALESMKADHSSDGFQ : 1715
OdHG  : HDRIFAGFLLHGVQASADIHVFICVPTS--KHEENCAHDVGVFSVLGGKSEMPWQFASVFQYEITDQLKLLGL--NQNSHFRGVTEVTAVNGSSINSDIFPHPTIIYVPKQD-HSADIKSEEGNEYLVRKNVERLSLSEMNSLIHAFRRMQRDKSSDGFE : 1725
NpH   : HDRVFVGFLLHGIQASAYVRIYICVPTQRGSSKENCNNYAGIFSILGGVTEMPWHFDRLYYYEITHALSNLGL--SHRSHFRIKTDINAVNGTHFDSHIFPDPTISFVPAPGSKNATHYQEEEHHHFVRKEVSRLSIYEIHEVVYAFRRMQRDMSADGFE : 1732

                       *       1780          *       1800          *       1820          *       1840          *       1860          *       1880          *       1900          *       1920
AcH1  : SIASFHAIPPLCPSPSASKRYACCLHGMATFPQWHRLYTVQVEDALRRHGSLVGIPYWDWSRQTDHLPGLLANPTYTDVYTGQTIDNPWYKGKIEF--ENSVTERDVQGDYLFKQGPHGFDTWLFNQALLALEQEDYCDFEIQFEITHNAVHSWLGGTKV : 1893
AcH2  : SIASFHAIPPLCPSPSASKRYACCLHGMATFPQWHRLYTVQVEDALRRHGSLVGIPYWDWSRQTDHLPGLLANPTYTDVYTGQTIDNPWYKARIEF--ENTTTERDVQGDYLFKQGPHGFDTWLFNQALLALEQEDYCDFEIQFEITHNAVHSWLGGTKV : 1882
LsH1  : SIASFHAIPPLCPSPSAENRYACCLHGMATFPQWHRLYTVQVEDALKRHGALVGIPYWDWSRVSDSLPHFIDDENFVDPVTGDQKANPWKKARIEF--ENSETEREVVSDRLFKRGPHGWDTWLFNQALLALEQEDYCNFEIQFEITHNAIHSWVGGSKE : 1898
LsH2  : AIASFHALPPLCPSPAAINRYACCLHGMATFPQWHRLYTVQVEDALRRHGSLVGIPYWDWTRQSKSLPEFFSDPTYPDPDGGDPVDNPWYKANIEF--EHAVTERDVKEEFLFKVGKHGWDTWLFNQALYALEQEDYCDFEVQFEITHNAIHSWLGGSKN : 1891
HpHaD : SIAAFHAVPPLCPSPSASTRYACCLHGMSTFPQWHRLYTVQVEDALRRHGSVVGIPYWDWTRASQSLPHFLSDNNYTDPYTKEVHDNPWHGASIDF--EHSHTERDIQSAELFKLGPHGWDTWLFEQALLALEQEDYCDFEIQFEITHNAIHSWVGGSKE : 1890
CaHaD : SIAAFHAVPPLCPSPSASKRYACCLHGMSTFPQWHRLYTVQVEDALRRHGSGVGIPYWDWTRASQSLPGFLSAENYTDPYTKEVHENPWHGASIDF--ENSHTERDIQSAELFKLGPHGWDTWLFEQALLALEQEDYCDFEIQFEITHNAIHSWVGGSKE : 1890
HpHaN : SIATFHAIPPLCPSPTASRRYACCIHGGTSFLQWHRLYTVQFEDALKRHGSPIGVPYWDWTRFSKELPRTFTYANYSDPFTNLWTLNPFYSGRVEF--EHVDTERDVQEDKLFKRGPHGWDTWLYTQVLFALEQEDFCDFEVQLEIAHNAIHSWIGGSKE : 1892
CaHaN : SIATFHAIPPLCPSPTASLRYACCIHGGTSFLQWHRLYTVQFEDALKRHGSPIGVPYWDWTRFSTELPRTFTFTNYSDPFTNVWTLNPFYKGRVEF--EHVDTERDVQEDKLFKSGPHGWDTWLYTQVLFALEQEDFCDFEVQLEIAHNAIHSWIGGSKE : 1893
HpHb  : SIATFHAIPPLCPSPTASVRHACCIHGGAAFLQWHRLYTVQFEDALRRHGSHVGLPYWDWVRITDRLPSIFTVESQTDPSTNVSVPNPFATGRVEF--EHEDVHRDIQSNLLFKRGPHGWDTWLYNQALYALEQEDYCDFAVQLEITHNSIHAWIGGSQK : 1893
CaHb  : SIATFHAIPPLCPSPTASVRHACCIHGGAAFLPWHRLYTVQFEDALRRHGSHVGLPYWDWVRISDRLPSILTVESQTDPSTNVSVPNPFATGRVEF--EHEDVHRDIQSDLLFKRGPHGWDTWLYNQALYALEQEDYCDFAVQLEITHNSIHAWIGGSQK : 1893
KLH1  : AIASFHALPPLCPSPAASKRFACCVHGMATFPQWHRLYTVQFQDSLRKHGAVVGLPYWDWTLPRSELPELLTVSTIHDPETGRDIPNPFIGSKIEFEGENVHTKRDINRDRLFQGSTKTHHNWFIEQALLALEQTNYCDFEVQFEIMHNGVHTWVGGKEP : 1875
OdHG  : AIASFHALPPLCPSPTAKHRHACCLHGMATFPHWHRLYVVQFEQALHRHGATVGVPYWDWTRPISKIPDFIASKRYSDPFTKIEDYNPFNQGQISFISEDTETKREVS-EYLFEHPVLGKQTWLFDNIALALEQTDYCDFEIQLEIVHNAIHSWIGGKEE : 1884
NpH   : YIASYHADPPLCPNPKALHRYACCIHGMATFLHWHRLYVVQFEQSLHRHGSDIGFPYWDWTRPITHIPALLKDVNYTDPFTNSVVGNPFNHGHLSFISPEVMTQRDVQ-DKLFKQPKLGRNTWLHDNVILALEQTDYCNFEVQFEIVHNAIHAWVGGSQE : 1891
```

```
                 *      1940        *      1960        *      1980        *      2000        *      2020        *      2040        *      2060        *      2080
AcH1   : HSMGHLHYASYDPAFYVHHSNTDRLWALWQALQKYRGLNPNEANCALEQMRVPLKPFSFGAPYNLNPKTKEFSKPEDTFEYEEHFNYNYDHLEFVGLDIPALDGFIKEKQEEDRVFAGFLLKGFGSSAFVNFDIVSN--GKSFEGGYFTVLGGSAEMPWQ : 2051
AcH2   : HSMGHLHYASYDPAFYVHHSNTDRLWALWQALQKYRGHNPNEANCALEQMREPLKPFSFGSPYNLNEMTKEYSKPEDTFDYEGHFNYRYDNLEFVGMNIPRLDAFIKERRERSRVFAGFLLKGFGSSAMVTFDICTKGMESCFQGGYFTVLGGAAEMPWQ : 2042
LsH1   : HSLGHLHYASYDPAFFVHHSNTDRLWAVWQALQKYRGYDPNEANCALEQMKDPLKPFSFGPPYNLNPLTKEHSRPEDTFNYADHFHYRYDNLEFVGLSIPQLDAFIKERHEHDRVFAGFLLKGFKKSALVDFDICAN--GNCFPGGYFTVLGGSAEMPWQ : 2056
LsH2   : YSLGHLHYASYDPAFFIHHSNTDRLWAVWQALQKYRGHDPNEANCALELMREPLKPFSFGPPYNLNQMTQDNSRPEDTFNYEDHFHYRYDDLQFVGMSIPRLDAFIKEKQEHDRVFAGFLLKGFGQSALVSFEICNDDMSNCFEGGYFTVLGGSQEMPWR : 2051
HpHaD  : HSLAHLHYASYDPAFYIHHSNTDRLWAIWQALQKHRGYSPNEANCALEHLKDSLKPFSFGPPYNLNKLTTKYSHPEDTFAYEDHFEYQYDSLEFVGMNIPALDAYIKERQEHDRVFAGFLLHGLGTSASVNFTICDAF-KKCFEGGHFTVLGGELEKPWQ : 2049
CaHaD  : HSLAHLHYASYDPAFYIHHSNTDRLWAIWQALQKHRGYNPNEANCALEHIKDSLKPFSFGPPYNLNKLTTKYSHPEDTFAYEEHFEYQYDSLEFVGMTIPALDAYIKERQEHDRVFAGFLLRGLGTSATVDFTICDAF-KKCFDGGHFTVLGGEQEKPWQ : 2049
HpHaN  : HSLAHLHYASYDPAFFIHHSNTDRLFAVWQKLQTYRGHDANSANCALEQLREPLKPFSFGPPYNLNKVTQDNSRPEDTFAYKEHFHYKYDALEFVGMNIPTLDAYIKERQEHDRVFAGFLLKGIGQSASVKFEICIDATGVCFDGGEFSILGGSAEMPWQ : 2052
CaHaN  : HSLAHLHYASYDPAFFIHHSNTDRLFAVWQKLQQYRGHDPNSANCALEQLREPLKPFSFGPPYNLNKVTQEYSRPQDTFAYKEHFHYKYDALEFVGMNIPTLDAYIKERQEHDRVFAGFLLKGIGQSATVEFEICSDATGDCFDGGEFSILGGSAEMPWQ : 2053
HpHb   : HSMAHLHYASYDPIFFIHHSNTDRLWAVWQALQEYRGHSANEVNCALEKLREPLKPFSFGPPYNLNPVTLENSHPGDTFDYERHFHYRYDALEFVGMNIPTLDAYIKERQEHDRVFAGFLLKGIHQSATVDFDICKG--NNCFEGGYFSILGGPAEMEWE : 2051
CaHb   : HSMAHLHYASYDPIFFIHHSNTDRLWAVWQALQKHRGHNAYEVYCSLEKLREPLKPFSFGPPYNLNPVTRQNAHSGDTFYYESHFHYRYDALEFGGMNIPTLDAFIKERQEHDRVFAGFLLKGIHQSATVDFDICKGDTDNCFPGGYFSILGGPAEAEWE : 2053
KLH1   : YGIGHLHYASYDPLFYIHHSQTDRIWAIWQSLQRFRGLSGSEANCAVNLMKTPLKPFSFGAPYNLNDHTHDFSKPEDTFDYQK-FGYIYDTLEFAGWSIRGIDHIVRNRQEHSRVFAGFLLEGFGTSATVDFQVCRTA-GDCEDAGYFTVLGGEKEMPWA : 2033
OdHG   : HSLNHLHYAAYDPIFYLHHSNVDRLWVIWQELQKLRGLNAYESHCALELMKVPLKPFSFGAPYNLNDLTTKLSKPEDMFRYKDNFHYEYDILDINSMSINQIESYIRHQRDHDRVFAGFLLSGFGSSAYATFEICIEG-GECHEGSHFSVLGGSTEMPWA : 2043
NpH    : HSLSHLHFAAYDPAFYLHHSNVDRIWVIWQELQKIRGLSAYEANCALELMNEPLKPFAFGSPYNLNEMTRKYSLPEDTFNYKDHFDYEYDMLEFSGMTIPQLEDYIKESLHHERIFAGFLLHGIGQSANVDFEVCDNQ-NHCEDAGYFSILGGSLEMPWA : 2050

                 *      2100        *      2120        *      2140        *      2160        *      2180        *      2200        *      2220        *      2240
AcH1   : FDRLFKYEITDQLIAANLRFDDNYSFNISVRLPDGTVLDSSLIPTPSVLFKSAQHDFKREHVAPNHVRRNLDTLEERDLQSLKAALRDLQHDNSNDGWASLASFHGSPAKCSTPANESVACCIHGMPTFPHWHRLFTLQVEQALRRHGSAIAIPYWDWTL : 2211
AcH2   : FDRLFKYEITDQLSEHNLRPDDDYHFHVHVKYIDGTELDHSLVPNPSVIIEPASDDYNLEKVTPNHIRRNLDELSERDLQSLKAALRDLQLDTSNDGWANLASFHGAPARCPDPKNPTVACCVHGMPTFPHWHRLYTLQVEQALRRHGSAIAIPYWDWTQ : 2202
LsH1   : FDRLFKYEITDQLNEHKLRFDDEYTFQIKIRAPDGTELSSDLIGTPSVILEPFKRDYDLEKVTPNFVRHDLSTLTERDLQGLKSALRDLQLDESADGWASLASFHGAPSMCENESGAKVACCIHGMPTFPHWHRLFTVQVERALQRHGSAVAIPYWDWTK : 2216
LsH2   : FDRLYKYEITEQLEEHNLRYDDNYHFHVHVKNIDGTMLPGKLIPDPTVILVPAQSDHDLDNVTPNLIRRDLSTLSERDIQSLQAALRDLQLDQSNDGWASLASFHGAPNRCPDPANPTVACCQHGMPTFPHWHRLFTVQIEQALQRHGSAIAIPYWDWTK : 2211
HpHaD  : FDRLYRYPITDVLKENHIHYDDDYHFHIHIKALNGTELDSKLIPEPSVLFVAAHEDVHHVEATPNNIRRNLDTLEERDIQSLQAALRDLQRDTTNDGWTNLASFHGAPARCPDPSHPTVACCVHGMPTFPHWHRLFALQIEQALHRHGSSIALPYWDWTQ : 2209
CaHaD  : FDRLYRYPITDVLKENHIHYDDDYHFHIHIKALNGTELDSKLIPEPSVLFVAAHEDVH-VETTPNNIRHNLNSLEERDIQSLQAALRNLQRDTTNDGWASLASFHGAPARCPDPDHPKVACCVHGMPTFPHWHRLFALQIEQALHRHGSSIALPYWDWTL : 2208
HpHaN  : FNRLYKYEITDQLESHHVHYDDEYHLTVHLKSINGTELDSHLIHEPTVIYVPAKHDVTLKKVTVNHIRRNLDEVEERDTQSIQRALRDLLADTGPDGWASLASFHGAPARCPDPDHPTVACCQHGMPTFLHWHRLFTLQVEQAIQKHGSAIAIPYWDWTQ : 2212
CaHaN  : FNRLYKYEITDQLESHHVHYDDEYHLTVHLKSINGTELDSHLVHEPTVIYVPAKHDVTLKKVTVNHIRRNLDDVEDRDTQSIQTALRKLLADTGPDGWASLASFHGAPARCPDPDHPTVACCQHGMFTFLHWHRLFTLQVEQAIQKHGSSIAIPYWDWTQ : 2213
HpHb   : FDRLYNYEITDQLKEHHVRYDDDYNFKIQIIAPDGAILDSHLLHEPTVSFVPARHDLNLKKVPLNKIRRNIDSLEERDIQSLQTALHDIQEDDSNNGWANLASFHGAPARCPDPEHPKVACCQHGMPTFPHWHRLFTLQVEQALQAHGSSLAIPYWDWTL : 2211
CaHb   : FDRLYNYEITDQLKEHHVRYDDDYHFKIQIKAPDGTILDSSLLREPTVSFAPAKRDIHLRKVPLNKIRRNIDSLEERDIQSLQSALYDLQEDESNNGWANLASFHGAPARCPDPEHPKVACCQHGMQTFPHWHRLFTLQVEQALRDHGSSLAIPYWDWTL : 2213
KLH1   : FDRLYKYDITETLDKMNLRHDEIFQIEVTITSYDGTVLDSGLIPTPSIIYDPAHHDISSHHLSLNKVRHDLSTLSERDIGSLKYALSSLQADTSADGFAAIASFHGLPAKCNDSHNNEVACCIHGMPTFPHWHRLYTLQFEQALRRHGSSVAVPYWDWTK : 2193
OdHG   : FDRLYKIEITDILSDMNLAFDSAFTIKTKLVAQNGTELPASILPEATVIRIPPSNEDADIDTPLNHIRRNVESLDERDIQNLMAALTRVKEDESDHGFQTIASYHGS-TLCPSPEEPKYACCLHGMPVFPHWHRVYLLHFEDSMRRHGSSVATPYWDWTQ : 2202
NpH    : FNRLYRYDITHTLETMHLNYDDNFHINIHIHDMAGNDLDSHLLQKATILRQPAAE-HHEVHHPLNRIRHNINDLSERDIQSLKSALIKVKEDHSNFGFQTITSYHGLPASCPTPEEATYACCMHGMAAFPHWHRLYALQFEESLLRHGSSVALPYWDWTH : 2209

                 *      2260        *      2280        *      2300        *      2320        *      2340        *      2360        *      2380        *      2400
AcH1   : PITDLPEIFTSQNYYDVWRDEVVNNPFARGYVPTEDVYTVRDIRPEIRNKNQAGDHSAIFDLVLSALEQTDYCDFEVQFEVMHNAIHFLVGGLQTYSLSSLEYSAYDPLFFIHHSFTDKIWAVWQELQTRRHLPANKADCALNYMSQPMRPFFFEGFNLN : 2371
AcH2   : PISHLPEIFTSEDFYDVWRDEVVANPFARGYIPTEKAYTVRDVRPEIHLNDREGRHSALFDLVLLALEQTDYCDFEVQFEVMHNAIHFLVGGLQTYSMSTLEYSAYDPMFLIHHSFTDKIWIVWQELQKRRHLPATSAACAVNYMNQPMKPFYFEGFNLN : 2362
LsH1   : PITALPEIFTKEDFYDAWRDEVVDNPFARGFIPTENTYTVRDIRPELFQTSKDGSHSDLFELVLSALEQTDYCDFEVQFEVMHNAIHYLVGGLQTYSLSSLEYSAYDPIFFIHHSFVDKIWVVWQELQQRRHLPSNTANCALNYLAEPMKPFNLD-LNIN : 2375
LsH2   : PIDELPKIFTDEDFYDVWRDEVVANPFARGYVPSEKVYTVRETKSELYDKGRGGKNSALIYYVISALEQTDYCDFEVQFEVIHNSIHYLVGGEQTYSLSSLEYSAYDPIFFIHHSFTDKIWAVWQELQKRRRLPYLKANCALNYMAEPMKPFSIDGLNLN : 2371
HpHaD  : AIDDLPSTFTKEDYYDVWRDEVVPNPFAHGYVTSEDTYTVRDIQETLHDRHVDGKHSFLLYGVLEVLEQTDYCDFEVHFEVVHNAIHYLIGGHQTYSLSSLEYSAYDPIFFIHHSFTDKLWAVWQELQKRRHFPYNRADCALNYVNEPLKPFSFEGFNLN : 2369
CaHaD  : AIDELPSTFTKEDYYDVWRDEVVPNPFAHGYVASEDTYTVRDIQSPLHKKH-DGKHSFLLHGVLDVLEQTDYCDFEVQFEVVHNAIHYLIGGHQTYSLSSLEYSAYDPIFFIHHSFTDKIWAVWQELQKRRNLPYNRADCALNYVNEPLKPFSFEGLNLN : 2367
HpHaN  : PIKKLPDIFTKVNYYDAWSDQVLENPFAHGSIPSEKAQTVRDVQPELFETTKDGKHSTLFPLLLEALEQTSYCDFAVQFEVLHNAIHYLVGGHQKYSLSSLEYSAYDPIFFIHHSFSDKLWVVWQELQQRRHLPYHTADCAVSAMSEPMKPFSFETFNTN : 2372
CaHaN  : PLKKLPDIFTRVNYYDAWSDQVLENPFAHGSIPSEKAQTVRDVQPELFETTEDGKHSTLFPLLLEALEQTSYCDFAVQFEVLHNAIHYLVGGHQKYSLSSLEYSAYDPIFFIHHSFSDKLWVVWQELQQRRHLPYHTADCAVNAMSEPMKPFSFETFNTN : 2373
HpHb   : HVSELPKIFTEEDYYDVWKDEVLENPFAHGYIPSEGAYTVRDVYQHFFRAYGDGKHSFLFYIALEALEQTDYCDFEVQFEILHNAIHYLVGGEQKYSLSSLEYSAYDPLFFIHHSFVDKIWVVWQKLQQRRHLPYNRADCAVAAVNQPMKPFTFESINPN : 2371
CaHb   : HVSELPKIFTEEDYYDVWKDSVLENPFAHGYIPSEDAYTVRDVYPGFYKSFGDGKHSFLFIIALQALEETDYCNFEVQFEILHNAIHYIVGGEQKYSLSSLEYSAYDPLFFIHHSFTDKIWVVWQELQQRRHLPYNRADCAVVAMNQPMKPFTFESLNLN : 2373
KLH1   : PIHNIPHLFTDKEYYDVWRNKVMPNPFARGYVPSHDTYTVRDVQEGLFHLTSTGEHSALLNQALLALEQHDYCDFAVQFEVMHNTIHYLVGGPQVYSLSSLHYASYDPIFFIHHSFVDKVWAVWQALQEKRGLPSDRADCAVSLMTQNMRPFHYE-INHN : 2352
OdHG   : PGTKLPRLLADSDYYDAWTDNVTENPFLRGYIKSEDTYTVRDVKPELFEIG-GGEGSTLYQQVLLMLEQEDYCDFEVQFEVVHNSIHYLVGGHHKYAMSSLVYSSFDPIFYVHHSMVDRLWAIWQALQEHRHLPFDKAYCALEQLSFPMKPFVWD-SNPN : 2360
NpH    : KITGLPHLLSNPDYYDALHKQVTENPFLRGHIEVAETYTVRNTQPELFEPSKDGKHSLLYDEILLALEQRDYCDFEVQFEVVHNAIHYLVGGHQRYSMSSLEYASYDPLFFIHHSNVDRLWAIWQALQQYRRIPYNKAYCALEQMTHAMKPFDRE-TNPN : 2368

                 *      2420        *      2440        *      2460        *      2480        *      2500        *      2520        *      2540        *      2560
AcH1   : KFTMDHAVPNTVFDYEHLGYSYDDLSIGGYDLDGLEQVIADRQSKARVFAGFLLKGVKTSGSVVINICLRNNVCNYAGRFNLLGGPTEMAWAFDRLFTYDITSALEQSGINPEDVFDAEALFTLDIKVFDVEGHALPVSSVLPEPTIIYKAAVGASEEVS : 2531
AcH2   : GFTKSHALPSSVFDHEKLGYAYDDLTIGGYDLEGLEDLITARRSHARVFAGFLLKGAKTSGSAVIDICRPNNECAYGGRFNVLGGPLEMDWAFNRLFKFDITRALEEAGLHPEDVFDSEALFTLRVKIFNAEGQAVALKSVMPEPTIIFEPAHGAAEDV- : 2521
LsH1   : AFTKKHAVPRDVFNHEELGYHYDNLNIGGYNLDQLEQLIAQHQSRPRVFAGFLLKGIKTSGSVVLNVCKG-SQCTYAGRFNLLGGPSEMPWAFDRLYKKDITWALEAADIKPESVFDADSLFTLDVKVFDVEGHAINVKSVFPTPSIIYDPAKGVANVD- : 2533
LsH2   : KFTKEHAVPDKLFDYEELGYAYDTLEIGGYSLEQIEEIIADNKKTARVYAGFLLKGIKTSGSVIIKACKD-SECAFAGRFNLLGGPTEMPWAFDRLFKADITWALRNLGLTADDVTDVDSHFHLEVDITDVKGRSLNRKSVMPEPTIIFRPAEGSTQDA- : 2529
HpHaD  : KFTREHAVPNTLFNHEDLGYAYDNFNIGGYNLDELEKLIHDRQIKPRIFAGFLLKGIKTSGSVNLKVCKF-EECTPAGFFNLLGGPLEMPWAFDRLYKKDITWAIAKLGLNPDDIHNTDSGFKLEVQAFNVEGTALPLSQVMPTPSVIYKPALGVKKDI- : 2527
CaHaD  : KFTREHAVPNTLFNHEDLGYAYDNFNIGGYDLDQLEKLIHDRQIKPRIFAGFLLKGIKTSGSINLKVCKL-EECTSGGFFNLLGGPLEMPWAFDRLYKKDITWAIANIGLNPDDIHKTDSGFRLEVQAFNVEGTALPLSQVMPTPSIIYKPAFGVDKNI- : 2525
HpHaN  : KFTRDHAGPNSLFDHENLGYTYDHLKVGGYDLGQLEELIHDNQQHARVFAGFLLHGIKTSGSVKIKLCQG-DKCTSAGQFNLLGGPLESPWAYNRLYKRDITQYLEDLNLHPEDVFDPILQVHLEVEVRDVQDHLLVAQDVLPQPTIIFDPPHEGAEDV- : 2530
CaHaN  : KFTREHSVPNTVFDHENLGYTYDNLNVGGYSLDQLEELIHDNQQHARVFAGFLLHGIKTSGSVKIKLCQG-DKCTSAGQFNLLGGPLESPWAYNRLYKRDITQYLAALNLHPEDVFDPTLQVHLEVEVRDVQDHVLEAQNVLPQPTIIFDPPHEGAEDV- : 2531
HpHb   : KFTREHALPSSVFDHENLGYGYDNLNIGGYTIEELEALIHDREKEDRVFVGFLLKGIGTSGVVTINICLRSGNCTFSGRFSLLGGPLEAPWAYDRLYKKEITHYLADIHIEPKDVLNAS--VYLKIEVDDVEGRTLSPKAVFPNPTIIFKAGHEEAKKT- : 2528
CaHb   : KFTREHALPSSVFDHEDLGYGYDNLNIGGYTIDELEELIHERQKEDRVFAGFLLKGIHTSGVVTINVCVKSGNCTFAGRFSLLGGPLEAPWAYDRLYKREITQYLGEIHIKPRDILNAN--VYLKIEVDDVEGRTLSPKAVFPHPTLIFEPGHDEEEKA- : 2530
KLH1   : QFTKKHAVPNDVFKYELLGYRYDNLEIGGMNLHEIEKEIKDKQHHVRVFAGFLLHGIRTSADVQFQICKTSEDCHHGGQIFVLGGTKEMAWAYNRLFKYDITHALHDAHITPEDVFHPSEPFFIKVSVTAVNGTVLPASIL-HAPTIIYEPGLDHHEDH- : 2510
OdHG   : LHTRAASTPQHLFDDNKLGYKYDNLEFHGMNIDQLENAIHKQQNKDRVFASFLLFGIKTSADVHLKLC-KDETCEDAGVVFILGGDNEMPWHFDRTYKKDITHVLHQMHIPLEDLYVHGSTILLEVEIETVDGKVLDSSSL-PAPSMIYVPAKDFKREV- : 2517
NpH    : KNTHDHAIPNSVFNYDDLGYSYDNLLFDGLTITELEEKIHSRQSHPRVFAGFLLHAIGTSADVHVSVCHHGNNCTSAGLFFILGGKHEMPWKYDRLFRYDITDAVTKAGFSPEDVFNDNLLYKIHTEINAVNGSHIDSHIM-HEPTLIFVPGRNTKQAER : 2527
```

```
                 *      2580         *      2600         *      2620         *      2640         *      2660         *      2680         *      2700         *      2720
AcH1  : SSSSLAGVGVRKDVSTLSTSEIDNLREALRRVQADAGPNGFASIAAFHGEPAGCEL-NGRRIACCQHGMANFPQWHRLFVKQFEDALTAQGAMIGIPYWDWTTAFTALPSLVTEQENNPFYNFKI-FNGEVTSRAPREQLFNDPEFGSESFFYRQALLAF : 2689
AcH2  : SSTTLSGVGVRKDVSTLSAAEISNLRDALRKVQADVGPNGFASIAAFHGEPAGCEL-NGHPIACCQHGMANFPQWHRLFVKQFEDALTAQGAMIGIPYWDWTTAFTALPSLVTEQENNPFYNFKI-FNGEVTSRAPREQLFNDPEFGSESFFYRQALLAF : 2679
LsH1  : ITSAVAGVGVRKDVSSLTASETESLRNALRKVQEDEGPNGFQNIASFHGSLAKCEY-QGHPIACCLHGMANFPQWHRLYVKQWEDALTSQGSKVGIPYWDWTTAFTALPTLVTEETNNPFHHGKI-YNGEITTRAPRDQLFNDPEFGSESFFYRQALFTF : 2691
LsH2  : VSTSIAGVGVRKDVSTLTVSEIENIRNALKRVQEDDGPNGFQNIASFHGSPAKCEY-QGHPIACCLHGMANFPQWHRLYVKQWEDALTAQGAKIGIPYWDWTTAFTALPTLVSQEDDNPFHHGKI-YNGEVTTRAPRDKLFNDPEFGAESFFYRQVLLAF : 2687
HpHaD : HTTAVAGVGVRKDVTRLTVSETENLREALRRIKADNGSNGFQSIASFHGSPPGCEH-ENHSVACCIHGMANFPQWHRLYVKQWEDALTAQGAKIGIPYWDWTTAFTELPALVTEEVDNPFHHGTI-YNGEITTRAPRDKLFNDPEFGKESFFYRQVLLAF : 2685
CaHaD : HTTAVAGVGVRKDVTRLTVSETENLREALRRVKADNGSNGFQKIASFHGSPPGCVH-ENHYVACCIHGMANFPQWHRLYVKQWEDALTAQGAQIGIPYWDWTTAFTELPALVTEEDDNPFHHGKI-YNGQNTTRAPRGRLFNHPEFGRESFFYRQILLAF : 2683
HpHaN : SSTSIAGVGVRKDVSSLSTSEIDNIRSALQQVEDDTGPNGFQNIASFHGSPARCEH-DHHPVACCVHGSPNFPQWHRLYVKQWEDALTAHGAKIGIPYWDWTYAFKKLPSLVTAGDNNPFHHGVT-HDGHITTRAPRSLLFNDPEFGDESFFYRQVLLAF : 2688
CaHaN : SSTSIAGIGVRKDVSTLSTSEIYNIRSALRQVKDDAGANGFLNIASFHGKPARCEH-DHHPVACCVHGSPKFPHWHRLYVKQWEDALTAHGAKIGIPYWDWTYAFKKLPSLVTAEVDNPFHHGVT-HDGHVTTRAPRSLLFNDPEFGDESFFYRQVLLAF : 2689
HpHb  : KAVPVPGDSVRKNVNDLTNSDVANLRAALRDVQADDGANGFASIAGFHGSPAHCEH-DHHPVACCLHGMANFPQWHRLYVKQWEDALIAHGSKNGIPYWDWTQSFTELPTLVTQVEDNPFHHGKI-DKDHNTTRSPRPQLFSDPASGNESFFYRQVLLAF : 2686
CaHb  : ARVPVPGDSIRKNVNDLTDSEVANLRAALYDVQADTGPNGFAFIASFHGYPARCEH-DNHPVACCLHGMASFPQWHRLYVKQWEDALISHGSREGVPYWDWAQPFTHLPSLVTQVEDNPFHHGQI-DQDHNTTRSPRPQLFSDPEHGEESFFYRQVLLAF : 2688
KLH1  : HSSSMAGHGVRKEINTLTTAEVDNLKDAMRAVMADHGPNGYQAIAAFHGNPPMCPMPDGKNYSCCTHGMATFPHWHRLYTKQMEDALTAHGARVGLPYWDGTTAFTALPTFVTDEEDNPFHHGHIDYLGVDTTRSPRDKLFNDPERGSESFFYRQVLLAL : 2670
OdHG  : HKKTVGDAIIRNNVNSLTPSDIKELRDAMAKVQADTSDNGYQKIASYHGIPLSCHYENGTAYACCQHGMVTFPNWHRLLTKQMEDALVAKGSHVGIPYWDWTTTFANLPVLVTEEKDNSFHHAHIDVANTDTTRSPRAQLFDDPEKGDKSFFYRQIALAL : 2677
NpH   : ERRISGGPIIRKNINKLTSSEIHELREAMAAVQADHSSNGYQAIAAYHGLPLQCK-----PYACCQHGMPTFPHWHRLYVKQMEDTLHTHGSHLGIPYWDWTEAFEYLPILVTETEHNPFHHAKIEFINETTSRNPRPQLFTDQRYGETSFFYRQILLAL : 2682

                 *      2740         *      2760         *      2780         *      2800         *      2820         *      2840         *      2860         *      2880
AcH1  : EQTDYCDFEVQYEITHNAIHSWTGGRSPYGMSTLEFTAYDPLFLLHHSNADRQFAIWQALQKFRGLPYNNANCAIQKLREPMKPFSNLDNINPTTRANARAIDAFDYDRFNYQYDNLNFHGLTISELNDLLEKRKEEDRVFAEFLLAGFGGSGDVVFNLC : 2849
AcH2  : EQTDYCDFEVQYEITHNAIHSWTGGRSPYGMSTLEFTAYDPLFLLHHSNVDRQFAIWQALQKFRGLPYNNANCAIQKLREPMKPFSNSDNINPTTRANARATDAFDYDRFNYQYDNLNFHGLTISELNDLLEKRKEEDRIFAEFLLAGFGGSADVIFNLC : 2839
LsH1  : EQTDYCNFEVQFEITHNAIHSWTGGSSQYGMSSLEYTAYDPLFLLHHSNVDRQFAIWQALQKFRGLPYNSANCAIQHLRQPMRPFSDENNLNAITRANSRAIDAFDYDRLNYQYDNLNFHGLTISQLNDLLEKRKEEDRIFAEFLLRGFSASADVIFKLC : 2851
LsH2  : EQTDYCDFEVQFEITHNAIHSWTGGSSPYGMSTLEYTAYDPLFLLHHSNVDRQFAIWQALQKFRGLPYNSANCAIQHLRQPMRPFSDESNINAITRANSRAIDAFDYDRFNYQYDNLNFHGLTISQLNDLLEKRIEEDRIFAEFLLIGFGASADVTFQLC : 2847
HpHaD : EQTDYCDFEVQYEISHNAIHSWTGGQSPYGMSTLEYTAYDPLFLLHHSNVDRQFAIWQALQKFRGLPYNSANCAIQLLHQPMRPFSDADNVNPVTRTNSRARDVFNYDRLNYQYDDLNFHGLSISELNDVLERRKEKARIFAEFLLHGIGASADVTFDLC : 2845
CaHaD : EQTDYCDFEVQYEISHNAIHSWTGGKSPYGMSTLEYTAYDPLFLLHHSNVDRQFAIWQALQKFRGLPYNSANCAIQLLHQPMRPFSDSDNINPVTRTNSRARDVFNYDRLNYQYDDLNFHGLSISELNDVLERRKEKARIFAEFLLHGLGASADITFDLC : 2843
HpHaN : EQTDYCDFEVQFEITHNAIHSWVGGQSPYGLSTLEYTAYDPLFLLHHSNVDRQFSIWQALQKYRGLPYNTANCAIQDLRRPLRPFSDPANVNPTTRANSRAIDAFNADSLHYQYDNLNFHGHSIAELEEILQHRQEEDRVFAEFLLHGLKTSADVSFDLC : 2848
CaHaN : EQTDYCDFEVQFEITHNAIHSWVGGQSPYGLSTLEYTAYDPLFLLHHSNVDRQFAIWQALQKHRGLPYNTANCAIQELRRPLRPFSDKENVNPTTRANSRAIDAFSSDRLHYQYDNLDFHGHSIVELEEILQHRQEEDRVFAEFLLHGLKTSADVTFDLC : 2849
HpHb  : EQTDYCDFEVQFEFAHNAIHSWTGGRSPYGMSTLEYTAYDPLFLLHHSNVDRQFAIWQALQKFRGLPYNSANCAIQLLHQPMRPFSDSDNVNPTTRAHSTASEAFNYEQLHYHYDNLNFHGDTISQLVNVIDERKSHDRIFAEFLLHSIGTSADVTFELC : 2846
CaHb  : EQTDYCDFEVQFEFAHNAIHSWIGGQSPYSMSTLEYTAYDPLFLLHHSNVDRQFAIWQALQKFRGLPYNSANCAIQLLHQPMRPFSDADNVNPTTRSHSTASEAFNYEQLHYQYDNLNFLGHTISELVDIIEERKSHDRIFAEFLLHSIGTSADVTFELC : 2848
KLH1  : EQTDFCQFEVQFEITHNAIHSWTGGLTPYGMSTLEYTTYDPLFWLHHANTDRIWAIWQALQEYRGLPYDHANCEIQAMKRPLRPFSDPINHNAFTHSNAKPTDVFEYSRFNFQYDNLRFHGMTIKKLEHELEKQKEEDRTFAAFLLHGIKKSADVSFDVC : 2830
OdHG  : EQTDFCDFEIQFEIGHNAIHSWVGGSSPYGMSTLHYTSYDPLFYLHHSNTDRIWSVWQALQKYRGLPYNTANCEINKLVKPLKPFNLDTNPNAVTKAHSTGATSFDYHKLGYDYDNLNFHGMTIPELEEHLKEIQHEDRVFAGFLLRTIGQSADVNFDVC : 2837
NpH   : EQTDFCDFEIQFEVGHNAIHSWVGGSTQYGMSTLHFTSYDPLFYIHHSNTDRIWAIWQALQRYRGLPYNSANCEIHLLKKPLEPFSFESNPNHETHNHSTGNAVFDYHQLGYEYDTLEFHGHNIPQLAVYLDSYHEHDRVFAGFLLHGIKRSADVNFDIC : 2842

                 *      2900         *      2920         *      2940         *      2960         *      2980         *      3000         *      3020         *      3040
AcH1  : SEE-ECAFAGTFAVLGGAIEMPWAFDRLFKYDVTNVFRKLNLRPDDVYHFEVKIVAVNGTELYPGLIRPPSVQFVPGVKGYYERVAAKTAKSSASLLRKDVNDLTLAEASNLRDALYKLQQDQGPNGFEAIAGYHGAPFKCPANGEDKYACCAHGMPVFP : 3008
AcH2  : SET-ECTFAGTFAVLGGAIEMPWAFDRLFKYDVTNVFMKLNLRPDDVYHFEVEIVAVNGTHLDSGLIRPPSVQFVPGVKGYYERVAAKTAKSSANLLRKDVNDLTLAEVSNLRDALYKMQQDQGPNGFEAIAGYHGAPFKCPAEGADKYACCAHGMPIFP : 2998
LsH1  : DEKGHCEFAGTFAVLGGLLEMPWAFDRLFKYDVTNVFNKLNLRTDSDYHFDVEIKAINGTVLDSNLIRSPSVQFVPGVKRFYEKVAEKTVVRDDKLVRKNVNQLTLDEAANLRNALNQLQNDQGPNGFEAIAGFHGAPFRCPATGDDKYACCVHGMPVFP : 3011
LsH2  : DAENHCAFAGTFAVLGGPLEMPWALDRLFKYDVTNVFNQLHLKPDSKYHFEVKIVAVNGTELDSTIIRPPTVQFVPGVKRYYEKLAERTVHREDKLTRKDVNTLTLAEATNLRNALSQLQQDAGLNGFEAIAGFHGAPFKCPATGTDKYACCVHGMPIFP : 3007
HpHaD : DSHDHCEFAGTFAILGGPLEHPWAFDRLFKYDVTDVFSKLHLRPDSEYHFNIHIVSVNGTELDSHLIRSPTVQFVPGVKDYYEKIAQKTEAQEDVLLRKNINELSLEESANLRSALNKLQQDQGPNGFEAIAGFHGAPFKCPQDGTDKYACCVHGMSVFP : 3005
CaHaD : DSHDHCEFAGTFAVLGGPLEHPWAFDRLFKYDVTDVFSKLHLRPDSEFHFNIHIVAVNGTELDSHLIRSPTVQFVPGVKDYYEKIAQQIEVHDDVLLRKNINELSLEESANLRSALNKLQQDQGPNGFEAIAGFHGAPFKCPETGADKYACCVHGMPVFP : 3003
HpHaN : DERGHCEFAGTFAILGGYLEMDWEFDRLFRYDVTNVFNKLHLQPDSDYHFVDHITAVNGTELDSQLIRPPSVHFVPGVKKP-LGVAAASGPGSGVLLRKNVNQLSQDEAVSLRDALYQLQQDQGLGGFEAIAGFHGAPFLCPENGDKKYACCVHGMPTFP : 3007
CaHaN : DERNHCEFAGTFAILGGDLEMDWEFDRLFKYDVTNVFNKLHLQPDSAYHFVDHITAVNGTELDSNLIRPPSVHFVPGVKKP-HGVAAASGPASGVLVRKNINQLSQDEAVSLRDALYQLQQDQGLGGFEAIAGFHGAPFLCPEHGAEKYACCVHGMPSFP : 3008
HpHb  : DEHNHCEFAGTFAILGGPLEMAWSTDRLFRYDVTDVFEKLHLQADSEYHFVDHIVAVNGTELDSHLIKPPSVRFVPGTKVP-QAEQTATTYQSSNLVRKSVNSLTLGEASNLKQALRELQADHGPGGFEAIAGFHGYPFLCPEKGDTKYACCVHGMPTFP : 3005
CaHb  : DERNRCAFAGTFAILGGPLEMGWANDRLFKYDVTDVFQKLHLQADSEYHFVDHIVAVNGTELDSHLIHPPSVRFVPGTRVI-QPEQEEATSQSANLIRKSVNSLTLGEASNLKQALRKLQADQGPGGFEAIAGFHGAPFLCPEKGDIKYACCVHGMATFP : 3007
KLH1  : NHDGECHFAGTFAILGGEHEMPWSFDRLFRYDITQVLKQMHLEYDSDFTFHMRIIDTSGKQLPSDLIKMPTVEHSPG--GK-HHEKHHEDHHEDILVRKNIHSLSHHEAEELRDALYKLQNDESHGGYEHIAGFHGYPNLCPEKGDEKYPCCVHGMSIFP : 2987
OdHG  : TKDGECTFGGTFCILGGEHEMFWAFDRLFKYDITTSLKHLRLDAHDDFDIKVTIKGIDGHVLSNKYLSPPTVFLAP-AK-TTH----------------------------------------------------------------------------- : 2913
NpH   : SLNGDCTNAGTFALLGGEHEMPWAFDRLFKYDITKPLQNLRLHSTDDFSINIHIVAINGEELDSHLIPTPSIIFSPG-RGAQH----------------------------------------------------------------------------- : 2919

                 *      3060         *      3080         *      3100         *      3120         *      3140         *      3160         *      3180         *      3200
AcH1  : HWHRLHTVQFEQALKEHGALVGVPYWDWTAPINALPSLIGDSSNHNPFYKYHISFVNQDTTRDIQDSLFNPRTINGYNYLYYLALSTLEEDSFCDFEIQYEILHNEIHGLIGGHGTYSMSTLDYSAFDPLFMIHHSSIDRIWAIWQQLQKLRRKPFNSAR : 3168
AcH2  : HWHRLHTVQFEQSLKEHGALVGVPYWDWTAPINALPSLIGDSSNHNPFYKSHISFVNKDTTRDIQDSLFNPRTINGYNYLYYLALSTLEEDNFCDFEIQYEVLHNEIHGLIGGHGTYSMSTLDYSAFDPLFMIHHSSIDRIWAIWQQLQKLRRKPFNSAR : 3158
LsH1  : HWHRLFTVQIEQALKSNGALIGVPYWDWTTPVKSLPSFFGDDADHNPFASFRIAAANEKTTRQVQSELYSERKVHGFPYLFYLALTTLEEDNYCDFEVQFEVLHNEIHADIGGSGTYSMATLDYSAFDPFFMIHHSSIDRIWVIWQELQKLRHKPFNGAS : 3171
LsH2  : HWHRLLTVQFEQALKAHGASVGVPYWDWTAPVRSLPSLFGDSADFNPFYSYFISFVGKKTTRDVKDELFNPPTINGFNQLYYLALSTLEEDNYCDFEVQYEILHNQIHALIGGTGTYSMATLDYSAFDPFFMIHHSSIDRIWVIWQELQKIRKKPFNAAH : 3167
HpHaD : HWHRLLTVQFEQALKAHGAKEGVPYWDWTAPIGKIPSLFGDSADYNPFYSYTISFNNQRTTRDIQSELYNPHQINGYNYLYYLALSTLEEDNFCDFEVQYEVLHNEIHALIGGNGTYSMATLDFSAFDPFFMIHHSSLDRIWIIWQELQKLRHKPFNYAH : 3165
CaHaD : HWHRLLTVQFEQALKYHGAKEGVPYWDWTAPIGKIPSLFGDSADYNPFYSYTISFNNQRTTRDIQGELYNPHTINGFNYIYYLALSTLEEDNFCDFEVQYEVLHNEIHALIGGNGTYSMATLDFSAFDPFFMIHHSSLDRIWIIWQELQKLRHKPFNYAQ : 3163
HpHaN : HWHRLFTVQFEQALKLHGSTTGIPYWDWTSPGNELPLFLADTDNDNPFSSYTISFVGQRTSRNPLGALFSTNTSAGTSLLYQLTLDALEEDDYCHFEIMLEFLHNRIHFLIGGTETYSMSTLDYSAFDPIFMIVHSGMDRLWVLWQELQKLRRKPFVAIE : 3167
CaHaN : HFHRLFTVQFEQSLKQHGSTTGIPYWDWTSPGNELPLFLADTDSDNPFSGYTISFAGQRTSRNPLEALFSTNTSAGTSLLYQLTLDALEEDDYCQFEIMLEFFHNRIHFLIGGSETYSMSTLDYSAFDPIFMIVHSGMDRLWVLWQELQKLRRKPFVAIE : 3168
HpHb  : HWHRLFTVQFEQALKQHGSIVGIPYWDWTAPGRALPPFLTDVSHDNPFSSYTITSAGKTTTRSPLEALFSANTSRGHTILYDLTLDALEEEDYCHFEASLEFLHNRIHFFIGGTGTYSMSTLDYSAFDPLFMVVHSGMDRIWDLWEQLQKLRHKSYTPVD : 3165
CaHb  : HWHRLFTVQFEQALKQHGSVVGIPYWDWTAPGRALPPFLTDASHDNPFSSYTIRSVGQTTTRSPLQALFSANTTQGHTILYDLTLNALEEEDYCHFETSLEFLHNRVHFFIGGSGTYSMSTLDYSAFDPLFMIVHSGMDRIWDLWEGLQKLRHKPYKPVS : 3167
KLH1  : HWHRLHTIQFERALKKHGSHLGIPYWDWTQTISSLPTFFADSGNNNPFFKYHIRSINQDTVRDVNEAIFQQTKFGEFSSIFYLALQALEEDNYCDFEVQYEILHNEVHALIGGAEKYSMSTLEYSAFDPYFMIHHASLDKIWIIWQELQKRRVKPAHAGS : 3147
OdHG  : ---------------------------------------------------------------------------------------------------------------------------------------------------------------- :    -
NpH   : ---------------------------------------------------------------------------------------------------------------------------------------------------------------- :    -
```

```
                  *       3220         *       3240         *       3260         *       3280         *       3300         *       3320         *       3340         *       3360
AcH1   : CGGAIMEEPLQPFSYSQINTNDFTRMNSQPSKVFDYAHLGYEFDNLELNGHDVNDLNNIINRLRDQDRVYLAFNNCGKQGSFAFDVHFETAENNVVP-LGRFYVLGGEREMPWCFERVFKYDVTEVLQANNVDIHKPVKIGGQLFRYDGEHVQNFTTYYA : 3327
AcH2   : CGGAIMEEPLQPFSYSQVNTNDFTRMNSLPSKVFDYAHLGYEFDDLDLNGHSVEDLNGIIGSFRDQNRIYIAFNNYGRQNSFTADIYLMMTGDKKVN-VGRFYMLGGEREMPWSFERLFKYDITDIVRANHVDISKPVK--------------------- : 3296
LsH1   : CAGHIMERPLQPFSYPEVNKNEFTRLNSVPNVVFDSERLGYKYDKLELNGNSVEEINNIIKNLHHQERIFVGILAFGHQKSLTIHISLINDNDEAFD-GGNIHILGGEKEMPWAYERIMKLDVTEAIRKGKISTDHAVKARFTSTDYQGNLNHQD-TDYA : 3329
LsH2   : CGGHLMETPLQPFSYPEVNPNDLTRLNAVPNLVFDSERFGYEFDKLELNGHDVSELNGIIQRLRGNTRYFLGFVLYGVRQTVGVEVTLIS-NKKRYP-AGNFYVLGGEKEMPWAYERIAKYDVTDVFHKAKLSDDKPVVGEFYSSLYNGQANNNV-TEEV : 3324
HpHaD  : CGGHVLDDPLHPFSFGEINKNDLTRLNSQPSSVFDYTHFGYEFDKLELNGHDVQGIDDIIHNLRHGNRVYLGFVLFGQQSSLEYKIDLIDDAGQAHT-AGSFHLLGGEREMPWAYERLFKYDVTDVAKQFGITTDHPIKVKVTSTYYNGEPHQEY-TDEI : 3323
CaHaD  : CGGHVLDDPLHPFSFGDINKNDLTRLNSLPSSVFDYTHFGYEFDKLELNGHDVQGIDNVIHNLRHGNRVYLGFVLFGQQSSLEYKIDLIDDEGQAHT-AGSFHLLGGEREMPWAYERLFKYDVTDVAKKFGITTDHPIKVKVTSTYYNGEPHQEY-TDEI : 3321
HpHaN  : CGEKSLHEPLHPFDY-DINTIALTREHAVPDTLFDHHLLGYEYDTTEISEHDAAEVLEIIRRRHSETRVYLGGAAYGHGQSFRTAAWVLNDAGEEFD-AGTNFLLGSSKEMPWANEILWKFDITTAVHQAGVSTDKNIKFHFKIETYNGTLFASE-SAYA : 3324
CaHaN  : CGEKSLHEPLHPFDY-DINTISLTRENAVPDSIFDHHHLQYDYDTTEISGHDAAEVLEIIRRRHSETRVYLGGAAQGYGESYRTETWVLNDAGEKFS-VGTNFLLGSAKEMPWSNEILWKFDITNAVRQAGVSTDKNIKFHFIAETYNGTLHHDQ-VAYG : 3325
HpHb   : CGGHTFDKPLHPFDFPAINTIALTRQNAIPELLFEHDRLGYEYDNLRISGYDVEQVNEILKKRHAETRIYYSEAAAGYGVSVSYEVWVLDEEGHEFP-ADTGYILGSSKEMPWTYEILFKYDITDAIHKANVSLDHPVKFKWRTVNYDGTLIEEA-SEYG : 3323
CaHb   : CAGHKFDKPLHPFDFPAINTIALTRQNAIPEQLFEHDNLGYEYDNLRISGYDVGQVNEILKKRHAETRIYYSEAASGFGVSVSYEVWVLDDEGHEIP-ADTGFVLGSSKEMPWTYEILFKYDITDAFNKANLSLDHPVKFKWRMVNYDGTFLEEA-SEYG : 3325
KLH1   : CAGDIMHVPLHPFNYESVNNDDFTRENSLPNAVVDSHRFNYKYDNLNLHGHNIEELEEVLRSLRLKSRVFAGFVLSGIRTTAVVKVYIKSGTDSDDEYAGSFVILGGAKEMPWAYERLYRFDITETVHNLNLTDDHVKFRFDLKKYDHTELDASV-LPAP : 3306
OdHG   : ---------------------------------------------------------------------------------------------------------------------------------------------------------------- : 2918
NpH    : ---------------------------------------------------------------------------------------------------------------------------------------------------------------- : 2924

                  *       3380         *       3400         *       3420         *       3440         *       3460
AcH1   : TFIERPAGVDYDIINIPVMRN-SSINPKTVVKKGTRVRFV--TDGVETPMENLGSFTNFHFCNIPPFSYHSFPFGEVHALSPGDYFFVNRDVDLCNSGR-KLQITVADE : 3432
AcH2   : ------------------------------------------------------------------------------------------------------------- :    -
LsH1   : IIVERHAEQDYDVVEIPVGRK-YPLPPKIVLKKGSRVKFYPGSDTFNSPLENLGSYTSFSKCSIPPFSYQSYALGVVHTLQPGDYFFVPKDKALCEAGK-RIQITVEDE : 3436
LsH2   : IVVEKPSDVNYDIVIIPISTG-NIIPPKIILKKGSRVKFV--SSNISVPMEELGSYTNLQKCKVPPFSFHSYPFGVVHTLAPGDYFFVPKSVQLCEAGR-RIQITVEDE : 3429
HpHaD  : VAVERHADTDYDIVVIPVSKD-NTLVPKIVVKKGTRIEFV--TSDLTDPLEDLGSYTTMHKCKIPPFSYNSYAFNTVYKLSPGDYFFVPKNVDLCNAGR-RIQITVEDD : 3428
CaHaD  : VAVERHADTDYDILVIPVSKD-NTLVPKIVVKKGTRIEFV--TSDLTDPLEDLGSYTTMHKCKIPPFSYNSYAFNTVHKLSPGDYFFVPKNAELCNAGR-RIQITVEDD : 3426
HpHaN  : IFLVREAYTDYLTLNIPVGVG-YPLPSKLVVPKKTHVRFLPVSEDYNIPIENLVSYTNYNKCSLPPFAFDSYAVNVVHALQPGDYYFTSFNKEYCTQDK-KIYIHVQDH : 3431
CaHaN  : IFVIREAYTDYLTLNIPVGGN-LPLPSKLVVPKKTHVRFLPVSEDYNVPIENLVSYTNYNKCSLPPFAFDSYAVNVVHALQPGDYYFTSFSKEHCTKGN-KIYIHVQDH : 3432
HpHb   : IVVVREAKTDYLTLIIPIGAEKYPLSPKLIVPKDTHVKFLAVNESYNYPIENLVSLTSLNRCTIPRFPFDQYNLGQVYALKPGDYYFTSSNEALCKSNK-RIYIVVEDH : 3431
CaHb   : IVVVREANTDYLTLIIPIGAEKYQLSPKLIVPKDTHVKFLAVNETYNYPIENLVSLTSLNKCTIPRFPFDQYNLGQVYALTPGDYYFTSSNETLCKSNK-RIYIVVEDH : 3433
KLH1   : IIVRRPNNAVFDIIEIPIGKD-VNLPPKVVVKRGTKIMFMSVDEAVTTPMLNLGSYTAMFKCKVPPFSFHAFELGKMYSVESGDYFMTASTTELCNDNNLRIHVHVDDE : 3414
OdHG   : ------------------------------------------------------------------------------------------------------------- :    -
NpH    : ------------------------------------------------------------------------------------------------------------- :    -
```
