# Supplementary material for: The evolution of hemocyanin genes in Tectipleura: a multitude of conserved introns in highly diverse gastropods
Source: BMC Ecol Evol. 2021 Mar 4;21:36. doi: 10.1186/s12862-021-01763-3 (PMC7931591; doi:10.1186/s12862-021-01763-3)
Supplement: Supplementary file 6 — Additional file 6: Figure S4. Splice site comparison of functional units. [file 12862_2021_1763_MOESM6_ESM.pdf]

Additional file 6

Figure S4: Splice site comparison of functional units. Amino acid sequences of functional units included in this multiple sequence alignment come from hemocyanins of Tectipleura (including hemocyanin-like proteins), Lepetellida and Cephalopoda (abbreviations see below). Colors indicate conservation scores: Red/blue: conservation of at least 95%/80% over all FUs; grey: FU-specific conservation of 95% (e.g. within FU-a, FU-b, …); yellow: splice site positions of internal introns (amino acids which are coded by the first nucleotides at the beginning of new exons are highlighted). "X" symbolizes not known amino acids. Signal peptides as well as tail extensions of FU-h are not included. Included hemocyanins: AcH1+2 (*Aplysia californica*), LsH1+2 (*Lymnaea stagnalis*), BgHl1+2 (*Biomphalaria glabrata* hemocyanin like proteins), HpHaD + HpHaN + HpHb (*Helix pomatia* alphaD, alphaN, beta), CaHaD CaHaN CaHb (*Cornu aspersum* alphaD, alphaN, beta); HtH1+2 (*Haliotis tubercularа*), KLH1+2 (*Megathura crenulata*), OdHG + OdHA (*Octopus vulgaris*), NpH (*Nautilus pompilius*).

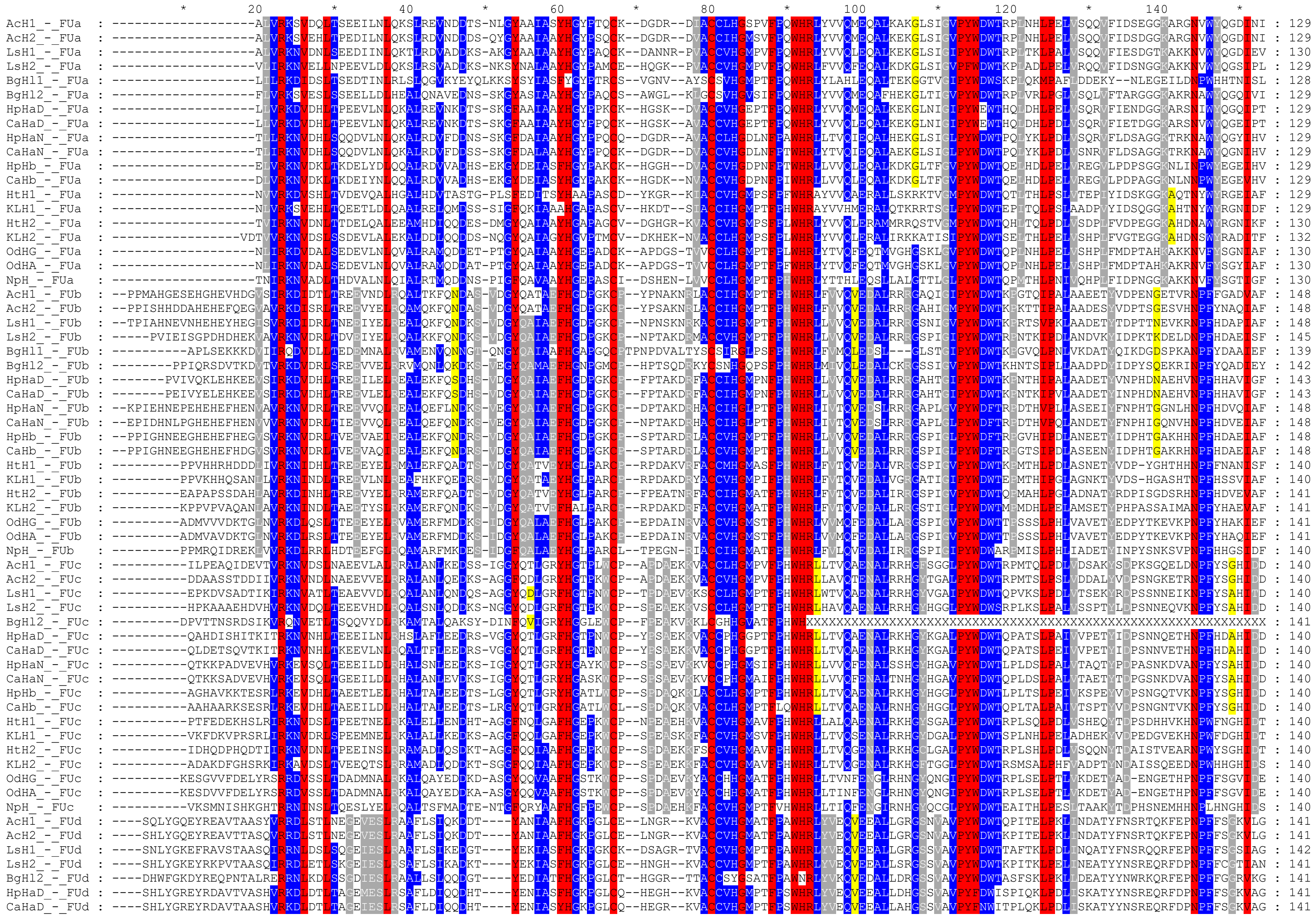
AcH1_-_FUa : 129
AcH2_-_FUa : 129
LsH1_-_FUa : 130
LsH2_-_FUa : 129
BgH11_-_FUa : 128
BgH12_-_FUa : 129
HpHaD_-_FUa : 129
CaHaD_-_FUa : 129
HpHaN_-_FUa : 129
CaHaN_-_FUa : 129
HpHb_-_FUa : 129
CaHb_-_FUa : 129
HtH1_-_FUa : 129
KLH1_-_FUa : 129
HtH2_-_FUa : 130
KLH2_-_FUa : 132
OdHG_-_FUa : 130
OdHA_-_FUa : 130
NpH_-_FUa : 130
AcH1_-_FUb : 148
AcH2_-_FUb : 148
LsH1_-_FUb : 148
LsH2_-_FUb : 145
BgH11_-_FUb : 139
BgH12_-_FUb : 142
HpHaD_-_FUb : 143
CaHaD_-_FUb : 143
HpHaN_-_FUb : 148
CaHaN_-_FUb : 148
HpHb_-_FUb : 148
CaHb_-_FUb : 148
HtH1_-_FUb : 140
KLH1_-_FUb : 140
HtH2_-_FUb : 141
KLH2_-_FUb : 141
OdHG_-_FUb : 141
OdHA_-_FUb : 141
NpH_-_FUb : 140
AcH1_-_FUc : 140
AcH2_-_FUc : 140
LsH1_-_FUc : 140
LsH2_-_FUc : 140
BgH12_-_FUc : 141
HpHaD_-_FUc : 140
CaHaD_-_FUc : 140
HpHaN_-_FUc : 140
CaHaN_-_FUc : 140
HpHb_-_FUc : 140
CaHb_-_FUc : 140
HtH1_-_FUc : 140
KLH1_-_FUc : 140
HtH2_-_FUc : 140
KLH2_-_FUc : 140
OdHG_-_FUc : 140
OdHA_-_FUc : 140
NpH_-_FUc : 140
AcH1_-_FUd : 141
AcH2_-_FUd : 141
LsH1_-_FUd : 142
LsH2_-_FUd : 141
BgH12_-_FUd : 141
HpHaD_-_FUd : 141
CaHaD_-_FUd : 141

```
HpHaN_-_FUd  : ----SHIYGQEYRPVVAAASQVRRNLESLSEGEIESLRAAFLAIQNDHS----YEAIASFHGKPGLCE--HEGR--KVACCVHGDPTFPSWHRLYVELVEHALLSHGSSVAVPYWDWISPIKKLPKLISKSTYYNSRQQRFDPNPFFSGRIAG : 141
CaHaN_-_FUd  : ----SHLYGQEYRPVVAAASQVRRNLESLSEGEIESLRAAFLAIQNDHS----YEGIASYHGKPGLCE--HQGR--KVACCVHGNPTFPSWHRLYVELVEHALLSHGSSVAVPYWDWISPIKKLPKLISKSTYYNSRQQRFDPNPFFSGRIAG : 141
HpHb_-_FUd   : ----SHIYGQEYRPLVTAGSHVRHNLEHLSAGEVESLRSAFLAIQEDHS----YEDIAAYHGKPGLCE--FEGR--KAACCVHGSAAFPSWHRLYVEQVEHALLAQGSSVSIPYWDWAVPIRSLPKLISEATYFNSRQQRFDANPFFSGRISG : 141
CaHb_-_FUd   : ----SHIYGQEYRPQVTVGSHVRFNLEDLSAGEIESLRSAFLAIQEDHT----YENIAAYHGKPGLCE--YEGH--KAACCVHGSAAFPPWHRLYVEQVEHALLARGSSVSIPYWDWAVPIRSLPKLISEATYFNSRQQRFDANPFFSGRIVG : 141
HtH1_-_FUd   : ----GTRDRDNYVEEVTGASHIRKNLNDLNTGEMESLRAAFLHIQDDGT----YESIAQYHGKPGKCQ--LNDH--NIACCVHGMPTFPQWHRLYVVQVENALLNRGSGVAVPYWEWTAPIDHLPHFIDDATYFNSRQQRYDPNPFFRGKVTF : 141
KLH1_-_FUd   : ----GHHEGEVYQAEVTSANRIRKNIENLSLGELESLRAAFLEIENDGT----YESIAKFHGSPGLCQ--LNGN--PISCCVHGMPTFPHWHRLYVVVVENALLKKGSSVAVPYWDWTKRIEHLPHLISDATYYNSRQHHYETNPFHHGKITH : 141
HtH2_-_FUd   : -------QADEYDEVVTAASHIRKNLKDLSKGEVESLRSAFLQLQNDGV----YENIAKFHGKPGLCD--DNGR--KVACCVHGMPTFPQWHRLYVLQVENALLERGSAVSVPYWDWTETFTELPSLIAEATYFNSRQQTFDPNPFFRGKISF : 138
KLH2_-_FUd   : ----GSHQADEYREAVTSASHIRKNIRDLSEGEIESIRSAFLQIQKEGI----YENIAKFHGKPGLCE--HDGH--PVACCVHGMPTFPHWHRLYVLQVENALLERGSAVAVPYWDWTEKADSLPSLINDATYFNSRSQTFDPNPFFRGHIAF : 141
OdHG_-_FUd   : ---GNFGEAGIWVEPVTSANRIRKNLNALTDGDMESLRKAFKDMTTDGR----YEEIASFHGLPAQCP--NKDGSKVYTCCIHGMPTFPHWHRLYVALVENELLARGSGVAVPYWDWVQPFDHLPALVNRATYYNSRTLLVEPNPFFKGKISF : 144
OdHA_-_FUd   : ---GNFGEAGIWVEPVTSANRIRKNLNALTEGDMESLRNAFKDMTTDGR----YEEIASFHGLPAQCP--NKDGSKVYTCCIHGMPTFPHWHRLYVALVENELLARGSGVAVPYWDWVQPFDHLPALVNRATYYNSRTLLVEPNPFFKGKISF : 144
NpH_-_FUd    : ---GTYGTQTKWREPVTSASRIRKDLNTLTDGELESLRNAFLRLQEEGA----YEPIAAFHGVPAKCR--GEDGN-IYTCCVHGMPTFPHWHRLYVIQVEHALRERGSGVSVPYWDWTKRFNHLPALMDESTFYNSRKNEMNTNPFHRGSISF : 143
AcH1_-_FUe   : -------NERQEADGQVGNYLVRKNIASLSPQEELSLLKAMAALQADSS-ADGYQSIASFHAIPPLCP--SPSASKRYACCLHGMATFPQWHRLYTVQVEDALRRHGSLVGIPYWDWSRQTDHLPGLLANPTYTDVYTGQTIDNPWYKGKIEF : 143
AcH2_-_FUe   : -------NERQESDGQLSSYQVRKSIDALSPQEELSLLKAMAALQADSS-ADGYQSIASFHAIPPLCP--SPSASKRYACCLHGMATFPQWHRLYTVQVEDALRRHGSLVGIPYWDWSRQTDHLPGLLANPTYTDVYTGQTIDNPWYKARIEF : 143
LsH1_-_FUe   : -------GERQKSSGEVTNYLVRKEINSLSPREVQSLYSAMEALQADSS-ADGWQSIASFHAIPPLCP--SPSAENRYACCLHGMATFPQWHRLYTVQVEDALKRHGALVGIPYWDWSRVSDSLPHFIDDENFVDPVTGDQKANPWKKARIEF : 143
LsH2_-_FUe   : -------SERQEAHGAVSSFVVRKNVDALSQREALSLYNAMRALQADSS-ADGFQAIASFHALPPLCP--SPAAINRYACCLHGMATFPQWHRLYTVQVEDALRRHGSLVGIPYWDWTRQSKSLPEFFSDPTYPDPDGGDPVDNPWYKANIEF : 143
BgHl2_-_FUe  : -------EKSYTSESHVINVRARKEVRSLNPREILSLYHAMASLQQDNS-PDGWQALAAFHALPSLCP--NPRAEKRYVCSPIGTATFLPWHRLYTVQVEDALRRHGALVGLPYWDWTREGSELPEFLEDDNYHDPLTGKTLPNPFVHSKIGS : 143
HpHaD_-_FUe  : -------KERQAEDGDVKDIVVRKNVDALSPRETLSLIHALEALQADSS-ADGYQSIAAFHAVPPLCP--SPSASTRYACCLHGMSTFPQWHRLYTVQVEDALRRHGSVVGIPYWDWTRASQSLPHFLSDNNYTDPYTKEVHDNPWHGASIDF : 143
CaHaD_-_FUe  : -------RERQTEDGDVKHVVVRKNVDALSPRETLALIHALEALQADSS-ADGYQSIAAFHAVPPLCP--SPSASKRYACCLHGMSTFPQWHRLYTVQVEDALRRHGSGVGIPYWDWTRASQSLPGFLSAENYTDPYTKEVHENPWHGASIDF : 143
HpHaN_-_FUe  : -------DEVEDQHGSTSTYLVRKNVESLSPLEGYYLAEALIALKKDTS-ADGYQSIATFHAIPPLCP--SPTASRRYACCIHGGTSFLQWHRLYTVQFEDALKRHGSPIGVPYWDWTRFSKELPRTFTYANYSDPFTNLWTLNPFYSGRVEF : 143
CaHaN_-_FUe  : -------DEEQDQLGATTTYLVRKNVESLNPLEGYHLADALIALKKDTS-ADGFQSIATFHAIPPLCP--SPTASLRYACCIHGGTSFLQWHRLYTVQFEDALKRHGSPIGVPYWDWTRFSTELPRTFTFTNYSDPFTNVWTLNPFYKGRVEF : 143
HpHb_-_FUe   : -------DEAEHPHGDRAPLLVRKNVESLSPLEKYHLVKALTSLNADGS-ADGFQSIATFHAIPPLCP--SPTASVRHACCIHGGAAFLQWHRLYTVQFEDALRRHGSHVGLPYWDWVRITDRLPSIFTVESQTDPSTNVSVPNPFATGRVEF : 143
CaHb_-_FUe   : -------DDVELPHGGRAPSLVRKNVESLSPLEKYHLVKALTSLNADGS-ADGFQSIATFHAIPPLCP--SPTASVRHACCIHGGAAFLPWHRLYTVQFEDALRRHGSHVGLPYWDWVRISDRLPSILTVESQTDPSTNVSVPNPFATGRVEF : 143
HtH1_-_FUe   : -------DTHILDHDHEEEILVRKNIIDLSPRERVSLVKALQRMKNDRS-ADGYQAIASFHALPPLCP--NPSAAHRYACCVHGMATFPQWHRLYTVQVQDALRRHGSLVGIPYWDWTKPVNELPELLSSATFYHPIRNINISNPFLGADIEF : 143
KLH1_-_FUe   : -------DSAHTDDGHTEPVMIRKDITQLDKRQQLSLVKALESMKADHS-SDGFQAIASFHALPPLCP--SPAASKRFACCVHGMATFPQWHRLYTVQFQDSLRKHGAVVGLPYWDWTLPRSELPELLTVSTIHDPETGRDIPNPFIGSKIEF : 143
HtH2_-_FUe   : -------SSFLRPDGHSDDILVRKEVNSLTTRETASLIHALKSMQEDHS-PDGFQAIASFHALPPLCP--SPSAAHRYACCVHGMATFPQWHRLYTVQFQDALRRHGATVGVPYWDWLRPQSHLPELVTMETYHDIWSNRDFPNPFYQANIEF : 143
KLH2_-_FUe   : -------RKNIYYDGLSQHNLVRKEVSSLTTLEKHFLRKALKNMQADDS-PDGYQAIASFHALPPLCP--SPSAAHRHACCLHGMATFPQWHRLYTVQFEDSLKRHGSIVGLPYWDWLKPQSALPDLVTQETYEHLFSHKTFPNPFLKANIEF : 143
OdHG_-_FUe   : ------DHSADIKSEEGNEYLVRKNVERLSLSEMNSLIHAFRRMQRDKS-SDGFEAIASFHALPPLCP--SPTAKHRHACCLHGMATFPHWHRLYVVQFEQALHRHGATVGVPYWDWTRPISKIPDFIASKRYSDPFTKIEDYNPFNQGQISF : 144
OdHA_-_FUe   : ------DHSADIKSEEGNEYLVRKNVERLSLSEMNSLIHAFRRMQKDKS-SDGFEAIASFHALPPLCP--SPTAKHRHACCLHGMATFPHWHRLYVVQFEQALHRHGATVGVPYWDWTRPISKIPDFIASEKYSDPFTKIEDYNPFNHGHISF : 144
NpH_-_FUe    : -----GSKNATHYQEEEHHHFVRKEVSRLSIYEIHEVVYAFRRMQRDMS-ADGFEYIASYHADPPLCP--NPKALHRYACCIHGMATFLHWHRLYVVQFEQSLHRHGSDIGFPYWDWTRPITHIPALLKDVNYTDPFTNSVVGNPFNHGHLSF : 145
AcH1_-_FUf   : ---------HDFKREHVAPNHVRRNLDTLEERDLQSLKAALRDLQHDNS-NDGWASLASFHGSPAKCS--TPANE-SVACCIHGMPTFPHWHRLFTLQVEQALRRHGSAIAIPYWDWTLPITDLPEIFTSQNYYDVWRDEVVNNPFARGYVPT : 140
AcH2_-_FUf   : ---------DDYNLEKVTPNHIRRNLDELSERDLQSLKAALRDLQLDTS-NDGWANLASFHGAPARCP--DPKNP-TVACCVHGMPTFPHWHRLYTLQVEQALRRHGSAIAIPYWDWTQPISHLPEIFTSEDFYDVWRDEVVANPFARGYIPT : 140
LsH1_-_FUf   : ---------RDYDLEKVTPNFVRHDLSTLTERDLQGLKSALRDLQLDES-ADGWASLASFHGAPSMCE--NESGA-KVACCIHGMPTFPHWHRLFTVQVERALQRHGSAVAIPYWDWTKPITALPEIFTKEDFYDAWRDEVVDNPFARGFIPT : 140
LsH2_-_FUf   : ---------SDHDLDNVTPNLIRRDLSTLSERDIQSLQAALRDLQLDQS-NDGWASLASFHGAPNRCP--DPANP-TVACCQHGMPTFPHWHRLFTVQIEQALQRHGSAIAIPYWDWTKPIDELPKIFTDEDFYDVWRDEVVANPFARGYVPS : 140
BgHl2_-_FUf  : ---------EDPHLTLVTPNLIRRDLSTLTERDFHSLRVALQNLQQDQT-DDGWARLASFHGAPACCK--DEEGN-PTACSAQGLPTFPHWHRLFTLQIEQALRRHGSAIAVPYWDWTFPTTELPSIFTEEDYYDILKDEVVPNPFVQGYIPT : 140
HpHaD_-_FUf  : ---------EDVHHVEATPNNIRRNLDTLEERDIQSLQAALRDLQRDTT-NDGWTNLASFHGAPARCP--DPSHP-TVACCVHGMPTFPHWHRLFALQIEQALHRHGSSIALPYWDWTQAIDDLPSTFTKEDYYDVWRDEVVPNPFAHGYVTS : 140
CaHaD_-_FUf  : ----------EDVHVETTPNNIRHNLNSLEERDIQSLQAALRNLQRDTT-NDGWASLASFHGAPARCP--DPDHP-KVACCVHGMPTFPHWHRLFALQIEQALHRHGSSIALPYWDWTLAIDELPSTFTKEDYYDVWRDEVVPNPFAHGYVAS : 139
HpHaN_-_FUf  : ---------HDVTLKKVTVNHIRRNLDEVEERDTQSIQRALRDLLADTG-PDGWASLASFHGAPARCP--DPDHP-TVACCQHGMPTFLHWHRLFTLQVEQAIQKHGSAIAIPYWDWTQPIKKLPDIFTKVNYYDAWSDQVLENPFAHGSIPS : 140
CaHaN_-_FUf  : ---------HDVTLKKVTVNHIRRNLDDVEDRDTQSIQTALRKLLADTG-PDGWASLASFHGAPARCP--DPDHP-TVACCQHGMFTFLHWHRLFTLQVEQAIQKHGSSIAIPYWDWTQPLKKLPDIFTRVNYYDAWSDQVLENPFAHGSIPS : 140
HpHb_-_FUf   : ---------HDLNLKKVPLNKIRRNIDSLEERDIQSLQTALHDIQEDDS-NNGWANLASFHGAPARCP--DPEHP-KVACCQHGMPTFPHWHRLFTLQVEQALQAHGSSLAIPYWDWTLHVSELPKIFTEEDYYDVWKDEVLENPFAHGYIPS : 140
CaHb_-_FUf   : ---------RDIHLRKVPLNKIRRNIDSLEERDIQSLQSALYDLQEDES-NNGWANLASFHGAPARCP--DPEHP-KVACCQHGMQTFPHWHRLFTLQVEQALRDHGSSLAIPYWDWTLHVSELPKIFTEEDYYDVWKDSVLENPFAHGYIPS : 140
HtH1_-_FUf   : ---------HKLNSRKHTPNRVRHELSSLSSRDIASLKAALTSLQHDNG-TDGYQAIAAFHGVPAQCH--EPSGR-EIACCIHGMATFPHWHRLYTLQLEQALRRHGSSVAVPYWDWTKPITELPHILTDGEYYDVWQNAVLANPFARGYVKI : 140
KLH1_-_FUf   : ---------HDISSHHLSLNKVRHDLSTLSERDIGSLKYALSSLQADTS-ADGFAAIASFHGLPAKCN--DSHNN-EVACCIHGMPTFPHWHRLYTLQFEQALRRHGSSVAVPYWDWTKPIHNIPHLFTDKEYYDVWRNKVMPNPFARGYVPS : 140
HtH2_-_FUf   : ---------RDINTRSMSPNRVRRELSDLSARDLSSLKSALRDLQEDDG-PNGYQALAAFHGLPAGCH--DSRGN-EIACCIHGMPTFPQWHRLYTLQLEMALRRHGSSVAIPYWDWTKPISELPSLFTSPEYYDPWHDAVVNNPFSKGFVKF : 140
KLH2_-_FUf   : ---------HGINVRHVGRNRIRMELSELTERDLASLKSAMRSLQADDG-VNGYQAIASFHGLPASCH--DDEGH-EIACCIHGMPVFPHWHRLYTLQMDMALLSHGSAVAIPYWDWTKPISKLPDLFTSPEYYDPWRDAVVNNPFAKGYIKS : 140
OdHG_-_FUf   : ---------NEDADIDTPLNHIRRNVESLDERDIQNLMAALTRVKEDES-DHGFQTIASYHG-STLCP--SPEEP-KYACCLHGMPVFPHWHRVYLLHFEDSMRRHGSSVATPYWDWTQPGTKLPRLLADSDYYDAWTDNVTENPFLRGYIKS : 139
OdHA_-_FUf   : ---------KQGADIDIPLNHIRRNVESLDERDIQNLMAALTRVKKDES-DHGFQTIASYHG-STLCP--SPEEP-KYACCLHGMPVFPHWHRVYLLHFEDSMRRHGSSVATPYWDWTQPGTKLPRLLADSDYYDAWTDNVTENPFLRGYIKS : 139
NpH_-_FUf    : ----------EHHEVHHPLNRIRHNINDLSERDIQSLKSALIKVKEDHS-NFGFQTITSYHGLPASCP--TPEEA-TYACCMHGMAAFPHWHRLYALQFEESLLRHGSSVALPYWDWTHKITGLPHLLSNPDYYDALHKQVTENPFLRGHIEV : 139
AcH1_-_FUg   : -----GASEEVSSSSSLAGVGVRKDVSTLSTSEIDNLREALRRVQADAG-PNGFASIAAFHGEPAGCE--LNGR--RIACCQHGMANFPQWHRLFVKQFEDALTAQGAMIGIPYWDWTTAFTALPSLVTEQE----------NNPFYNFKI-F : 132
AcH2_-_FUg   : ------GAAEDVSSTTLSGVGVRKDVSTLSAAEISNLRDALRKVQADVG-PNGFASIAAFHGEPAGCE--LNGH--PIACCQHGMANFPQWHRLFVKQFEDALTAQGAMIGIPYWDWTTAFTALPSLVTEQE----------NNPFYNFKI-F : 131
LsH1_-_FUg   : ------GVANVDITSAVAGVGVRKDVSSLTASETESLRNALRKVQEDEG-PNGFQNIASFHGSLAKCE--YQGH--PIACCLHGMANFPQWHRLYVKQWEDALTSQGSKVGIPYWDWTTAFTALPTLVTEET----------NNPFHHGKI-Y : 131
LsH2_-_FUg   : ------GSTQDAVSTSIAGVGVRKDVSTLTVSEIENIRNALKRVQEDDG-PNGFQNIASFHGSPAKCE--YQGH--PIACCLHGMANFPQWHRLYVKQWEDALTAQGAKIGIPYWDWTTAFTALPTLVSQED----------DNPFHHGKI-Y : 131
HpHaD_-_FUg  : ------GVKKDIHTTAVAGVGVRKDVTRLTVSETENLREALRRIKADNG-SNGFQSIASFHGSPPGCE--HENH--SVACCIHGMANFPQWHRLYVKQWEDALTAQGAKIGIPYWDWTTAFTELPALVTEEV----------DNPFHHGTI-Y : 131
CaHaD_-_FUg  : ------GVDKNIHTTAVAGVGVRKDVTRLTVSETENLREALRRVKADNG-SNGFQKIASFHGSPPGCV--HENH--YVACCIHGMANFPQWHRLYVKQWEDALTAQGAQIGIPYWDWTTAFTELPALVTEED----------DNPFHHGKI-Y : 131
HpHaN_-_FUg  : ------EGAEDVSSTSIAGVGVRKDVSSLSTSEIDNIRSALQQVEDDTG-PNGFQNIASFHGSPARCE--HDHH--PVACCVHGSPNFPQWHRLYVKQWEDALTAHGAKIGIPYWDWTYAFKKLPSLVTAGD----------NNPFHHG-VTH : 131
CaHaN_-_FUg  : ------EGAEDVSSTSIAGIGVRKDVSTLSTSEIYNIRSALRQVKDDAG-ANGFLNIASFHGKPARCE--HDHH--PVACCVHGSPKFPHWHRLYVKQWEDALTAHGAKIGIPYWDWTYAFKKLPSLVTAEV----------DNPFHHG-VTH : 131
HpHb_-_FUg   : ------EEAKKTKAVPVPGDSVRKNVNDLTNSDVANLRAALRDVQADDG-ANGFASIAGFHGSPAHCE--HDHH--PVACCLHGMANFPQWHRLYVKQWEDALIAHGSKNGIPYWDWTQSFTELPTLVTQVE----------DNPFHHGKIDK : 132
CaHb_-_FUg   : ------DEEEKAARVPVPGDSIRKNVNDLTDSEVANLRAALYDVQADTG-PNGFAFIASFHGYPARCE--HDNH--PVACCLHGMASFPQWHRLYVKQWEDALISHGSREGVPYWDWAQPFTHLPSLVTQVE----------DNPFHHGQIDQ : 132
HtH1_-_FUg   : ------DHHDDHQSGSIAGSGVRKDVNTLTKAETDNLREALWGVMADHG-PNGFQAIAAFHGKPALCP--MPDGH-NYSCCTHGMATFPHWHRLYTKQMEDAMRAHGSHVGLPYWDWTAAFTHLPTLVTDTD----------NNPFQHGHIDY : 133
KLH1_-_FUg   : ------DHHEDHHSSSMAGHGVRKEINTLTTAEVDNLKDAMRAVMADHG-PNGYQAIAAFHGNPPMCP--MPDGK-NYSCCTHGMATFPHWHRLYTKQMEDALTAHGARVGLPYWDGTTAFTALPTFVTDEE----------DNPFHHGHIDY : 133
HtH2_-_FUg   : -----GRAADSAHSANIAGSGVRKDVTTLTVSETENLRQALQGVIDDTG-PNGYQAIASFHGSPPMCE--MNGR--KGACCAHGMASFPHWHRLYVKQMEDALADHGSHIGIPYWDWTTAFTELPALVTDSE----------NNPFHEGRIDH : 133
KLH2_-_FUg   : ------GHTDDHGSDHIAGSGVRKDVTSLTASEIENLRHALQSVMDDDG-PNGFQAIAAYHGSPPMCH--MDGR--DVACCTHGMASFPHWHRLFVKQMEDALAAHGAHIGIPYWDWTSAFSHLPALVTDHE----------HNPFHHGHIAH : 132
OdHG_-_FUg   : ------DFKREVHKKTVGDAIIRNNVNSLTPSDIKELRDAMAKVQADTS-DNGYQKIASYHGIPLSCH--YENGT-AYACCQHGMVTFPNWHRLLTKQMEDALVAKGSHVGIPYWDWTTTFANLPVLVTEEK----------DNSFHHAHIDV : 133
OdHA_-_FUg   : ------EFTKEIEKEAVRGTIIRKNVNSLTPSDIKELRDAMAKVQADTS-DNGYQKIASYHGMPLSCH--YENGT-AYACCQHGMVTFPNWHRLLTKQMEDALVAKGSHVGIPYWDWTTTFANLPVLVTEEK----------DNSFHHAHIDV : 133
NpH_-_FUg    : -----NTKQAERERRISGGPIIRKNINKLTSSEIHELREAMAAVQADHS-SNGYQAIAAYHGLPLQCK--------PYACCQHGMPTFPHWHRLYVKQMEDTLHTHGSHLGIPYWDWTEAFEYLPILVTETE----------HNPFHHAKIEF : 129
```

```
AcH1_-_FUh  : ----GYYERVAAKTAKSSASLLRKDVNDLTLAEASNLRDALYKLQQDQG-PNGFEAIAGYHGAPFKCP--ANGED-KYACCAHGMPVFPHWHRLHTVQFEQALKEHGALVGVPYWDWTAPINALPSLIGDSS---------NHNPFYKYHISF : 136
LsH1_-_FUh  : ----RFYEKVAEKTVVRDDKLVRKNVNQLTLDEAANLRNALNQLQNDQG-PNGFEAIAGFHGAPFRCP--ATGDD-KYACCVHGMPVFPHWHRLFTVQIEQALKSNGALIGVPYWDWTTPVKSLPSFFGDDA---------DHNPFASFRIAA : 136
LsH2_-_FUh  : ----RYYEKLAERTVHREDKLTRKDVNTLTLAEATNLRNALSQLQQDAG-LNGFEAIAGFHGAPFKCP--ATGTD-KYACCVHGMPIFPHWHRLLTVQFEQALKAHGASVGVPYWDWTAPVRSLPSLFGDSA---------DFNPFYSYFISF : 136
BgHl1_-_FUh : -----------DSEIETQPALLRKNIQELTDGESNNLRDALKKLQSETS-ADNFENIAGFHGAPNRCP--PHGSD-RFACSPHGLPIFPHWHRLLTVQFEQALSRLGASWGIPYWDWTDESTALPKLFSDP----------EDNPFYRYYIQA : 128
HpHaD_-_FUh : ----DYYEKIAQKTEAQEDVLLRKNINELSLEESANLRSALNKLQQDQG-PNGFEAIAGFHGAPFKCP--QDGTD-KYACCVHGMSVFPHWHRLLTVQFEQALKAHGAKEGVPYWDWTAPIGKIPSLFGDSA---------DYNPFYSYTISF : 136
CaHaD_-_FUh : ----DYYEKIAQQIEVHDDVLLRKNINELSLEESANLRSALNKLQQDQG-PNGFEAIAGFHGAPFKCP--ETGAD-KYACCVHGMPVFPHWHRLLTVQFEQALKYHGAKEGVPYWDWTAPIGKIPSLFGDSA---------DYNPFYSYTISF : 136
HpHaN_-_FUh : -----KPLGVAAASGPGSGVLLRKNVNQLSQDEAVSLRDALYQLQQDQG-LGGFEAIAGFHGAPFLCP--ENGDK-KYACCVHGMPTFPHWHRLFTVQFEQALKLHGSTTGIPYWDWTSPGNELPLFLADTD---------NDNPFSSYTISF : 135
CaHaN_-_FUh : -----KPHGVAAASGPASGVLVRKNINQLSQDEAVSLRDALYQLQQDQG-LGGFEAIAGFHGAPFLCP--EHGAE-KYACCVHGMPSFPHFHRLFTVQFEQSLKQHGSTTGIPYWDWTSPGNELPLFLADTD---------SDNPFSGYTISF : 135
HpHb_-_FUh  : -----VPQAEQTATTYQSSNLVRKSVNSLTLGEASNLKQALRELQADHG-PGGFEAIAGFHGYPFLCP--EKGDT-KYACCVHGMPTFPHWHRLFTVQFEQALKQHGSIVGIPYWDWTAPGRALPPFLTDVS---------HDNPFSSYTITS : 135
CaHb_-_FUh  : -----VIQPEQEEATSQSANLIRKSVNSLTLGEASNLKQALRKLQADQG-PGGFEAIAGFHGAPFLCP--EKGDI-KYACCVHGMATFPHWHRLFTVQFEQALKQHGSVVGIPYWDWTAPGRALPPFLTDAS---------HDNPFSSYTIRS : 135
HtH1_-_FUh  : --HRGGKHEDEHHDDRLADVLIRKEVDFLSLQEANAIKDALYKLQNDDS-KGGFEAIAGYHGYPNMCP--ERGTD-KYPCCVHGMPVFPHWHRLHTIQMERALKNHGSPMGIPYWDWTKKMSSLPSFFGDSS---------NNNPFYKYYIRG : 138
KLH1_-_FUh  : ------KHHEKHHEDHHEDILVRKNIHSLSHHEAEELRDALYKLQNDES-HGGYEHIAGFHGYPNLCP--EKGDE-KYPCCVHGMSIFPHWHRLHTIQFERALKKHGSHLGIPYWDWTQTISSLPTFFADSG---------NNNPFFKYHIRS : 134
HtH2_-_FUh  : HRGPVEETEVTRQHTDGNAHFHRKEVDSLSLDEANNLKNALYKLQNDHS-LTGYEAISGYHGYPNLCP--EEGDD-KIPCCVHGMGIFPYWHRLLTIQLERALEHNGALLGVPYWDWNKDLSSLPAFFSDSS---------NNNPYFKYHIAG : 140
KLH2_-_FUh  : ---HHGGDTSGHDHSERHDGFFRKEVGSLSLDEANDLKNALYKLQNDQG-PNGYESIAGYHGYPFLCP--EHGED-QYACCVHGMPVFPHWHRLHTIQFERALKEHGSHLGIPYWDWTKSMIALPAFFADSS---------NSNPFYKYHIMK : 137
```

```
                    160         *         180         *         200         *         220         *         240         *         260         *         280         *         300
AcH1_-_FUa  : DGQ----VVHTARAV-DDRLFQQVA-PGENTQLFEMVLNALEYENYCQFEVQFEVAHNTIHYLVGGRHKYSMSHLEYTSYDEIFFLHHSNVDRILAVKLALLG-PGAPQKGGV----EFC-DLKNSMTPMAPFSWDSNPIALTRDHSIEPQTL : 270
AcH2_-_FUa  : DGH----VVHTARAV-DERLFQQVE-AGERTDIFEQVLNALEYENYCQFEVQFEVAHNTIHYLVGGRHTYSMSHLEYTSYDEIFFLHHSNVDRIYAVYFELLQKLRGNRRGNACVGEGELC-DIAAFQKELVPFNWDSNPFPLTKRHNKAYEVI : 275
LsH1_-_FUa  : VENSKTVIRHTARAI-DDRLFQKVEPG-QNTDIFEQVINALMYPNYCQFEVQFEIAHNTIHYLVGGRNKYSVSHLEYTSYDEIFFLHHSNVDRIYSIYEALQKEKGYTPGPGCTGKGELC-DIKHFDEPLAPFSWETNPFAVTKQFNLPREAV : 280
LsH2_-_FUa  : KTG----PRFTARAV-DDRLFQKVE-AGEHTDIFEQVLNALEYPNYCQFEVQFEVAHNTIHYLVGGRNRYSMSHLEYTSYDEIFFLHHSNVDRIYAVYFELLQRLRGYTPGPGCKGEGETC-DIKGFQKPLEPFNRASNPWPRTRNHATGFESL : 275
BgH11_-_FUa : SGV----IHATNRTV-DSRLWS--------LDLMEHIIHALEYPNYCQFVVQLEVLHSAIHFLVGGASKYSMSNIDFAAYDEIFLVHHANLDRIYEVYFALYRERGSVPGTSCETDCEIC-DIKGFQMPLEPFNRDDNPFPNTRLLATGWNMT : 267
BgH12_-_FUa : GDQ----MIRTARSV-DKRLFQKYG-PGEHTNIFEQVLNALEYKDYNQFEVQLEIAQNTIHHLVGGRNKYSMSNLDYASYDEIFFLHHANVDRIYTIYERLYGSARINSF-----------DVQTFMKPMDPFSWETNPFNITKDQSKPKSTF : 265
HpHaD_-_FUa : PEG----VKNTARAV-DPRLFQQVE-TGQYTDIFEHVLNALEYPNYCQFEVQFEVAHNTIHYLVGGRHTYSVSHLEYTSYDEIFFLHHSNVDRIYTIYETIQRSRGYTPGPGCTGEGELC-DIVGFRTPLEPFSRDSNPFALTRVHSHPYEAT : 275
CaHaD_-_FUa : PEG----VKKTARAV-DPRLFQQVV-AGQYTDIFEHVLNALEYPNYCQFEVQFEVAHNTIHYLVGGRHTYSVSHLEYTSYDEIFFLHHSNVDRIYTIYETIQRARGYTPGPGCTGEGELC-DIVGFRTPLEPFSRDSNPFALTKTHSHPYEAT : 275
HpHaN_-_FUa : GKK----TYKTARAV-DKRLYQNVT-GEEHTDIFEQVLHAFEYGY-CQFEVQFEVAHNTIHSLVGGRYPYSVSSLDYTGYDELFYLHHSNVDRLYAIYQEVQKLRGVQSGSANSPLC----DVKSYYRPLEPFRRKSNPFSLTRTYNSADKAK : 271
CaHaN_-_FUa : GAK----TYKTARAV-DKRLYQNVS-GDEHTDIFEHVLHAFEYSSYCQFEVQFEVAHSTIHSLVGGRFPYSVSSLDYTAYDEIFYLHHSNVDRLFAIYQEVQRIRGIESGSANSPIC----DVKTFYRPLEPFSRTSNPFSLTKTYNSPDKAK : 272
HpHb_-_FUa  : GDK----TYHTSRAI-DERLYQHVA-PGQHTDIFEHVLDAFEYTDFCQFEVQFEVSHNYIHSLVGGRSQYSLSSLEFTVYDEIFFLHHSNVERLFQIYTEVQKYREAKGEGGQKVIC----DLKGYFAPLEPFSRASNPFPVTRENSSPEKAL : 272
CaHb_-_FUa  : GDQ----TYHTTRAI-DERLYQHVA-PGQHTDIFEHVLDAFEYTSFCQFEVQFEVAHNYIHSLVGGRSQYSLSSLEYTVYDEIFFLHHSNVERLFQIYTAVQKHREVENEAGKKEIC----DLKGFFAPLEPFNRDSNPFPVTRENSSPDKSQ : 272
HtH1_-_FUa  : I------NKKTARAV-DDRLFEKVE-PGHYTHIMETVLDALEQDEFCKFEIQFEIAHNAIHYLVGGKFEYSMSNLEYTSYDEIFFLHHSNVDRLFAIWQRLQELRGKNPNAM---DCAHE-LAH---QQLQPFNRDSNPVQLTKDHSTPADLF : 267
KLH1_-_FUa  : L------DKKTNRAV-DDRLFEKVK-PGQHTHIMESVLDALEQDEFCKFEIQFEIAHNAIHYLVGGKHDYSMANLEYTAYDEIFFLHHSNVDRIFAIWQRLQELRNKDPKAM---DCAQE-LLH---QKMEPFSWEDNDIPLTNEHSTPADLF : 267
HtH2_-_FUa  : E------NKKTARAV-DDRLFEKVG-PGENTRIFEGILDALEQDEFCNFEIQFEIAHNAIHYLVGGRHTYSMSHLEYTSYDEIFFLHHSNTDRIFAIWQRLQVLRGKDPNTA---DCAHN-LIH---EPMEPFRRDSNPLDLTRENSKPIDSF : 268
KLH2_-_FUa  : L------NKKTSRAV-DDRLFEKVQ-PGHHTRIMEGILDALEQDEFCKFEIQFEIAHNAIHYLVGGRHTYSMSHLEYTSYDEIFFLHHSNTDRIFAIWQRLQQLRGKDPNSA---DCAHN-LIH---TPMEPFRRDTNPLDLTREHAKPADSF : 270
OdHG_-_FUa  : E------KKTTARAV-DTRLFQ-AS-KGGKNFIFEGVLSALEQDDYCHFEVQFEVAHNPIHYLVGGRFTHSMSSLEYTSYDELFFLHHSNVERLFTIWQALQKHRGIEGN-A---NCGLN-MFH---KPMEPFGRDTNPISLTKEHAKAVDVF : 266
OdHA_-_FUa  : E------KKTTARAV-DTRLFQ-AS-KGGKNFIFEGVLSALEQDDYCHFEVQFEVAHNPIHYLVGGRFTHSMSSLEYMAYDEFFFLHHSNVERLFTIWQALQKHRGLGGN-A---NCGLN-MFH---KPMEPFGRDTNPISLTKEHAKAVDVF : 266
NpH_-_FUa   : K------KMQTARAV-DERLFSQPE-PGHHTFIFEGILDALEQTDYCQFEVQFEIIHNAIHYLVGGQFPHSMSSLEFTSYDELFFLHHSNVDRLFSIWQALQRHRGLPSGHS---NCAKE-LFT---VPMKPFNEDSNPIQLTHDHALPSQLF : 268
AcH1_-_FUb  : LNS----GAKTSRDV-SDSLNDVPE-WGDHTDLFDQYLYALEQDNFCNFEVQFEVAHNLIHALVGGNTQFGLSSLSYSAFDEIFFLHHSNVDRIWAIWTALQQYRGKPYK-A---HCAQS-YVNQPMKPFA-ESSPLNNNEKTFSHSVPTNVY : 289
AcH2_-_FUb  : LGN----GVVTSRDV-LEGLTQTPT-FGDHTDLFDAYLLALEQDNFCDFEVQFEVAHNMIHALVGGNAPFGLASLSYSAYDEIFFLHHSNVDRIWAIWTALQQHRGKPYK-A---HCAQS-YVHQPMKPFA-ESSPLNNNEKTFSHSVPTNVY : 289
LsH1_-_FUb  : LGD----STKTSRDV-SSSLSDSPK-WGDHTELFDAFLLALEQDNFCDFEVQFEVAHNLIHALVGGKEQYSLATLSYSAFDEIFFLHHSNVDRIWAIWTALQELRGKPYK-A---HCAQS-YTYEPLKPFA-ESSPYNNDQKTFTHAVPRNVY : 289
LsH2_-_FUb  : LDN----NVHTSRDV-QKDLSQTPL-WGDHTELFDAFLLALEQDNFCDFEVQFEIAHNLIHGLVGGNSKYGLSSLSYSAFDEIFYIHHSNVDRIWAIWTALQQHRGKPYK-A---HCAQS-YTYEPLKPFA-ESSPYNNNEKTFSHSTPRNVY : 286
BgH11_-_FUb : LRT----GSRTSR----SWPEQGVN----LDDLKDAVLLALEQDNFCDFEVQFEIAHNLIHALVGGNAPYGMSSLEYSAYDEIFYIHHSFLDRIWSIWMSLQELRGKPYK-A---HCAQS-YIFTPLSPFN-ESTTYNPNPKTYAHSTATNIY : 274
BgH12_-_FUb : L------NVTTGRDA-SLDQ--RIS-SYNQTELFEAFLLALEQEDFCDFEIQFEMAHNLIHSLVGGRHPYSMATLEYTAFDEIFYIHHSGLDRIWAIWTALQELRGKPYK-A---HCAQS-YTNTPMKPFA-ESSPYNNNKTSSHSVPTKIY : 279
HpHaD_-_FUb : LGG----DAKTSRDP-LPELTQTPK-WGDHTELFDVFLLALEQDNFCDFEVQFEIAHNLIHAYVGGNSKYGLSSLSYSAFDEIFYIHHSNIDRIWAIWTALQQHRGKPYK-A---HCAQS-YVYTPLKPFA-EHTPYNNNEKTFAHSTPTDVY : 284
CaHaD_-_FUb : LGG----DAKTSRDP-LPELTQTPK-WGDHTELFDVFLLALEQDNFCDFEVQFEIAHNLIHGYVGGNSQYGLSSLSYSAFDEIFYIHHSNIDRIWAIWTALQQQRGKPYK-A---HCAQS-YVYTPLKPFA-EHTPYNNNEKTFAHSTPTEVY : 284
HpHaN_-_FUb : LGN----DVHTERDI-SPSLAQTPA-WGEHTELFNAFLLALEQDYFCKFAIQFEVAHNLIHGLVGGNTPHGLSTLSYSAFDEIFYIYHSNIDRIWAIWTALQEHRGKPYK-A---HCAQS-YVHTPLKPFA-EKPPYNNDAKTYAHSTATNIY : 289
CaHaN_-_FUb : LGN----DVRTERDI-SESLAQTPA-WGEHTELFNAFLLALEQDNFCKFAVQFEVAHNLIHGLVGGNTSHGLSTLSYSAFDEIFYIYHSNIDRIWAIWTALQQLRGKPYK-A---HCAQS-YVHTPLKPFA-EKPPYNNDAKTFAHSTATNIY : 289
HpHb_-_FUb  : LGH----DVHTSRAV-LPGLSQTPD-WGDHTELYDAFLLALEQDNFCDFEVQFEIAHNLIHGLVGGNSAHGLSSLSYSAFDEIFYIYHSSIDRIWAIWTALQQHRHKPYK-A---HCAQS-HVHTPLKPFA-ESSPYNNDEKTYSHSTPTNVY : 289
CaHb_-_FUb  : LGH----DVHTSRDV-LPGLSQVPD-WGDHTELYDAFLLALEQDNFCDFEVQFEIAHNLIHGLVGGNSAYGLSSLSYSAFDEIFYVYHSSIDRIWAIWTALQQRRNKPYR-A---HCAQS-HVHTPLKPFA-ESSPYNNDEKTYSHSIPTNVY : 289
HtH1_-_FUb  : EEG----HHHTSRMI-DSKLFAPVA-FGEHSHLFDGILYAFEQEDFCDFEIQFEIVHNSIHAWIGGSEDYSMATLHYTAFDPIFYLHHSNVDRLWAIWQALQIRRHKPYQ-A---HCAQS-VEQLPMKPFA-EPSPLNNNEKTHSHSVPTDIY : 281
KLH1_-_FUb  : EEN----APHTKRQI-DQRLFKPAT-FGHHTDLFNQILYAFEQEDYCDFEVQFEITHNTIHAWTGGSEHFSMSSLHYTAFDEIEYFHHSNVDRLWAVWQALQMRRHKPYR-A---HCAIS-LEHMHLKPFA-ESSPLNNNEKTHANAMPNKIY : 281
HtH2_-_FUb  : E------NGRTERHP-DSRLFEQPL-FGKHTRLFDSIVYAFEQEDFCDFEVQFEMTHNNIHAWIGGGKYSMSSLHYTAFDEISYLHHSNTDRLWAIWQALQIRRNKPYK-A---HCAWS-EERQPLKPFA-ESSPLNNNEKTYENSVPTNVY : 280
KLH2_-_FUb  : E------NNVTHRDT-DNRLFEKPS-FGHHTHLFDAMVYAFEQEDFCDFEVQFEIVHNNIHAWVGGSEKYSMSSLHYTAFDEIFYLHHSNVDRLWAIWQALQIRRGKSYK-A---HCASS-QEREPLKPFA-ESSPLNNNEKTYHNSVPTNVY : 280
OdHG_-_FUb  : LHN----DVFTARNV-DSRLLFEKPT-KGHHGYLHDGMLLAFEQEDFCDFEVQFEVTHNAIHAWVGGNEPYSMSSLHYISFDPLFWLHHSQVDRLWAVWQALQIHRGKPYK-P---YCALS-EVHRPLKPFA-EEPPLNNNKHTHSHSVPTHVY : 282
OdHA_-_FUb  : LHN----DVFTARNV-DSRLLFEKPT-KGHHGYLHDGMLLAFEQEDFCDFEVQLEVTHNAIHAWVGGNEPYSMSSLHYTSFDPLFWLHHSQVDRLWAVWQALQIQRGKPYK-P---YCALS-EVHRPLKPFA-EEPPLNNNKHTHSHSVPTHVY : 282
NpH_-_FUb   : LHD----HITTQRDIKQETLFEQPA-FGHHTYLFDGLMYALEQDDFCDFEIQFEIVHNGIHAWTGGQQTYSMSSLHYTSFDPIFFLHHSNVDRLWAIWQALQIYRGKHYL-A---HCANS-EVHSPMRPFS-EPSRNPNPRTFTHATPTDIY : 282
AcH1_-_FUc  : A------NADTVRSV-RDDLFQQPP-FGEYTDIAKQVLLALEQDDFCTFEVQYEIAHNFIHALVGGSEQFSMASLQYTAFDEIFFLHHSNTDRIWAIWQALQKYRGKPYNTA---NCAIG-QLRNPLSPFS-LTSDINPDPVTREHSVPFQVF : 280
AcH2_-_FUc  : V------DQYTVRSV-RDDLYQQPN-FGDYTDIAKQVLLALEQDDFCTFEVQYEIAHNFIHALVGGSEQFSMASLQYTAFDEIFFLHHSNTDRIWAIWQALQKYRGKPYNTA---NCAIG-QLRNPLSPFS-LTSDINPDPVTREHSIPFQAF : 280
LsH1_-_FUc  : A------DQDTVRSP-RDDLFEQPK-FGQNTAIAKQVLLALEQDNFCDFEVQFEIAHNFIHALVGGNEQFSMASLLYTAFDELFYLHHANTDRLWAIWQALQKYRNKPYNTA---NCAIG-QLRKPLSPFS-LTSSVNPDANTREHSLPFQVF : 280
LsH2_-_FUc  : V------DHDTVRSV-RSDLFQQPG-FGEYTDIAEQILLALEQDDFCDFEVQFEIAHNFIHALVGDKEPYSMASLMYTAFDEIFFLHHSNTDRIWAIWQALQNYRGKPYNTA---NCAIG-PLRQPLAPFS-LTSDVNPDAVTREHSVPFQVF : 280
BgH12_-_FUc : X------XXXXXXXXXXXXXX-XXX-XXXXXXXXXXXXLAMEQEDFCDFEVQFEIAHNCINVHIGGSELYSMSSLKYAAFEPLFLVFHHANADRIWAIWQALQKLRNKPYLTA---NCAQG-LMQTPLSPYN-LTDGINRYSNTQGLSKPSQVF : 281
HpHaD_-_FUc : V------NQNTVRSV-RSDLYQQPA-FGEYTDIAKQVLYALEQDNYCDFEVQYEIAHNFIHALVGGSEVYSMASLLYTAFDELFYLHHSNTDRIWAIWQALQAYRGKPENAA---NCAIG-KLRIPLPPFS-LTSDVNPDPVTREHSSPLSVF : 280
CaHaD_-_FUc : V------NQNTVRSV-RDDLYQQPA-FGEYTDIAKQVLYALEQDNYCDFEVQYEIAHNFIHALVGGSEVYSMASLLYTAFDELFYLHHSNTDRIWAIWQALQTYRGKAENAA---NCAIG-KLRKPLPPFS-LTSDVNPDPVTREYSSPLRVF : 280
HpHaN_-_FUc : V------NQDTERSP-RPELFQKPK-YGEYTSIAKQVLLAFEQEDFCDFEVQFEIAHNFIHTLVGGSKPYSMASLAYTAYDEIFYLHHSNTDRLWAIWQALQKNRGKPHNSA---NCATG-LLRKPLAPFS-LDSDVNPDPVTKAHSQPYQVF : 280
CaHaN_-_FUc : A------NQDTERSP-KPELFLKPK-YGDYTAIAKQVLLAFEQEDFCDFEVQFEIAHNFIHTLVGGSKPYSMASLAYTAYDELFFLHHSNTDRLWAIWQALQKNRGKSHNSA---NCATG-LLRKPLAPFS-LDSDVNPDPVTKAHSQPHQVF : 280
HpHb_-_FUc  : A------SADTVRSV-RPDLFVDPG-FGHYTDIAKQVLLAFEQDNFCDFEVQFEIAHNFIHALVGGSEVYSMASLAYTAYDPLFYLHHSNTDRLWAVWQALQQYRGKPFNSA---NCAVD-KIRKPLAPFS-LSSDVNPDPVTREHSLPFQVF : 280
CaHb_-_FUc  : A------SADTVRSV-RPDLFKNPG-FGHYTDIAKQVLLAFEQDNFCDFEVQFEIAHNFIHALVGGSELYSLASLAFTAYDELFYLHHSNTDRLWAVWQALQQHRGKPFNSA---NCAVD-KIRKPLAPFS-LSSDVNPDPVTREHSLPFQVF : 280
HtH1_-_FUc  : V------NQDTTRSV-REDLYQQPE-FGHFTDIAQQVLLALEQDDFCSFEVQYEISHNFIHALVGGTDAYGMASLRYTAYDPIFFLHHSNTDRLWAIWQSLQKYRGKPYNTA---NCAIE-SMRRPLQPFG-LSSAINPDRITREHAIPFDVF : 280
KLH1_-_FUc  : V------DKTTTRSV-QNKLFEQPE-FGHYTSIAKQVLLALEQDNFCDFEIQYEIAHNYIHALVGGAQPYGMASLRYTAFDPIFFLHHSNTDRLWAIWQALQKYRGKPYNVA---NCAVT-SMRKPLQPFG-LSANINTDHVTKEHSVPFNVF : 280
HtH2_-_FUc  : V------GVDTTRSV-RQELYEAPG-FGHYTGVAKQVLLALEQDNFCDFEVQFEIAHNFIHALVGGSEPYGMASLRYTTYDPIFYLHHSNTDRLWAIWQALQKYRGKPYNSA---NCAIA-SMRKPLQPFG-LTDEINPDDETRQHAVPFSVF : 280
KLH2_-_FUc  : V------GHDTTRDV-RDDLYQSPG-FGHYTDIAKQVLLAFEQDDFCDFEVQFEIAHNFIHALVGGNEPYSMSSLRYTTYDEIFFLHRSNTDRLWAIWQALQKYRGKPYNTA---NCAIA-SMRKPLQPFG-LDSVINPDDETREHSVPFRVF : 280
OdHG_-_FUc  : I------GEHTTRSP-NPTLFLKPP-FGHFTPLGDEVMYALEQEDFCSFEVQFEIAHNHIHALVGGTEPYSMSSLEYTTFDPIFILHHSNVDRIWAIWQALQKFRGHRYNSA---NCAIE-TLRKPMSPFS-LTSDIINIDPMTREHSVPFDVF : 280
OdHA_-_FUc  : I------GEHTTRFP-NPTLFLKPP-FGHFTPLGNEVMYALEQEDFCSFEVQFEIAHNHIHALVGGTEPYSMSSLEYIAFDPIFILHHSNVDRIWAIWQALQKFRGHRYNSA---NCAIE-TLRKPMSPFS-LTSDINVDPMTREHSVPFDVF : 280
NpH_-_FUc   : V------NADTMRNI-NEKLFEQPE-FGHFTLLANQVILALEQENFCDFSVQFEISHNEIHALVGGTEPYSMASLTYTAFDELFMLHHSNVDRIWAIWQSLQQYRGHPHKSA---NCALQ-GLHQPLSPFS-LTSEVNPDPETREHSKPMDVF : 280
AcH1_-_FUd  : E------DAVTTRDP-QPQLFN-------NDYFYQQTLFALEQTNFCDFEIQFEIIHNALHSWLGGRAEYSFSSLDYTAFDEVFFLHHANTDRIWAIWQELQRYRGLPYNEA---DCAIN-LMRKPLQPFN-N-DDEVHGDATNRYSRPADTF : 274
AcH2_-_FUd  : E------DAVTTRDP-QAQLFN-------NDHFYQQTLFALEQTNFCDFEIQFEIIHNALHSWLGGRAEYSFSSLDYTAFDEVFFLHHANTDRIWAIWQELQRYRGLPYNEA---DCAIN-LMRKPLQPFN-N-DELNHGDLTSRYSRPADTF : 274
LsH1_-_FUd  : E------GAVTTRDP-QPELFN-------NDDLYEQVLYALEQDHFCDFEIQFEIIHNALHSLLGGRAQYSLSSLDYSAFDEVFFLHHANTDRIWAIWQELQRYRGLKYNEA---DCAIN-QMRTPLRPFN-D-DQLNHGDLTHRYSRPADTF : 275
LsH2_-_FUd  : E------NAVTTRDP-QPELFN-------SEELYEQVLLALEQEHFCDFEIQFEIVHNALHTLLGGRSQYSLSSLDYSAFDEVFFLHHANTDRIWAIWQELQRYRGLPYNEA---DCAIN-LMRKPLQPFN-N-DSLNHGDLTHRYSQPAETF : 274
BgH12_-_FUd : E------NAFTTRDP-QGELFN-------NKDLYEQALYVLEQEHFCDFEIQFEVVFQSAIHFLVGGRSLFSLSSLDYSAFDPVFIIHHRINTDRIWAIWQELQRYRRLPYSHA---DCAIN-KMKKHLEPFD-R-PELNKNKDTAKFSRPADTF : 274
HpHaD_-_FUd : E------DAVTTRDP-QPELFN-------NDYFYEQALYALEQDNFCDFEIQFEVLHNALHSWLGGHAKYSFSSLDYTAFDEVFFLHHANTDRLWAIWQELQRYRGLPYNEA---DCAIN-LMRKPLQPFQ-D-KKLNPRNITNIYSRPADTF : 274
```

```
CaHaD_-_FUd : E------DAVTTRDP-QPELFN-------NDYFYEQALYALEQYSFCDFEIQFEVLHNALHSWLGGHAKYSVSSLDYTAYDPVFFLHHANTDRLWAIWQEIQRYRGLPYNEA---DCAIN-LMRKPLQPFQ-D-KTLNPRNITNIYSRPADTF : 274
HpHaN_-_FUd : E------NAVTTRDP-QPELFN-------NDYFLEQALFALEQDHYCDFEIQFEILHNALHSWLGGHALYSMASLDYSAFDPVFFLHHANTDRIWAIWQELQRYRGLQYNEA---DCAIN-LMKKPLQPFN---RTTNTDEVTRKYSRPVDTF : 273
CaHaN_-_FUd : E------NAVTTRDP-QPELFN-------NDYFLEQALFALEQDHYCDFEIQFEILHNALHSWLGGHAFYSMASLDYAAFDPVFFLHHANTDRIWAVWQELQRYRGLQYNEA---DCAIN-LMRKPLEPFN---RVTNTDEVTRKNSRPVDTF : 273
HpHb_-_FUd  : E------NATTTRNP-QTALFN-------NDYFYEQVLFAFEQTHFCDFEIQLEVTHNAIHSWLGGHARYSMASLDYAAFDPVFFLHHTNTDRLWAIWQELQRYRGLPYNEA---DCAIN-QMRKPLKPFG---DSDNKDNVTKKYSRPVDTF : 273
CaHb_-_FUd  : E------NASTTRNP-QAALFN-------NDYFYEQVLFAFEQTHFCDFEIQLEVTHNAIHSWLGGHARYSMASLDYAAYDPVFFLHHTNTDRLWAIWQELQRYRGLPYNEA---DCAIN-QLRKPLKPFG---DSDNKDNVTRKYSRPIDTF : 273
HtH1_-_FUd  : E------NAVTTRDP-QAGLFN-------SDYMYENVLLALEQENYCDFEIQFELVHNALHSMLGGKGQYSMSSLDYSAFDPVFFLHHANTDRLWAIWQELQRFRELPYEEA---NCAIN-LMHQPLKPFS-D-PHENHDNVTLKYSKPQDGF : 274
KLH1_-_FUd  : E------NEITTRDP-KDSLFH-------SDYFYEQVLYALEQDNFCDFEIQLEILHNALHSLLGGKGKYSMSNLDYAAFDPVFFLHHATTDRIWAIWQDLQRFRKRPYREA---NCAIQ-LMHTPLQPFD---KSDNNDEATKTHATPHDGF : 273
HtH2_-_FUd  : E------NAVTTRDP-QPELYV-------NRYYYQNVMLVFEQDNYCDFEIQFEMVHNVLHAWLGGRATYSISSLDYSAFDPVFFLHHANTDRLWAIWQELQRYRKKPYNEA---DCAIN-LMRKPLHPFD-N-SDLNHDPVTFKYSKPTDGF : 271
KLH2_-_FUd  : E------NAVTSRDP-QPELWD-------NKDFYENVMLALEQDNFCDFEIQLELIHNALHSRLGGRAKYSLSSLDYTAFDPVFFLHHANVDRIWAIWQDLQRYRKKPYNEA---DCAVN-EMRKPLQPFN-N-PELNSDSMTLKHNLPQDSF : 274
OdHG_-_FUd  : L------NSETNRDP-QEELFG-------NKYLYEHTLFVLEQTDFCDFEVHFEVLHNTIHSWLGGRDPHSMSSLDFAAYDPIFFLHHSNIDRIWAIWQELQRYRKLPYNEA---NCALP-LLNVPMRPFS-N-TTANHDRMTLTHSAPNDVF : 277
OdHA_-_FUd  : L------NSETNRDP-QEKLFG-------NKYLYEHTLFVLEQTDFCDFEVHFEVLHNTIHSWLGGRDPHSMSSLDFAAYDPIFFLHHSNIDRIWAIWQELQRSRKVPYNEA---NWALP-LLNVPMRPFS-N-TTANHDRMTLTHSAPNDVF : 277
NpH_-_FUd   : I------NSHTSRDF-QPELFN-------SDYLYENTLLALEQNDFCNFEVQLEVLHNAIHAWIGGRDTYSMSSLDYAAYDPIFFIHHSTLDRIWAIWQELQRYRGLPYNEA---NCALP-LMNQHLRPFN-N-VSVNHDRNTLTLNKPNDAF : 276
AcH1_-_FUe  : E------NSVTERDVQGDYLFKQGP-HGFDTWLFNQALLALEQEDYCDFEIQFEITHNAVHSWLGGTKVHSMGHLHYASYDPAFYVHHSNTDRLWALWQALQKYRGLNPNEA---NCALE-QMRVPLKPFS-FGAPYNLNPKTKEFSKPEDTF : 284
AcH2_-_FUe  : E------NTTTERDVQGDYLFKQGP-HGFDTWLFNQALLALEQEDYCDFEIQFEITHNAVHSWLGGTKVHSMGHLHYASYDPAFYVHHSNTDRLWALWQALQKYRGHNPNEA---NCALE-QMREPLKPFS-FGSPYNLNEMTKEYSKPEDTF : 284
LsH1_-_FUe  : E------NSETEREVVSDRLFKRGP-HGWDTWLFNQALLALEQEDYCNFEIQFEITHNAIHSWVGGSKEHSLGHLHYASYDPAFFVHHSNTDRLWAVWQALQKYRGYDPNEA---NCALE-QMKDPLKPFS-FGPPYNLNPLTKEHSRPEDTF : 284
LsH2_-_FUe  : E------HAVTERDVKEEFLFKVGK-HGWDTWLFNQALYALEQEDYCDFEVQFEITHNAIHSWLGGSKNYSLGHLHYASYDPAFFIHHSNTDRLWAVWQALQKYRGHDPNEA---NCALE-LMREPLKPFS-FGPPYNLNQMTQDNSRPEDTF : 284
BgHl2_-_FUe : E------AVYTDRDILVDKMFKMGP-LGWDTWMFEQALFALEQDNYCDFQVQLEVISQVLSVWIGGSETHSMSHLHFASYDPLFFVHHANVDRLWAVWQALQKHRGHDPDKS---NCALE-MMKKPLKPFS-FGSPYNLNEVTREKSLPTAVF : 284
HpHaD_-_FUe : E------HSHTERDIQSAELFKLGP-HGWDTWLFEQALLALEQEDYCDFEIQFEITHNAIHSWVGGSKEHSLAHLHYASYDPAFYIHHSNTDRLWAIWQALQKHRGYSPNEA---NCALE-HLKDSLKPFS-FGPPYNLNKLTTKYSHPEDTF : 284
CaHaD_-_FUe : E------NSHTERDIQSAELFKLGP-HGWDTWLFEQALLALEQEDYCDFEIQFEITHNAIHSWVGGSKEHSLAHLHYASYDPAFYIHHSNTDRLWAIWQALQKHRGYNPNEA---NCALE-HIKDSLKPFS-FGPPYNLNKLTTKYSHPEDTF : 284
HpHaN_-_FUe : E------HVDTERDVQEDKLFKRGP-HGWDTWLYTQVLFALEQEDFCDFEVQLEIAHNAIHSWIGGSKEHSLAHLHYASYDPAFFIHHSNTDRLFAVWQKLQTYRGHDANSA---NCALE-QLREPLKPFS-FGPPYNLNKVTQDNSRPEDTF : 284
CaHaN_-_FUe : E------HVDTERDVQEDKLFKSGPH-GWDTWLYTQVLFALEQEDFCDFEVQLEIAHNAIHSWIGGSKEHSLAHLHYASYDPAFFIHHSNTDRLFAVWQKLQQYRGHDPNSA---NCALE-QLREPLKPFS-FGPPYNLNKVTQEYSRPQDTF : 284
HpHb_-_FUe  : E------HEDVHRDI-QSNLLFKRGPHGWDTWLYNQALYALEQEDYCDFAVQLEITHNSIHAWIGGSQKHSMAHLHYASYDPIFFIHHSNTDRLWAVWQALQEYRGHSANEV---NCALE-KLREPLKPFS-FGPPYNLNPVTLENSHPGDTF : 284
CaHb_-_FUe  : E------HEDVHRDI-QSDLLFKRGPHGWDTWLYNQALYALEQEDYCDFAVQLEITHNSIHAWIGGSQKHSMAHLHYASYDPIFFIHHSNTDRLWAVWQALQKHRGHNAYEV---YCSLE-KLREPLKPFS-FGPPYNLNPVTRQNAHSGDTF : 284
HtH1_-_FUe  : EGP----GVHTERHINTERLFHSGDHDGYHNWFFETVLFALEQEDYCDFEIQFEIAHNGIHTWIGGSAVHGMGHLHYASYDPIFYIHHSQTDRIWAIWQELQKYRGLSGSEA---NCAIE-HMRTPLKPFS-FGPPYNLNSHTQEYSKPEDTF : 287
KLH1_-_FUe  : EGE----NVHTKRDINRDRLFQGST-KTHHNWFIEQALLALEQTNYCDFEVQFEIMHNGVHTWVGGKEPYGIGHLHYASYDPLFYIHHSQTDRIWAIWQSLQRFRGLSGSEA---NCAVN-LMKTPLKPFS-FGAPYNLNDHTHDFSKPEDTF : 286
HtH2_-_FUe  : EGE----NITTEREVIADKLFVKGG-HVFDNWFFKQAILALEQENYCDFEIQFEILHNGVHTWVGGSRTYSIGHLHYAFYDPLFYLHHSQTDRIWAIWQELQEQRGLSGDEA---HCALE-QMREPLKPFS-FGAPYNWNQLTQDFSRPEDTF : 286
KLH2_-_FUe  : EGE----GVTTERDVDAEHLFAKGN-LVYNNWFCNQALYALEQENYCDFEIQFEILHNGIHSWVGGSKTHSIGHLHYASYDPLFYIHHSQTDRIWAIWQALQEHRGLSGKEA---HCALE-QMKDPLKPFS-FGSPYNLNKRTQEFSKPEDTF : 286
OdHG_-_FUe  : ISE----DTETKREV-SEYLFEHPV-LGKQTWLFDNIALALEQTDYCDFEIQLEIVHNAIHSWIGGKEEHSLNHLHYAAYDPIFYLHHSNVDRLWVIWQELQKLRGLNAYES---HCALE-LMKVPLKPFS-FGAPYNLNDLTTKLSKPEDMF : 286
OdHA_-_FUe  : ISE----DTETKREV-SEYLFEHPV-LGKQTWLFDNIALALEQTDYCDFEIQLEIVHNAIHSWIGGKEEHSLNHLHYAAYDPIFYLHHSNVDRLWVIWQELQKLRGLNAYES---HCALE-LMKVPLKPFS-FGAPYNLNDLTTKLSKPEDMF : 286
NpH_-_FUe   : ISP----EVMTQRDV-QDKLFKQPK-LGRNTWLHDNVILALEQTDYCNFEVQFEIVHNAIHAWVGGSQEHSLSHLHFAAYDPAFYLHHSNVDRIWVIWQELQKIRGLSAYEA---NCALE-LMNEPLKPFA-FGSPYNLNEMTRKYSLPEDTF : 287
AcH1_-_FUf  : E------DVYTVRDI-RPEIRNKNQ-AGDHSAIFDLVLSALEQTDYCDFEVQFEVMHNAIHFLVGGLQTYSLSSLEYSAYDPLFFIHHSFTDKIWAVWQELQTRRHLPANKA---DCALN-YMSQPMRPFF-F-EGFNLNKFTMDHAVPNTVF : 279
AcH2_-_FUf  : E------KAYTVRDV-RPEIHLNDR-EGRHSALFDLVLLALEQTDYCDFEVQFEVMHNAIHFLVGGLQTYSMSTLEYSAYDPMFLIHHSFTDKIWIVWQELQKRRHLPATSA---ACAVN-YMNQPMKPFY-F-EGFNLNGFTKSHALPSSVF : 279
LsH1_-_FUf  : E------NTYTVRDI-RPELFQTSK-DGSHSDLFELVLSALEQTDYCDFEVQFEVMHNAIHYLVGGLQTYSLSSLEYSAYDPIFFIHHSFVDKIWVVWQELQQRRHLPSNTA---NCALN-YLAEPMKPFN---LDLNINAFTKKHAVPRDVF : 278
LsH2_-_FUf  : E------KVYTVRET-KSELYDKGR-GGKNSALIYYVISALEQTDYCDFEVQFEVIHNSIHYLVGGEQTYSLSSLEYSAYDPIFFIHHSFTDKIWAVWQELQKRRRLPYLKA---NCALN-YMAEPMKPFS-I-DGLNLNKFTKEHAVPDKLF : 279
BgHl2_-_FUf : E------DTYTVRDV-RHELFXXXX-XXXXXXXXXXXXXXXXXXXXXXXXAQFQVVHSTILNSLGGDQLYSLSTLEYSAYDPIFIIHNSFMDKIWLIWQELQKERHLPSLGT---ECALN-YMNQPLQPFN---SELNMNTFTKEHSLPKDLF : 278
HpHaD_-_FUf : E------DTYTVRDI-QETLHDRHV-DGKHSFLLYGVLEVLEQTDYCDFEVHFEVVHNAIHYLIGGHQTYSLSSLEYSAYDPIFFIHHSFTDKLWAVWQELQKRRHFPYNRA---DCALN-YVNEPLKPFS-F-EGFNLNKFTREHAVPNTLF : 279
CaHaD_-_FUf : E------DTYTVRDI-QSPLHK-KH-DGKHSFLLHGVLDVLEQTDYCDFEVQFEVVHNAIHYLIGGHQTYSLSSLEYSAYDPIFFIHHSFTDKIWAVWQELQKRRNLPYNRA---DCALN-YVNEPLKPFS-F-EGLNLNKFTREHAVPNTLF : 277
HpHaN_-_FUf : E------KAQTVRDV-QPELFETTK-DGKHSTLFPLLLEALEQTSYCDFAVQFEVLHNAIHYLVGGHQKYSLSSLEYSAYDPIFFIHHSFSDKLWVVWQELQQRRHLPYHTA---DCAVS-AMSEPMKPFS-F-ETFNTNKFTRDHAGPNSLF : 279
CaHaN_-_FUf : E------KAQTVRDV-QPELFETTE-DGKHSTLFPLLLEALEQTSYCDFAVQFEVLHNAIHYLVGGHQKYSLSSLEYSAYDPIFFIHHSFSDKLWVVWQELQQRRHLPYHTA---DCAVN-AMSEPMKPFS-F-ETFNTNKFTREHSVPNTVF : 279
HpHb_-_FUf  : E------GAYTVRDV-YQHFFRAYG-DGKHSFLFYIALEALEQTDYCDFEVQFEILHNAIHYLVGGEQKYSLSSLEYSAYDPLFFIHHSFVDKIWVVWQKLQQRRHLPYNRA---DCAVA-AVNQPMKPFT-F-ESINPNKFTREHALPSSVF : 279
CaHb_-_FUf  : E------DAYTVRDV-YPGFYKSFG-DGKHSFLFIIALQALEETDYCNFEVQFEILHNAIHYIVGGEQKYSLSSLEYSAYDPLFFIHHSFTDKIWVVWQELQQRRHLPYNRA---DCAVV-AMNQPMKPFT-F-ESLNLNKFTREHALPSSVF : 279
HtH1_-_FUf  : K------DAFTVRNV-QESLFKMSS-FGKHSLLFDQALLALEQTDYCDFEVQFEVMHNTIHYLVGGRQTYAFSSLEYSSYDPIFFIHHSFVDKIWAVWQELQSRRHLQFRTA---DCAVG-LMGQAMRPFN---KDFNHNSFTKKHAVPNTVF : 278
KLH1_-_FUf  : H------DTYTVRDV-QEGLFHLTS-TGEHSALLNQALLALEQHDYCDFAVQFEVMHNTIHYLVGGPQVYSLSSLHYASYDPIFFIHHSFVDKVWAVWQALQEKRGLPSDRA---DCAVS-LMTQNMRPFH-Y--EINHNQFTKKHAVPNDVF : 278
HtH2_-_FUf  : A------NTYTVRDP-QEMLFQLCE-HG-ESILYEQTLLALEQTDYCDFEVQFEVLHNVIHYLVGGRQTYALSSLHYASYDPFFFIHHSFVDKMWVVWQALQKRRKLPYKRA---DCAVN-LMTKPMRPFD---SDMNQNPFTKMHAVPNTLY : 277
KLH2_-_FUf  : E------DAYTVRDP-QDILYHLQD-ETGTSVLLDQTLLALEQTDFCDFEVQFEVVHNAIHYLVGGRQVYALSSQHYASYDPAFFIHHSFVDKIWAVWQALQKKRKRPYHKA---DCALN-MMTKPMRPFA---HDFNHNGFTKMHAVPNTLF : 278
OdHG_-_FUf  : E------DTYTVRDV-KPELFE-IG-GGEGSTLYQQVLLMLEQEDYCDFEVQFEVVHNSIHYLVGGHHKYAMSSLVYSSFDPIFYVHHSMVDRLWAIWQALQEHRHLPFDKA---YCALE-QLSFPMKPFV---WDSNPNLHTRAASTPQHLF : 276
OdHA_-_FUf  : E------DTYTVRDV-KPELFE-IG-GGEGSTLYRQVLLMLEQEDYCDFEVQFEVVHNSIHYLVGGHQKYAMSSLVYSSFDPIFYVHHSMVDRLWAIWQALQEHRHLPFDKA---YCALE-QLSFPMKPFV---WESNPNLHTRAASTPQHLF : 276
NpH_-_FUf   : A------ETYTVRNT-QPELFEPSK-DGKHSLLYDEILLALEQRDYCDFEVQFEVVHNAIHYLVGGHQRYSMSSLEYASYDPLFFIHHSNVDRLWAIWQALQQYRRIPYNKA---YCALE-QMTHAMKPFD---RETNPNKNTHDHAIPNSVF : 277
AcH1_-_FUg  : N------GEVTSRAP-REQLFNDPE-FGSESFFYRQALLAFEQTDYCDFEVQYEITHNAIHSWTGGRSPYGMSTLEFTAYDPLFLLHHSNADRQFAIWQALQKFRGLPYNNA---NCAIQ-KLREPMKPFS---NLDNINPTTRANARAIDAF : 270
AcH2_-_FUg  : N------GEVTSRAP-REQLFNDPE-FGSESFFYRQALLAFEQTDYCDFEVQYEITHNAIHSWTGGRSPYGMSTLEFTAYDPLFLLHHSNVDRQFAIWQALQKFRGLPYNNA---NCAIQ-KLREPMKPFS---NSDNINPTTRANARATDAF : 269
LsH1_-_FUg  : N------GEITTRAP-RDQLFNDPE-FGSESFFYRQALFTFEQTDYCNFEVQFEITHNAIHSWTGGSSQYGMSSLEYTAYDPLFLLHHSNVDRQFAIWQALQKFRGLPYNSA---NCAIQ-HLRQPMRPFS---DENNLNAITRANSRAIDAF : 269
LsH2_-_FUg  : N------GEVTTRAP-RDKLFNDPE-FGAESFFYRQVLLAFEQTDYCDFEVQFEITHNAIHSWTGGSSPYGMSTLEYTAYDPLFLLHHSNVDRQFAIWQALQKFRGLPYNSA---NCAIQ-HLRQPMRPFS---DESNINAITRANSRAIDAF : 269
HpHaD_-_FUg : N------GEITTRAP-RDKLFNDPE-FGKESFFYRQVLLAFEQTDYCDFEVQYEISHNAIHSWTGGQSPYGMSTLEYTAYDPLFLLHHSNVDRQFAIWQALQKFRGLPYNSA---NCAIQ-LLHQPMRPFS---DADNVNPVTRTNSRARDVF : 269
CaHaD_-_FUg : N------GQNTTRAP-RGRLFNHPE-FGRESFFYRQILLAFEQTDYCDFEVQYEISHNAIHSWTGGKSPYGMSTLEYTAYDPLFLLHHSNVDRQFAIWQALQKFRGLPYNSA---NCAIQ-LLHQPMRPFS---DSDNINPVTRTNSRARDVF : 269
HpHaN_-_FUg : D------GHITTRAP-RSLLFNDPE-FGDESFFYRQVLLAFEQTDYCDFEVQFEITHNAIHSWVGGQSPYGLSTLEYTAYDPLFLLHHSNVDRQFSIWQALQKYRGLPYNTA---NCAIQ-DLRRPLRPFS---DPANVNPTTRANSRAIDAF : 269
CaHaN_-_FUg : D------GHVTTRAP-RSLLFNDPE-FGDESFFYRQVLLAFEQTDYCDFEVQFEITHNAIHSWVGGQSPYGLSTLEYTAYDPLFLLHHSNVDRQFAIWQALQKHRGLPYNTA---NCAIQ-ELRRPLRPFS---DKENVNPTTRANSRAIDAF : 269
HpHb_-_FUg  : -------DHNTTRSP-RPQLFSDPA-SGNESFFYRQVLLAFEQTDYCDFEVQFEFAHNAIHSWTGGRSPYGMSTLEYTAYDPLFLLHHSNVDRQFAIWQALQKFRGLPYNSA---NCAIQ-LLHQPMRPFS---DSDNVNPTTRAHSTASEAF : 269
CaHb_-_FUg  : -------DHNTTRSP-RPQLFSDPE-HGEESFFYRQVLLAFEQTDYCDFEVQFEFAHNAIHSWIGGQSPYSMSTLEYTAYDPLFLLHHSNVDRQFAIWQALQKFRGLPYNSA---NCAIQ-LLHQPMRPFS---DADNVNPTTRSHSTASEAF : 269
HtH1_-_FUg  : L------NVSTTRSP-RDMLFNDPE-HGSESFFYRQVLLALEQTDFCKFEVQFEITHNAIHSWTGGHSPYGMSTLDFTAYDPLFWLHHSNTDRIWAVWQALQEYRGLPYNHA---NCEIQ-AMKTPLRPFS---DDINHNPVTKANAKPLDVF : 271
KLH1_-_FUg  : L------GVDTTRSP-RDKLFNDPE-RGSESFFYRQVLLALEQTDFCQFEVQFEITHNAIHSWTGGLTPYGMSTLEYTTYDPLFWLHHANTDRIWAIWQALQEYRGLPYDHA---NCEIQ-AMKRPLRPFS---DPINHNAFTHSNAKPTDVF : 271
HtH2_-_FUg  : L------GVTTSRSP-RDMLFNDPE-QGSESFFYRQVLLALEQTDYCQFEVQFELTHNAIHSWTGGRSPYGMSTLEFTAYDPLFWLHHSNTDRIWAVWQALQKYRGLPYNEA---HCEIQ-VLKQPLRPFN---DDINHNPITKTNARPIDSF : 271
KLH2_-_FUg  : R------NVDTSRSP-RDMLFNDPE-HGSESFFYRQVLLALEQTDFCQFEVQFEITHNAIHSWTGGHTPYGMSSLEYTAYDPLFYLHHSNTDRIWAIWQALQKYRGFQYNAA---HCDIQ-VLKQPLKPFS---ESRNPNPVTRANSRAVDSF : 270
OdHG_-_FUg  : A------NTDTTRSP-RAQLFDDPE-KGDKSFFYRQIALALEQTDFCDFEIQFEIGHNAIHSWVGGSSPYGMSTLHYTSYDPLFYLHHSNTDRIWSVWQALQKYRGLPYNTA---NCEIN-KLVKPLKPFN---LDTNPNAVTKAHSTGATSF : 271
OdHA_-_FUg  : A------NTDTTRSP-RAQLFDDPE-KGDKSFFYRQIALALEQTDFCDFEIQFEIGHNAIHSWVGGSSPYGMSTLHYTSYDPLFYLHHSNTDRIWSVWQALQKYRGLPYNTA---NCEIN-KLVKPLKPFN---LDTNPNAVTKAHSTGATSF : 271
NpH_-_FUg   : I------NETTSRNP-RPQLFTDQR-YGETSFFYRQILLALEQTDFCDFEIQFEVGHNAIHSWVGGSTQYGMSTLHFTSYDPLFYIHHSNTDRIWAIWQALQRYRGLPYNSA---NCEIH-LLKKPLEPFS---FESNPNHETHNHSTGNAVF : 267
```

```
AcH1_-_FUh  : V------NQDTTRDI-QDSLFNPRT-INGYNYLYYLALSTLEEDSFCDFEIQYEILHNEIHGLIGGHGTYSMSTLDYSAFDPLFMIHHSSIDRIWAIWQQLQKLRRKPFNSA---RCGGA-IMEEPLQPFS-Y-SQINTNDFTRMNSQPSKVF : 275
LsH1_-_FUh  : A------NEKTTRQV-QSELYSERK-VHGFPYLFYLALTTLEEDNYCDFEVQFEVLHNEIHADIGGSGTYSMATLDYSAFDPFFMIHHSSIDRIWVIWQELQKLRHKPFNGA---SCAGH-IMERPLQPFS-Y-PEVNKNEFTRLNSVPNVVF : 275
LsH2_-_FUh  : V------GKKTTRDV-KDELFNPPT-INGFNQLYYLALSTLEEDNYCDFEVQYEILHNQIHALIGGTGTYSMATLDYSAFDPFFMIHHSSIDRIWVIWQELQKIRKKPFNAA---HCGGH-LMETPLQPFS-Y-PEVNPNDLTRLNAVPNLVF : 275
BgHl1_-_FUh : E------KEWTDREV-NLKQLNLLD-PEGTKMLFHSALSILEEDQFCDFAVQFELLHYRLHALMGGTKKYSLATLDFSAFDPLFMILQSSFDRLWTMWQQLQYLRQKTVS-G---QCVYK-HVDSSMEPFR-N-PDINVNKMTRENSLPGLVS : 266
HpHaD_-_FUh : N------NQRTTRDI-QSELYNPHQ-INGYNYLYYLALSTLEEDNFCDFEVQYEVLHNEIHALIGGNGTYSMATLDFSAFDPFFMIHHSSLDRIWIIWQELQKLRHKPFNYA---HCGGH-VLDDPLHPFS-F-GEINKNDLTRLNSQPSSVF : 275
CaHaD_-_FUh : N------NQRTTRDI-QGELYNPHT-INGFNYIYYLALSTLEEDNFCDFEVQYEVLHNEIHALIGGNGTYSMATLDFSAFDPFFMIHHSSLDRIWIIWQELQKLRHKPFNYA---QCGGH-VLDDPLHPFS-F-GDINKNDLTRLNSLPSSVF : 275
HpHaN_-_FUh : V------GQRTSRNP-LGALFSTNT-SAGTSLLYQLTLDALEEDDYCHFEIMLEFLHNRIHFLIGGTETYSMSTLDYSAFDPIFMIVHSGMDRLWVLWQELQKLRRKPFVAI---ECGEK-SLHEPLHPFD---YDINTIALTREHAVPDTLF : 273
CaHaN_-_FUh : A------GQRTSRNP-LEALFSTNT-SAGTSLLYQLTLDALEEDDYCQFEIMLEFFHNRIHFLIGGSETYSMSTLDYSAFDPIFMIVHSGMDRLWVLWQELQKLRRKPFVAI---ECGEK-SLHEPLHPFD---YDINTISLTRENAVPDSIF : 273
HpHb_-_FUh  : A------GKTTTRSP-LEALFSANT-SRGHTILYDLTLDALEEEDYCHFEASLEFLHNRIHFFIGGTGTYSMSTLDYSAFDPLFMVVHSGMDRIWDLWEQLQKLRHKSYTPV---DCGGH-TFDKPLHPFD-F-PAINTIALTRQNAIPELLF : 274
CaHb_-_FUh  : V------GQTTTRSP-LQALFSANT-TQGHTILYDLTLNALEEEDYCHFETSLEFLHNRVHFFIGGSGTYSMSTLDYSAFDPLFMIVHSGMDRIWDLWEGLQKLRHKPYKPV---SCAGH-KFDKPLHPFD-F-PAINTIALTRQNAIPEQLF : 274
HtH1_-_FUh  : V------QHETTRDV-NQRLFNQTK-FGEFDYLYYLTLQVLEENSYCDFEVQYEILHNAVHSWLGGTGQYSMSTLEYSAFDPVFMIHHSSLDRIWILWQKLQKIRMKPYYAL---DCAGDRLMKDPLHPFN-Y-ETVNEDEFTRINSFPSILF : 278
KLH1_-_FUh  : I------NQDTVRDV-NEAIFQQTK-FGEFSSIFYLALQALEEDNYCDFEVQYEILHNEVHALIGGAEKYSMSTLEYSAFDPYFMIHHASLDKIWIIWQELQKRRVKPAHAG---SCAGD-IMHVPLHPFN-Y-ESVNNDDFTRENSLPNAVV : 273
HtH2_-_FUh  : V------GHDTVREP-TSLIYNQPQ-IHGYDYLYYLALTTLEENNYCDFEVQYEILHNAVHSWLGGSQKYSMSTLEYSAFDPVFMILHSGLDRLWIIWQELQKIRRKPYNFA---KCAYH-MMEEPLAPFS-Y-PSINQDEFTRANSKPSTVF : 279
KLH2_-_FUh  : A------GHDTARSP-SDLLFNQPQ-LHGYDYLYYLALSTLEEDNYCDFEVHYEILHNAVHLWLGGTETYSMSSLAFSAYDPVFMILHSGLDRLWIIWQELQKLRKKPYNAA---KCAGH-MMDEPLHPFN-Y-ESANHDSFTRANAKPSTVF : 276
```

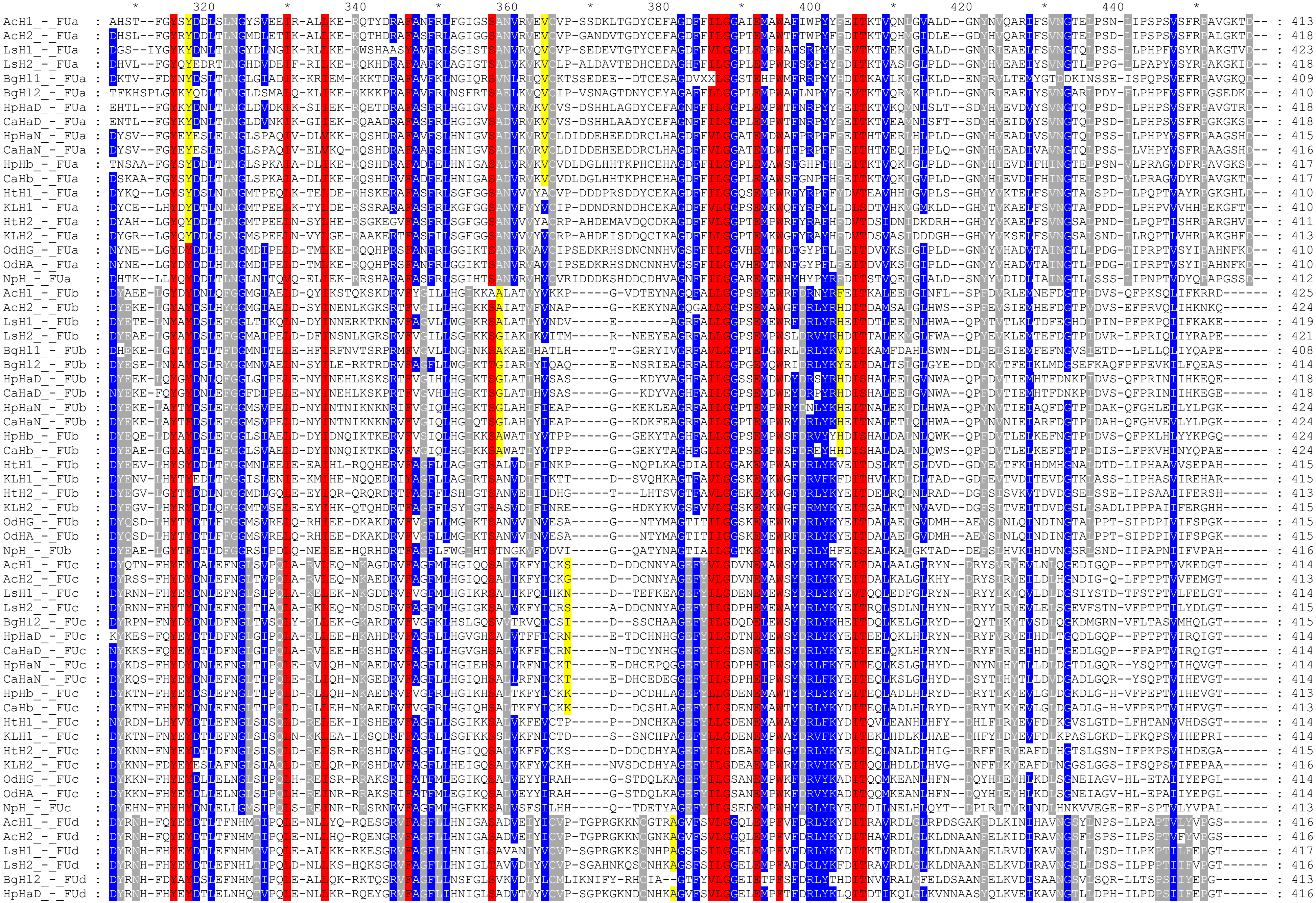

```
CaHaD_-_FUd : DYRNN-FHYEYDTLELNHLTVPQLE-SLLNR-RQEYGRVFAGFLIHNIGLSADVTVYVCVP-SGPKGKNDCNHKAGVFSVLGGELEMPFTFDRLYKLQITDTIKQLGLKVNNAASYQLKVEIKAANGTLLDPH-ILPDPSIIFVPGT------ : 416
HpHaN_-_FUd : DYRNN-LHYEYDTLEFNHLSIPQLE-ELLQS-RKRNGRVFAGFLIHNIGLSADVDVYVCVT-VGQFGEQDCNHKAGTFSVLGGETEMPFEFNRLYKQDITRTVRELGLKLDNAANFHLLVEIRAPNGSQLDPH-ILPDPSIIYIPGT------ : 415
CaHaN_-_FUd : DYRNN-LHYEYDTLEFNHLSIPQLE-ELIQS-RKRNGRVFAGFLIHNIGLSADVDVYVCVS-VGKYGEQDCDHKAATFSVLGGETEMPFEFNRLYKQDITRTVRELGLKLDYAANFHLLVEIKAPNGSQLDPH-ILPDPSIIYIPGT------ : 415
HpHb_-_FUd  : DYRNH-FDYEYDNLEFNHQSIPQLE-NLLKL-RQRKGRVFSGFLIHNIGLSADVEVSICLP-NAPDAFGGCNHKAAVFSILGGETEMPFEFDRLFRIDITKAVKDAGLEVSAAANFKLKVAIRAVNGSYLDPH-ILPDPSIIYDPGT------ : 415
CaHb_-_FUd  : DYRNH-FDYEYDNLEFNHQSIPQLE-NLLKQ-RQTKGRVFAGFLIHNIGLSADVEVSICLP-NAPDAFGSCDHKAAVFSILGGETEMPFEFDRLFRIDITKAVVDAGLEPSASANFKLKVAIRAVNGSYLDPH-TLPDPSIIYDPGT------ : 415
HtH1_-_FUd  : DYQNH-FGYKYDNLEFHHLSIPSLD-ATLKQ-RRHHDRVFAGFLLHNIGTSADITIYICLP-DGRRG-NDCSHEAGTFYILGGETEMPFIFDRLYKFEITKPLQQLGVKLH-GGVFELELEIKAYNGSYLDPH-TF-DPTIIFEPGT------ : 413
KLH1_-_FUd  : EYQNS-FGYAYDNLELNHYSIPQLD-HMLQE-RKRHDRVFAGFLLHNIGTSADGHVFVCLP-TGEHT-KDCSHEAGMFSILGGQTEMSFVFDRLYKLDITKALKKNGVHLQ--GDFDLEIEITAVNGSHLDSH-VIHSPTILFEAGT------ : 412
HtH2_-_FUd  : DYQNN-FGYKYDNLEFNHFSIPRLE-EIIRI-RQRQDRVFAGFLLHNIGTSATVEIFVCVP--TTSGEQNCENKAGTFAVLGGETEMAFHFDRLYRFDISETLRDLGIQLD-SHDFDLSIKIQGVNGSYLDPH-ILPEPSLIFVPGS------ : 411
KLH2_-_FUd  : DYQNR-FRYQYDNLQFNHFSIQKLD-QTIQA-RKQHDRVFAGFILHNIGTSAVVDIYICVE-QGGEQ--NCKTKAGSFTILGGETEMPFHFDRLYKFDITSALHKLGVPLD-GHGFDIKVDVRAVNGSHLDQH-ILNEPSLLFVPGE------ : 413
OdHG_-_FUd  : DYQNV-LHYKYDTLSFYDLTITQLD-HLIEE-RKSHDRIFAGFLLHGVQASADIHVFICVP-TSKHE-ENCAHDVGVFSVLGGKSEMPWQFASVFQYEITDQLKLLGLNQN--SHFRGVTEVTAVNGSSINSD-IFPHPTIIYVPKQ------ : 416
OdHA_-_FUd  : DYQNV-LHYKYDTLSFYDLTITQLD-HLIEE-RKSHDRIFAGFLLHGVQASADIHVFICVP-TSKYE-ENCAHDVGVFSVLGGKSEMPWQFDRVFRYEITDQLKSLGLNQN--SHFRVVTEVTAVNGSSINSD-IFPHPTIIYVPRQ------ : 416
NpH_-_FUd   : DYQNH-FHYRYDNLDFHGLSIPQLE-HELHE-RQTHDRVFVGFLLHGIQASAYVRIYICVPTQRGSSKENCNNYAGIFSILGGVTEMPWHFDRLYYYEITHALSNLGLSHR--SHFRIKTDINAVNGTHFDSH-IFPDPTISFVPAP------ : 417
AcH1_-_FUe  : EYEEH-FNYNYDHLEFVGLDIPALD-GFIKE-KQEEDRVFAGFLLKGFGSSAFVNFDIVSN--------GKSFEGGYFTVLGGSAEMPWQFDRLFKYEITDQLIAANLRFD--DNYSFNISVRLPDGTVLDSS-LIPTPSVLFKSAQ------ : 417
AcH2_-_FUe  : DYEGH-FNYRYDNLEFVGMNIPRLD-AFIKE-RRERSRVFAGFLLKGFGSSAMVTFDICTK-----GMESC-FQGGYFTVLGGAAEMPWQFDRLFKYEITDQLSEHNLRPD--DDYHFHVHVKYIDGTELDHS-LVPNPSVIIEPAS------ : 419
LsH1_-_FUe  : NYADH-FHYRYDNLEFVGLSIPQLD-AFIKE-RHEHDRVFAGFLLKGFKKSALVDFDICAN-------GNC-FPGGYFTVLGGSAEMPWQFDRLFKYEITDQLNEHKLRFD--DEYTFQIKIRAPDGTELSSD-LIGTPSVILEPFK------ : 417
LsH2_-_FUe  : NYEDH-FHYRYDDLQFVGMSIPRLD-AFIKE-KQEHDRVFAGFLLKGFGQSALVSFEICND-----DMSNC-FEGGYFTVLGGSQEMPWRFDRLYKYEITEQLEEHNLRYD--DNYHFHVHVKNIDGTMLPGK-LIPDPTVILVPAQ------ : 419
BgHl2_-_FUe : KYETN-FQYRYDNLELAGMSISQLD-AYIKG-KIKHDRVFAGFQLNGLRLSAFVDFEICTV-------RKC-TEGGYFAVLGGAEELPWQFDRLYKYDITQQLKSRALRFD--DDYEIKVHVKSMNGSQLSSS-LIASPNIIFVPSK------ : 417
HpHaD_-_FUe : AYEDH-FEYQYDSLEFVGMNIPALD-AYIKE-RQEHDRVFAGFLLHGLGTSASVNFTICDA------FKKC-FEGGHFTVLGGELEKPWQFDRLYRYPITDVLKENHIHYD--DDYHFHIHIKALNGTELDSK-LIPEPSVLFVAAH------ : 418
CaHaD_-_FUe : AYEEH-FEYQYDSLEFVGMTIPALD-AYIKE-RQEHDRVFAGFLLRGLGTSATVDFTICDA------FKKC-FDGGHFTVLGGEQEKPWQFDRLYRYPITDVLKENHIHYD--DDYHFHIHIKALNGTELDSK-LIPEPSVLFVAAH------ : 418
HpHaN_-_FUe : AYKEH-FHYKYDALEFVGMNIPTLD-AYIKE-RQEHDRVFAGFLLKGIGQSASVKFEICID-----ATGVC-FDGGEFSILGGSAEMPWQFNRLYKYEITDQLESHHVHYD--DEYHLTVHLKSINGTELDSH-LIHEPTVIYVPAK------ : 419
CaHaN_-_FUe : AYKEH-FHYKYDALEFVGMNIPTLD-AYIKE-RQEHDRVFAGFLLKGIGQSATVEFEICSD-----ATGDC-FDGGEFSILGGSAEMPWQFNRLYKYEITDQLESHHVHYD--DEYHLTVHLKSINGTELDSH-LVHEPTVIYVPAK------ : 419
HpHb_-_FUe  : DYERH-FHYRYDALEFVGMNIPTLD-AYIKE-RQEHDRVFAGFLLKGIHQSATVDFDICKG-------NNC-FEGGYFSILGGPAEMEWEFDRLYNYEITDQLKEHHVRYD--DDYNFKIQIIAPDGAILDSH-LLHEPTVSFVPAR------ : 417
CaHb_-_FUe  : YYESH-FHYRYDALEFGGMNIPTLD-AFIKE-RQEHDRVFAGFLLKGIHQSATVDFDICKG-----DTDNC-FPGGYFSILGGPAEAEWEFDRLYNYEITDQLKEHHVRYD--DDYHFKIQIKAPDGTILDSS-LLREPTVSFAPAK------ : 419
HtH1_-_FUe  : DYKK--FGYRYDSLELEGRSISRID-ELIQQ-RQEKDRTFAGFLLKGFGTSASVSLQVCRV------DHTC-KDAGYFTILGGSAEMPWAFDRLYKYDITKTLHDMNLRHE--DTFSIDVTITSYNGTVLSGD-LIQTPSIIFVPGR------ : 420
KLH1_-_FUe  : DYQK--FGYIYDTLEFAGWSIRGID-HIVRN-RQEHSRVFAGFLLEGFGTSATVDFQVCRT------AGDC-EDAGYFTVLGGEKEMPWAFDRLYKYDITETLDKMNLRHD--EIFQIEVTITSYDGTVLDSG-LIPTPSIIYDPAH------ : 419
HtH2_-_FUe  : DYRK--FGYEYDNLEFLGMSVAELD-QYIIE-HQENDRVFAGFLLSGFGGSASVNFQVCRA------DSTC-QDAGYFTVLGGSAEMAWAFDRLYKYDITETLEKMHLRYD--DDFTISVSLTANNGTVLSSS-LIPTPSVIFQRGH------ : 419
KLH2_-_FUe  : DYHR--FGYEYDSLEFVGMSVSSLH-NYIKQ-QQEADRVFAGFLLKGFGQSASVSFDICRP------DQSC-QEAGYFSVLGGSSEMPWQFDRLYKYDITKTLKDMKLRYD--DTFTIKVHIKDIAGAELDSD-LIPTPSVLLEEGK------ : 419
OdHG_-_FUe  : RYKDN-FHYEYDILDINSMSINQIE-SYIRH-QRDHDRVFAGFLLSGFGSSAYATFEICIE------GGEC-HEGSHFSVLGGSTEMPWAFDRLYKIEITDILSDMNLAFD--SAFTIKTKLVAQNGTELPAS-ILPEATVIRIPPS------ : 420
OdHA_-_FUe  : RYKDN-FHYEYDILDINSMSINQIESSYIRH-QKDHDRVFAGFLLSGFGSSAYATFEICIE------GGEC-HEGSHFAVLGGSTEMPWAFDRLYKIEITDVLSDMHLAFD--SAFTIKTKIVAQNGTELPAS-ILPEATVIRIPPS------ : 421
NpH_-_FUe   : NYKDH-FDYEYDMLEFSGMTIPQLE-DYIKE-SLHHERIFAGFLLHGIGQSANVDFEVCDN------QNHC-EDAGYFSILGGSLEMPWAFNRLYRYDITHTLETMHLNYD--DNFHINIHIHDMAGNDLDSH-LLQKATILRQPAA------ : 421
AcH1_-_FUf  : DYEH--LGYSYDDLSIGGYDLDGLE-QVIAD-RQSKARVFAGFLLKGVKTSGSVVINICLR------NNVC-NYAGRFNLLGGPTEMAWAFDRLFTYDITSALEQSGINPEDVFDAEALFTLDIKVFDVEGHALPVSSVLPEPTIIYKAAV-- : 419
AcH2_-_FUf  : DHEK--LGYAYDDLTIGGYDLEGLE-DLITA-RRSHARVFAGFLLKGAKTSGSAVIDICRP------NNEC-AYGGRFNVLGGPLEMDWAFNRLFKFDITRALEEAGLHPEDVFDSEALFTLRVKIFNAEGQAVALKSVMPEPTIIFEPAH-- : 419
LsH1_-_FUf  : NHEE--LGYHYDNLNIGGYNLDQLE-QLIAQ-HQSRPRVFAGFLLKGIKTSGSVVLNVCKG-------SQC-TYAGRFNLLGGPSEMPWAFDRLYKKDITWALEAADIKPESVFDADSLFTLDVKVFDVEGHAINVKSVFPTPSIIYDPAK-- : 417
LsH2_-_FUf  : DYEE--LGYAYDTLEIGGYSLEQIE-EIIAD-NKKTARVYAGFLLKGIKTSGSVIIKACKD-------SEC-AFAGRFNLLGGPTEMPWAFDRLFKADITWALRNLGLTADDVTDVDSHFHLEVDITDVKGRSLNRKSVMPEPTIIFRPAE-- : 418
BgHl2_-_FUf : NHDL--LGYQYDHLKIGGHSVNDLE-YLIND-KKMKNRILAGFLLKSVNTSVSVKLHVCNC----QECSVC-IYAGGFSLLGGPTERTWAFDHLFKKDITHAVEEAGIEVSDSADFFLDVKVYDRAGKPMKDRSVFPTPSIILVPTKVKCL-- : 420
HpHaD_-_FUf : NHED--LGYAYDNFNIGGYNLDELE-KLIHD-RQIKPRIFAGFLLKGIKTSGSVNLKVCKF-------EEC-TPAGFFNLLGGPLEMPWAFDRLYKKDITWAIAKLGLNPDDIHNTDSGFKLEVQAFNVEGTALPLSQVMPTPSVIYKPAL-- : 418
CaHaD_-_FUf : NHED--LGYAYDNFNIGGYDLDQLE-KLIHD-RQIKPRIFAGFLLKGIKTSGSINLKVCKL-------EEC-TSGGFFNLLGGPLEMPWAFDRLYKKDITWAIANIGLNPDDIHKTDSGFRLEVQAFNVEGTALPLSQVMPTPSIIYKPAF-- : 416
HpHaN_-_FUf : DHEN--LGYTYDHLKVGGYDLGQLE-ELIHD-NQQHARVFAGFLLHGIKTSGSVKIKLCQG-------DKC-TSAGQFNLLGGPLESPWAYNRLYKRDITQYLEDLNLHPEDVFDPILQVHLEVEVRDVQDHLLVAQDVLPQPTIIFDPPH-- : 418
CaHaN_-_FUf : DHEN--LGYTYDNLNVGGYSLDQLE-ELIHD-NQQHARVFAGFLLHGIKTSGSVKIKLCQG-------DKC-TSAGQFNLLGGPLESPWAYNRLYKRDITQYLAALNLHPEDVFDPTLQVHLEVEVRDVQDHVLEAQNVLPQPTIIFDPPH-- : 418
HpHb_-_FUf  : DHEN--LGYGYDNLNIGGYTIEELE-ALIHD-REKEDRVFVGFLLKGIGTSGVVTINICLR------SGNC-TFSGRFSLLGGPLEAPWAYDRLYKKEITHYLADIHIEPK--DVLNASVYLKIEVDDVEGRTLSPKAVFPNPTIIFKAGH-- : 417
CaHb_-_FUf  : DHED--LGYGYDNLNIGGYTIDELE-ELIHE-RQKEDRVFAGFLLKGIHTSGVVTINVCVK------SGNC-TFAGRFSLLGGPLEAPWAYDRLYKREITQYLGEIHIKPR--DILNANVYLKIEVDDVEGRTLSPKAVFPHPTLIFEPGH-- : 417
HtH1_-_FUf  : DYED--LGYNYDNLEISGLNLNEIE-ALIAK-RKSHARVFAGFLLFGLGTSADIHLEICKT------SENC-HDAGVIFILGGSAEMHWAYNRLYKYDITEALQEFDINPEDVFHADEPFFLRLSVVAVNGTV-IPSSHLHQPTIIYEPGE-- : 417
KLH1_-_FUf  : KYEL--LGYRYDNLEIGGMNLHEIE-KEIKD-KQHHVRVFAGFLLHGIRTSADVQFQICKT------SEDC-HHGGQIFVLGGTKEMAWAYNRLFKYDITHALHDAHITPEDVFHPSEPFFIKVSVTAVNGTV-LPASILHAPTIIYEPGL-- : 417
HtH2_-_FUf  : DYET--LYYSYDNLEIGGRNLDQLQ-AEIDR-SRSHDRVFAGFLLRGIGTSADVRFWICRN------ENDC-HRGGIIFILGGAKEMPWSFDRNFKFDITHVLENAGISPEDVFDAEEPFYIKVEIHAVNKTM-IPSSVIPAPTIIYSPGE-- : 416
KLH2_-_FUf  : DFQD--LFYTYDNLEIAGMNVNQLE-AEINR-RKSQTRVFAGFLLHGIGRSADVRFWICKT------ADDC-HASGMIFILGGSKEMHWAYDRNFKYDITQALKAQSIHPEDVFDTDAPFFIKVEVHGVNKTA-LPSSAIPAPTIIYSAGE-- : 417
OdHG_-_FUf  : DDNK--LGYKYDNLEFHGMNIDQLE-NAIHK-QQNKDRVFASFLLFGIKTSADVHLKLCKD-------ETC-EDAGVVFILGGDNEMPWHFDRTYKKDITHVLHQMHIPLEDLYVHGSTILLEVEIETVDGKV-LDSSSLPAPSMIYVPAK-- : 414
OdHA_-_FUf  : DYNK--LGYKYDDLEFHGMNIDQLE-NAIHK-TQNKDRVFASFLLFGIKTSADVHLKLCKD-------ETC-EDAGVVFVLGGDNEMPWHFDRTYKMDITNVLHKMHIPLEDLYVHGSTIHLEVEIESVDGKV-LDSSSLPVPSMIYVPAK-- : 414
NpH_-_FUf   : NYDD--LGYSYDNLLFDGLTITELE-EKIHS-RQSHPRVFAGFLLHAIGTSADVHVSVCHH------GNNC-TSAGLFFILGGKHEMPWKYDRLFRYDITDAVTKAGFSPEDVFNDNLLYKIHTEINAVNGSH-IDSHIMHEPTLIFVPGR-- : 416
AcH1_-_FUg  : DYDR--FNYQYDNLNFHGLTISELN-DLLEK-RKEEDRVFAEFLLAGFGGSGDVVFNLCSE-------EEC-AFAGTFAVLGGAIEMPWAFDRLFKYDVTNVFRKLNLRPD--DVYHFEVKIVAVNGTELYPG-LIRPPSVQFVPGVK----- : 403
AcH2_-_FUg  : DYDR--FNYQYDNLNFHGLTISELN-DLLEK-RKEEDRIFAEFLLAGFGGSADVIFNLCSE-------TEC-TFAGTFAVLGGAIEMPWAFDRLFKYDVTNVFMKLNLRPD--DVYHFEVEIVAVNGTHLDSG-LIRPPSVQFVPGVK----- : 402
LsH1_-_FUg  : DYDR--LNYQYDNLNFHGLTISQLN-DLLEK-RKEEDRIFAEFLLRGFSASADVIFKLCDE------KGHC-EFAGTFAVLGGLLEMPWAFDRLFKYDVTNVFNKLNLRTD--SDYHFDVEIKAINGTVLDSN-LIRSPSVQFVPGVK----- : 403
LsH2_-_FUg  : DYDR--FNYQYDNLNFHGLTISQLN-DLLEK-RIEEDRIFAEFLLIGFGASADVTFQLCDA------ENHC-AFAGTFAVLGGPLEMPWALDRLFKYDVTNVFNQLHLKPD--SKYHFEVKIVAVNGTELDST-IIRPPTVQFVPGVK----- : 403
HpHaD_-_FUg : NYDR--LNYQYDDLNFHGLSISELN-DVLER-RKEKARIFAEFLLHGIGASADVTFDLCDS------HDHC-EFAGTFAILGGPLEHPWAFDRLFKYDVTDVFSKLHLRPD--SEYHFNIHIVSVNGTELDSH-LIRSPTVQFVPGVK----- : 403
CaHaD_-_FUg : NYDR--LNYQYDDLNFHGLSISELN-DVLER-RKEKARIFAEFLLHGLGASADITFDLCDS------HDHC-EFAGTFAVLGGPLEHPWAFDRLFKYDVTDVFSKLHLRPD--SEFHFNIHIVAVNGTELDSH-LIRSPTVQFVPGVK----- : 403
HpHaN_-_FUg : NADS--LHYQYDNLNFHGHSIAELE-EILQH-RQEEDRVFAEFLLHGLKTSADVSFDLCDE------RGHC-EFAGTFAILGGYLEMDWEFDRLFRYDVTNVFNKLHLQPD--SDYHFVDHITAVNGTELDSQ-LIRPPSVHFVPGVK----- : 403
CaHaN_-_FUg : SSDR--LHYQYDNLDFHGHSIVELE-EILQH-RQEEDRVFAEFLLHGLKTSADVTFDLCDE------RNHC-EFAGTFAILGGDLEMDWEFDRLFKYDVTNVFNKLHLQPD--SAYHFVDHITAVNGTELDSN-LIRPPSVHFVPGVK----- : 403
HpHb_-_FUg  : NYEQ--LHYHYDNLNFHGDTISQLV-NVIDE-RKSHDRIFAEFLLHSIGTSADVTFELCDE------HNHC-EFAGTFAILGGPLEMAWSTDRLFRYDVTDVFEKLHLQAD--SEYHFVDHIVAVNGTELDSH-LIKPPSVRFVPGTK----- : 403
CaHb_-_FUg  : NYEQ--LHYQYDNLNFLGHTISELV-DIIEE-RKSHDRIFAEFLLHSIGTSADVTFELCDE------RNRC-AFAGTFAILGGPLEMGWANDRLFKYDVTDVFQKLHLQAD--SEYHFVDHIVAVNGTELDSH-LIHPPSVRFVPGTR----- : 403
HtH1_-_FUg  : EYNR--LSFQYDNLIFHGYSIPELD-RVLEE-RKEEDRIFAAFLLSGIKRSADVVFDICQP------GHEC-VFAGTFAILGGELEMPWSFDRLFRYDITKVMKQLHLRHD--SDFTFRVKIVGTDDHELPSD-SVKAPTIEFEPGV------ : 404
KLH1_-_FUg  : EYSR--FNFQYDNLRFHGMTIKKLE-HELEK-QKEEDRTFAAFLLHGIKKSADVSFDVCNH------DGEC-HFAGTFAILGGEHEMPWSFDRLFRYDITQVLKQMHLEYD--SDFTFHMRIIDTSGKQLPSD-LIKMPTVEHSPGG------ : 404
HtH2_-_FUg  : DYER--FNYQYDTLSFHGKSIPELN-DLLEE-RKREERTFAAFLLRGIGCSADVVFDICRP------NGDC-VFAGTFAVLGGELEMPWSFDRLFRYDITRVMNQLHLQYD--SDFSFRVKLVATNGTELSSD-LLKSPTIEHELGA------ : 404
KLH2_-_FUg  : DYER--LNYQYDTLTFHGHSISELD-AMLQE-RKKEERTFAAFLLHGFGASADVSFDVCTP------DGHC-AFAGTFAVLGGELEMPWSFERLFRYDITKVLKQMNLHYD--SEFHFELKIVGTDGTELPSD-RIKSPTIEHHGGD------ : 403
OdHG_-_FUg  : DYHK--LGYDYDNLNFHGMTIPELE-EHLKE-IQHEDRVFAGFLLRTIGQSADVNFDVCTK------DGEC-TFGGTFCILGGEHEMFWAFDRLFKYDITTSLKHLRLDAH--DDFDIKVTIKGIDGHVLSNK-YLSPPTVFLAPAKTTH--- : 407
OdHA_-_FUg  : DYHK--LGYDYDNLNFHGMTIPELE-EHLKE-IQHEDRVFAGFLLRTIGQSADVNFDVCTK------DGEC-TFGGTFCILGGEHEMFWAFDRLFRYDITTSLKHLHLDAH--DDFDIKVTIKGIDGHVLPNK-YLSPPTVFLAPAKTN---- : 406
NpH_-_FUg   : DYHQ--LGYEYDTLEFHGHNIPQLA-VYLDS-YHEHDRVFAGFLLHGIKRSADVNFDICSL------NGDC-TNAGTFALLGGEHEMPWAFDRLFKYDITKPLQNLRLHST--DDFSINIHIVAINGEELDSH-LIPTPSIIFSPGRGAQH-- : 404
```

```
AcH1_-_FUh   : DYAH--LGYEFDNLELNGHDVNDLN-NIINR-LRDQDRVYLAFNNCGKQGS--FAFDVHFETA-----ENNVVPLGRFYVLGGEREMPWCFERVFKYDVTEVLQANNVDIH--KPVKIGGQLFRYDGEHVQNF-TTYYATFIERPAGVDYD-- : 412
LsH1_-_FUh   : DSER--LGYKYDKLELNGNSVEEIN-NIIKN-LHHQERIFVGILAFGHQKS--LTIHISLIND-----NDEAFDGGNIHILGGEKEMPWAYERIMKLDVTEAIRKGKISTD--HAVKARFTSTDYQGNLNHQD-TDYAIIVERHAEQDY---- : 410
LsH2_-_FUh   : DSER--FGYEFDKLELNGHDVSELN-GIIQR-LRGNTRYFLGFVLYGVRQT--VGVEVTLISN------KKRYPAGNFYVLGGEKEMPWAYERIAKYDVTDVFHKAKLSDD--KPVVGEFYSSLYNGQA-NNN-VTEEVIVVEKPSDVNYD-- : 410
BgHl1_-_FUh : DHRR--LGYKFDKLNLNVFSLKDLE-DKIKL-QKSKNRTYAGLMLRGVKNS--VTLEVYLQDN----------QVGTVNILGGPNEKPWVFERPYKIDVTDAMKGAQLTTD--KPVKLHLKTGTYDGSSSSEK-DM-EVFIIERPSGSHHD-- : 397
HpHaD_-_FUh : DYTH--FGYEFDKLELNGHDVQGID-DIIHN-LRHGNRVYLGFVLFGQQSS--LEYKIDLIDD-----AGQAHTAGSFHLLGGEREMPWAYERLFKYDVTDVAKQFGITTD--HPIKVKVTSTYYNGEPHQEY-TDEIVAVERHADTDYD--- : 411
CaHaD_-_FUh : DYTH--FGYEFDKLELNGHDVQGID-NVIHN-LRHGNRVYLGFVLFGQQSS--LEYKIDLIDD-----EGQAHTAGSFHLLGGEREMPWAYERLFKYDVTDVAKKFGITTD--HPIKVKVTSTYYNGEPHQEY-TDEIVAVERHADTDYD--- : 411
HpHaN_-_FUh : DHHL--LGYEYDTTEISEHDAAEVL-EIIRR-RHSETRVYLGGAAYGHGQS--FRTAAWVLND-----AGEEFDAGTNFLLGSSKEMPWANEILWKFDITTAVHQAGVSTD--KNIKFHFKIETYNGTLFASE-SAYAIFLVREAYTDY---- : 408
CaHaN_-_FUh : DHHH--LQYDYDTTEISGHDAAEVL-EIIRR-RHSETRVYLGGAAQGYGES--YRTETWVLND-----AGEKFSVGTNFLLGSAKEMPWSNEILWKFDITNAVRQAGVSTD--KNIKFHFIAETYNGTLHHDQ-VAYGIFVIREAYTDYLT-- : 410
HpHb_-_FUh   : EHDR--LGYEYDNLRISGYDVEQVN-EILKK-RHAETRIYYSEAAAGYGVS--VSYEVWVLDE-----EGHEFPADTGYILGSSKEMPWTYEILFKYDITDAIHKANVSLD--HPVKFKWRTVNYDGTLIEEA-SEYGIVVVREAKTDYL--- : 410
CaHb_-_FUh   : EHDN--LGYEYDNLRISGYDVGQVN-EILKK-RHAETRIYYSEAASGFGVS--VSYEVWVLDD-----EGHEIPADTGFVLGSSKEMPWTYEILFKYDITDAFNKANLSLD--HPVKFKWRMVNYDGTFLEEA-SEYGIVVVREANTDYL--- : 410
HtH1_-_FUh   : DHYR--FNYEYDNMRIRGQDIHELE-EVIQE-LRNKDRIFAGFVLSGLRIS--ATVKVFIHSK---NDTSHEEYAGEFAVLGGEKEMPWAYERMLKLDISDAVHKLHVK-D--EDIRFRVVVTAYNGDVVTTR--LSQPFIVHRPAHVAHD-- : 415
KLH1_-_FUh   : DSHR--FNYKYDNLNLHGHNIEELE-EVLRS-LRLKSRVFAGFVLSGIRTT--AVVKVYIKSG----TDSDDEYAGSFVILGGAKEMPWAYERLYRFDITETVHNLNLT-D--DHVKFRFDLKKYDHTELDAS-VLPAPIIVRRPNNAVFD-- : 410
HtH2_-_FUh   : DQHK--FGYHYDNLNVRGHSIQELN-TIIND-LRNTDRIYAGFVLSGIGTS--ASVKIYLRTD------DNDEEVGTFTVLGGEREMPWAYERVFKYDITEVADRLKLSYG--DTFNFRLEITSYDGSVVNKS--LPNPFIIYRPANHDYD-- : 414
KLH2_-_FUh   : DSHK--FNYHYDNPDVRGNSIQEIS-AIIHD-LRNQPRVFAGFVLSGIYTS--ANVKIYLVRE-----GHDDENVGSFVVLGGPKEMPWAYERIFKYDITEVANRLNMHHD--DTFNFRLEVQSYTGEMVTHH--LPEPLIIYRPAKQEYD-- : 412
```
