# Supplementary material for: The evolution of hemocyanin genes in Tectipleura: a multitude of conserved introns in highly diverse gastropods
Source: BMC Ecol Evol. 2021 Mar 4;21:36. doi: 10.1186/s12862-021-01763-3 (PMC7931591; doi:10.1186/s12862-021-01763-3)

Additional file 7

Movie files: Distribution of splice sites positions in a 3D model of a hemocyanin FU. Splice site positions (colored spheres) of all FUs of hemocyanins from (A) Tectipleura and (B) Tectipleura, Lepetellida, Octopodoidea and *Nautilus pompilius* are shown as an overlay in the 3D-reconstruction of an *Octopus dofleini* FU-g (PDB-ID: 1JS8). They are distributed all over the tertiary structure of functional units. Different colors indicate splice site frequencies: In (A): unique splice site positions which occur in only one FU of Tectipleura are colored in red. Those positions that are identical in multiple splice sites are blue (splice sites in 2 FUs) or yellow (3FUs). (B) shows splice site positions that can be found once (red) or twice (blue) in different hemocyanins of different molluscan species. Occurrence in multiple FUs of one hemocyanin gene structure are not encoded in coloring of (B).

(A)

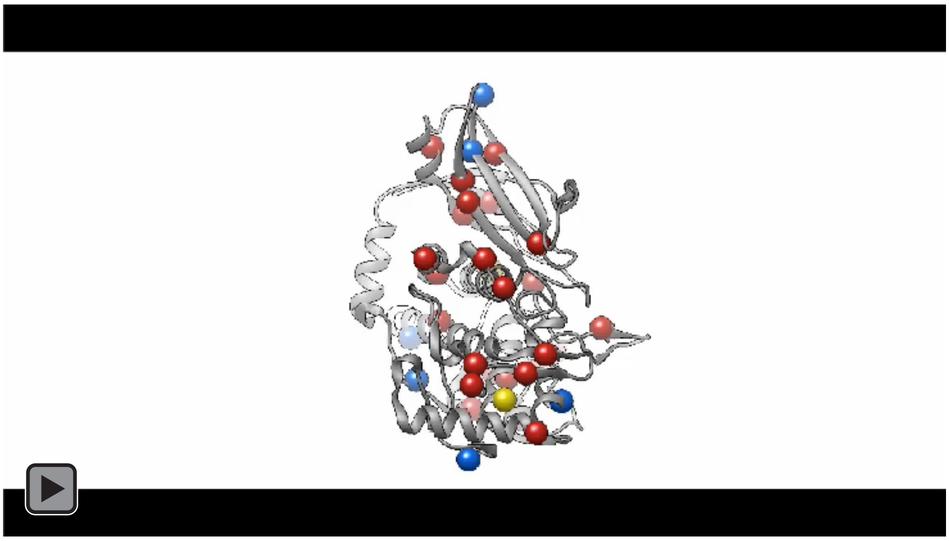

(B)

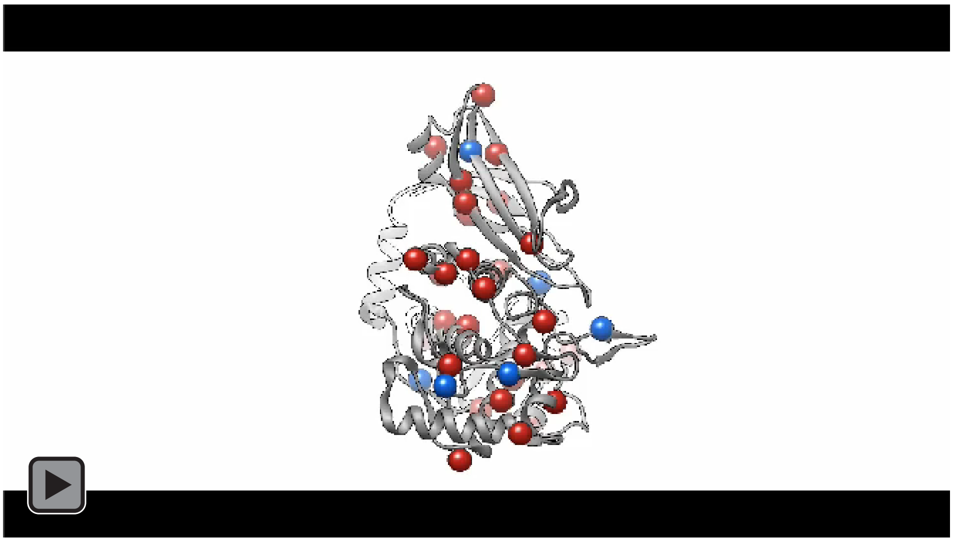

Supplement: Supplementary file 7 — Additional file 7. Movie files. Distribution of splice sites positions in a 3D model of a hemocyanin FU. [file 12862_2021_1763_MOESM7_ESM.pdf]
