# Supplementary material for: The evolution of hemocyanin genes in Tectipleura: a multitude of conserved introns in highly diverse gastropods
Source: BMC Ecol Evol. 2021 Mar 4;21:36. doi: 10.1186/s12862-021-01763-3 (PMC7931591; doi:10.1186/s12862-021-01763-3)
Supplement: Supplementary file 8 — Additional file 8: Table S3. Primer description. [file 12862_2021_1763_MOESM8_ESM.pdf]

Additional file 8

Table S3: Primer description. (A) All primers applied for confirmation of hemocyanin exon-intron structure in *Helix pomatia* and *Lymnaea stagnalis* are listed. **(B)** Primer list applied for sequencing longer fragments via Sanger Sequencing.

(A)

Primer	***5'-3' sequence***	***Tm [°C]***
AN FU-b E6 S	AAC CCT ACA AGG CCC ACT GC	62.5
AN FU-b E6 AS	TTG ATG CCG TGG AGC TGG A	59.5
AN FU-a E5E6 S	GTG CCA ACT CAC CCC TCT GT	62.5
AN FU-a E5E6 AS	ACG CCG ATG TTG TGT AGA CTG A	62.1
AN FU-d E3E4 S	TCC GCC TTC GAC CCT GTC TT	62.5
AN FU-d E3E4 AS	TTC CGG GTC ACT TCA TCA GTG T	62.1
AD FU-c E3E4 S	CGG TGA GGT CGG TCA GGA GT	64.5
AD FU-c E3E4 AS	AGA CCT CAC TGC CTC CAA CC	62.5
AD FU-c E6E7 S	GGA GGA ACA CAA AGC CCA TGA T	62.1
AD FU-c E6E7 AS	CGA AGT AGC GGT CAT TGT AGC GA	64.6
B FU-h E6E8 S	TGG ATG AAG AAG GCC ACG AGT	61.2
B FU-h E6E8 AS	TTG TAC GAT TCA TTG ACT GCC	57.5
Lymnaea_stagnalis_FU-c_Exon1 S	GCT GAC ACC ATC AAA ATC CGT A	60.1
Lymnaea_stagnalis_FU-c_Exon2 AS	GCA GGA TTT CTT TAC CTC AGC A	60.1

(B)

Primer	***5'-3' sequence***
AD FUc E3E4	
SSequ1	ACGGTTTTCGTTTCACAC
ASSequ1	CTACTCCTAACTCAAGCCT
BFUh E6-E8	
SSequ1	GACAAGAACGTTTGCAGTG
SSequ2	TTATACAGGCGACCAAAAGC
SSequ3	GACCGGTGTATGGGCTGAA
ASSequ1	CTGTAACCAGCAAAACAGG
ASSequ2	GGCCAGACGGACACATAATA
ASSequ3	GCCGTTGAGTGAGCCTTCT

Table S4: Overview of PCR programs applied using the two different enzyme amplification systems. **A)** displays the doubtful sequence parts confirmed by LD PCR with the SuperFii system for HpHαD FU-c exon 3-4 and HpHβ FU-h exon E6-E8. **B)** shows all fragments confirmed by applying the Advantage polymerase system. For HpHαN FU-b exon E6 and HpHαN FU-a exon E5-E6 a two-step protocoll was used whereas for HpHαN FU-d exon E3 (60°C A_{TM}), HpHαD FU-c exon 6/7 (60°C A_{TM}) and the hygrophila-specific intron in LsH1 (58°C A_{TM}) a three-step protocol was necessary.

A)

Step	*Temp [°C]*	*Time*	*Temp [°C]*	*Time*	*Cycles*
	aD FUc Exon 3-4		***B FUh Exon E6-E8***		
Denaturation	98	5 min	98	5 min	1x
Denaturation	98	10 sec	98	10 sec	
Annealing	72	3 min	55.5	10 sec	30x
Extension			72	3 min	
Final Extension	72	5 min	72	5 min	1x

B)

Two-Step protocol				***Three-Step protocol***			
Step	*Temp [°C]*	*Time*	*Cycles*	*Step*	*Temp [°C]*	*Time*	*Cycles*
Denaturation	95	5 min	1x	Denaturation	95	5 min	1x
Denaturation	95	30 sec		Denaturation	95	30 sec	
Annealing	68	1/2 min	30x	Annealing	T	30 sec	30x
Extension				Extension	68	1/2 min	
Final Extension	68	5 min	1x	Final Extension	68	5 min	1x
